# Flat trend of high caesarean section rates in Peru: A pooled analysis of 3,376,062 births from the national birth registry, 2012 to 2020

**DOI:** 10.1016/j.lana.2022.100293

**Published:** 2022-06-17

**Authors:** Hugo G. Quezada-Pinedo, Kim N. Cajachagua-Torres, Wilmer Cristobal Guzman-Vilca, Carla Tarazona-Meza, Rodrigo M. Carrillo-Larco, Luis Huicho

**Affiliations:** a The Generation R Study Group, Erasmus MC, University Medical Center Rotterdam, Rotterdam, the Netherlands; b The Department of Paediatrics, Erasmus MC, University Medical Center Rotterdam, Rotterdam, the Netherlands; c Centro de Investigación en Salud Materna e Infantil and Centro de Investigación para el Desarrollo Integral y Sostenible, Universidad Peruana Cayetano Heredia, Lima, Peru; d Facultad de Medicina “Alberto Hurtado”, Universidad Peruana Cayetano Heredia, Lima, Peru; e Sociedad Científica de Estudiantes de Medicina Cayetano Heredia (SOCEMCH), Universidad Peruana Cayetano Heredia, Lima, Peru; f CRONICAS Centre of Excellence in Chronic Diseases, Universidad Peruana Cayetano Heredia, Lima, Peru; g Program in Human Nutrition, Department of International Health, The Johns Hopkins Bloomberg School of Public Health, Baltimore, MD 21205, USA; h Centre for Non-Communicable Diseases Research and Training, Johns Hopkins University, Baltimore MD, USA; i Universidad Cientifica del Sur, Lima, Peru; j Department of Epidemiology and Biostatistics, School of Public Health, Imperial College London, London, UK

**Keywords:** Caesarean rates, Birth registry, Newborn, Maternal and child health, Low- and middle-income countries

## Abstract

**Background:**

National and subnational C-section rates are seldom available in low- and middle-income countries to guide policies and interventions. We aimed to describe the C-section rates at the national and subnational levels in Peru (2012-2020).

**Methods:**

Based on the Peruvian national birth registry, we quantified C-section rates at the national, regional and province levels; also, by natural regions (Coast, Highlands, and Amazon). Using individual-level data from the mother, we stratified the C-section rates by educational level, healthcare insurance and provider. Ecologically, we studied the correlations between C-section rates and human development index (HDI), altitude above sea level, proportion of the population living in poverty and proportion of rural population.

**Findings:**

C-section rate in Peru decreased slightly from 2012 (39·7%) to 2020 (38·0%). A widening gap of C-section rates was observed through the study years among the Coast that showed higher rates and the other natural regions that showed lower rates. The rates in most of the 25 regions showed a flat trend, particularly in the last four years and some provinces showed a very low rate. The rates were highest in mothers with higher education and in users of private health insurance. Higher HDI, health facility located at lower altitude, lower poverty and urbanization were positively correlated with higher C-section rates.

**Interpretation:**

C-section rates in Peru are above the international recommendations. Large differences by natural region, provinces and women socioeconomic status were found. Further efforts are needed to achieve the recommended C-section rates.

**Funding:**

Academy Ter Meulen grant of the Academy Medical Sciences Fund of the Royal Netherlands Academy of Arts & Sciences (KNAWWF/1327/TMB202116), Wellcome Trust (214185/Z/18/Z), Fogarty (D43TW011502).

**Research in context**

**Evidence before this study**

We conducted a search in PubMed on January 5^th^, 2022 using the following search terms: “caesarean section AND developing countries”, without data and language restriction. From 1,003 hits, we identified 2 studies that evaluate the worldwide caesarean section (C-section) rates. Both studies evaluated the C-section rates in a global level, one of the studies referred to the 1990-2014 period and the other the 2010-2018 period. These studies only included country-level data and did not contain any geographical nor socioeconomic sub-national differences within each country. Worldwide analysis might hide within countries differences. This hampers efforts to identify places where interventions are needed to keep C-section rates levels suggested by international health organizations.

**Added value of this study**

From our understanding, this is the only observational study in Peru assessing the C-section rates in Peru between 2012 and 2020, with a detailed exploration at national, regional, and provincial levels. In addition, we analysed C-section rates by natural region and by available socioeconomic variables. The rates did not change substantially between 2012 and 2020, and they remained well above the international recommended levels over time. At the subnational level, larger differences were distinguished, with the highest rates being reported in the Coast and in areas with better socioeconomic indicators.

**Implications of all the available evidence**

This study uniquely shows that C-section rates in Peru are well above the international recommendations. A comprehensive evaluation is needed to identify the drivers of these high rates and reasons why the rates have not decreased, including potential health system factors, health workforce and user-related factors. The fact that the rates did not substantially change in this study period, calls to revise the available policies and interventions to reduce the frequency of C-sections. The subnational differences may imply that region-specific policy actions are needed, fostered, or articulated by the national government.

Alt-text: Unlabelled box

## Introduction

Clinical guidelines have clear indications for caesarean section (C-section) and are aimed to prevent perinatal complications for both mothers and newborns. Performing a C-section, when it is not clearly indicated, has not shown benefits and has been associated with maternal and offspring complications and may incur in unnecessary health expenditures.^1^ The rate of C-sections have progressively increased worldwide and the trends suggest they will increase in the current decade.^2^, ^3^, ^4^, ^5^ Whether all the C-sections were absolutely necessary, is debatable. Latin America and the Caribbean appears to be the region with the highest C-section rates in the world; in this region in 2015, 44% of all births were C-sections.^2^ In Peru, the C-section rate doubled (from 12·7% to 26·5%) between 2000 and 2012.^6^ However, these estimates only considered public hospitals and showed the epidemiology of C-sections ten years ago. It is largely unknown whether these estimates are still valid today and if they reflect the epidemiology of C-sections at the national and subnational levels in Peru. A previous study using the Peruvian Demographic and Family Health Surveys (ENDES, in Spanish) that C-section rates were 21.4% in 2009 and 34.5% in 2018%.^29^^,^^30^ Although ENDES is a representative national survey, the use of secondary data from a survey might have resulted in reduced precision and accuracy of the estimated data at national and subnational levels.^29^^,^^30^ Due to the implementation of some health policies and interventions at the regional level (first administrative division in Peru), a subnational characterization of C-section rates could provide detailed information to policymakers and local health authorities. In terms of maternal and child health, Peru and other countries in Latin America show large within country inequalities.^7^ This also calls for subnational data/evidence-driven policies. Furthermore, identifying modifiable characteristics (e.g., education level of users or healthcare insurance provider) and geographic patterns associated with high C-section rates could aid to select groups and places where interventions to monitor C-section rates are urgently needed. Leveraging on a national birth registry, this study aimed to describe trends in C-section rates at the national level and by geographical and sociodemographic variables in Peru between 2012 and 2020.

## Methods

### Data sources

We used information from the Online Live Birth Certificate Registration System (*Sistema de Registro del Certificado de Nacido Vivo*, in Spanish). This registry covers the whole Peruvian territory and all healthcare systems (e.g., public and private services).^8^^,^^9^ Implemented in March 2012, it includes information of the newborn recorded immediately after birth (e.g. mode of delivery, sex, birthweight, and gestational age); the registry also records information of the mother (e.g. age, education, and health insurance). The coverage of this system has improved over the time, including 12% of all projected births in 2012, 37% in 2013, 53% in 2014, 72% in 2015, 80% in 2016, 84% in 2017 and 88% in 2018.^10^ Data can be accessed upon request from the Ministry of Health: https://www.minsa.gob.pe/portada/transparencia/solicitud/. Additionally, for all provinces in Peru we collected information on proportion of people living in poverty, proportion of people living in rural areas, human development index (HDI), and altitude above sea level from the National institute of Statistics and Computing.^11^

### Study setting

Peru is an upper-middle income country located in South America, hosts 32,971,846 inhabitants, and has a gross domestic product of US$ 202·0 billion in 2020.^12^ Peru is geographically divided in three natural regions: Coast, Highlands and Amazon.^12^ The country is politically and administratively divided in 25 regions (equivalent to states), which are further divided in 196 provinces (equivalent to counties).^13^ In addition, the 25 regions in Peru can be grouped according to their geographic location. Those regions along the Pacific Ocean are “Coast’; those regions surrounding the Andes are “Highlands”; and those regions in the Amazon rainforest are “Amazon”. The main healthcare providers include the SIS (Seguro Integral de Salud, in Spanish) run by the Ministry of Health and covering 64% of the population; ESSALUD (*Seguro Social de Salud*, in Spanish) run by the Ministry of Labour and provides care for 29% of the population; and the private health sector.^14^ SIS has a national/state-funded (Beveridge) model and it mainly targets the segment of the population with limited financial resources while ESSALUD is a social insurance (Bismark) model that provides coverage to formally employed population.^14^^,^^15^ Of note, there could be overlap between the three healthcare providers.

### Study population

For this analysis, we included all women-child pairs from the registration system between 2012 and 2020. Of 3,394,988 live births, we conducted a complete-case analysis. To ensure data quality, the following cleaning criteria and plausibility ranges were applied: a) observations with birthweight below 500 g or above 5,500 g; b) observations with gestational age outside the range of 22-44 weeks; c) observations with delivery mode missing; and d) observations with maternal age younger than 9 years were excluded. When summarizing the results at the province level only, those provinces with fewer than 30 births were further excluded.

### Definitions

The prevalence or rate of C-section is expressed as a percentage (%) where the numerator was the number of births by C-section and the denominator was the total number of live births. The C-section rates are described in relation to time and geographic levels, and also in relation to sociodemographic characteristics: maternal education attainment (none/primary/incomplete secondary, complete secondary and any higher education), health insurance provider (SIS, ESSALUD and private/out-of-pocket health expenditure), and category of the health care facility where the birth was registered, whereby category I (lowest capacity) correspond to medical centers, categories II and III (highest capacity) correspond to hospitals.^16^

### Statistical analysis

First, C-section rates were summarized along with 95% confidence intervals (95%CI). We used Clopper-Pearson exact CI to estimate the confidence intervals using ProCIs package in R software. Maps and time trend plots were used to characterize the spatial and temporal patterns of the C-section rates. Second, we used equiplots to illustrate the differences in C-section rates according to maternal education, health insurance, and category of the healthcare facility. Third, scatterplots were used to explore potential relationships between C-section rates and selected determinants at the province level (ecological analysis); these scatterplots also showed the Kendall's tau-b correlation coefficients. All statistical analyses were performed with R version 4·0·2 (R foundation, Vienna, Austria). Our study has been reported according to the Strengthening the Reporting of Observational studies in Epidemiology (STROBE) guidelines.^17^

### Ethics

We used anonymized data that was retrieved from open access websites and the study was conducted according to the guidelines laid down in the Declaration of Helsinki. The study was approved by the Research Ethics Committee of Universidad Peruana Cayetano Heredia (UPCH), Lima, Peru (SIDISI 207860).

### Role of funding source

The funders of the study had no role in study design, data collection, data analysis, data interpretation, or writing of the report. All authors had full access to the data in the study. All authors collectively had final responsibility for the decision to submit for publication and vouch for the data accuracy. The authors alone are responsible for the opinions in the manuscript, which do not necessarily represent those of their institutions.

## Results

### Study population characteristics

Between 2012 and 2020, 3,394,988 births were originally recorded in the national registry. After applying our selection criteria, data from 3,376,062 births were included in the analysis (*Supplementary Figure* 1). Overall, 48·9% of the newborns were girls; the mean gestational age was 38·7 weeks (standard deviation (SD) = 1·7); the mean maternal age was 27·8 years (SD = 6·9); 11·2% of all births occurred in private health services; and the C-section rate was 36·5% (Table 1).

**Table 1:** Characteristics of the study population by year.

Year	2012	2013	2014	2015	2016	2017	2018	2019	2020	2012-2020
**Sample size (n)**	72,352	213,536	306,256	415,109	457,300	478,235	491,990	482,916	458,368	3,376,062
**Newborn sex, girls (%)**	48·6	48·7	48·7	48·8	48·8	48·9	48·9	48·8	49·0	48·8
**Gestational age [mean (standard deviation)], weeks**	38·8 (1·8)	38·8 (1·8)	38·8 (1·7)	38·8 (1·7)	38·7 (1·7)	38·7 (1·7)	38·7 (1·7)	38.7 (1·7)	38.7 (1·7)	38·7 (1·7)
**Gestational age [10^th^ – 50^th^ – 90^th^ percentile], weeks**	37-39-40	37-39-40	37-39-40	37-39-40	37-39-40	37-39-40	37-39-40	37-39-40	37-39-40	37-39-40
**Maternal age [mean (standard deviation)], years**	26·7 (6·9)	26·9 (6·9)	27·3 (6·9)	27·5 (6·9)	27·7 (6·9)	27·8 (6·9)	28·0 (6·9)	28·1 (6·9)	28·3 (6·9)	27·8 (6·9)
**Mode of delivery [% (95% CI)]**										
Vaginal	60·2 (59·8- 60·5)	62·8 (62·6-63·0)	64·4 (64·2-64·6)	64·3 (64·1-64·4)	65·3 (65·1-65·4)	64·6 (64·5-64·7)	62·8 (62·6-62·9)	61·9 (61·8-62·0)	61·9 (61·7-62·0)	63·4
C-section rate	39·7 (39·3-40·0)	37·0 (36·8-37·2)	35·4 (35·3-35·6)	35·6 (35·4-35·7)	34·6 (34·5-34·8)	35·3 (35·1-35·4)	37·1 (37·0-37·2)	38·0 (37·8-38·1)	38·0 (37·8-38·1)	36·5 (36·4-36·5)
Instrumental	0·2 (0·2-0·2)	0·2 (0·2-0·2)	0·2 (0·2-0·2)	0·2 (0·2-0·2)	0·1 (0·1-0·1)	0·1 (0·1-0·1)	0·1 (0·1-0·1)	0·1 (0·1-0·1)	0·1 (0·1-0·2)	0·1 (0·1-0·1)
**Maternal education (%)**										
Any higher education	21·0	23·8	27·2	29·1	30·3	31·0	33·3	34·5	35·0	31·0
Complete secondary	45·6	39·8	37·2	35·8	34·6	34·5	34·4	34·5	35·3	35·6
Incomplete secondary/any primary/none	33·4	36·4	35·6	35·1	35·1	34·5	32·3	31·0	29·8	33·4
**Health insurance (%)**										
SIS^a^	66·4	76·1	71·4	69·3	71·1	71·3	69·6	70·0	69·2	70·5
ESSALUD^b^	0·4	3·9	12·7	19·6	20·1	20·3	20·3	19·4	16·9	17·4
Private and out-of-pocket health expenditure	32·7	19·4	15·2	10·5	8·2	7·8	9·1	9·5	12·6	11·2
Others	0·5	0·6	0·7	0·6	0·6	0·7	1·0	1·1	1·3	0·9
Human development index*	0·74	0·75	0·76	0·76	0·77	0·77	0·77	0·78	-	-
**Population living in rural areas (%)**†	23·2	23·0	22·8	22·6	22·5	22·3	22·1	21·9	21·7	-
**Population living in poverty (%)**§	25·8	23·9	22·7	21·8	20·7	21·7	20·5	20·2	30·1	-

a SIS: Seguro Integral de Salud in Spanish.

b ESSALUD: *Seguro Social de Salud* in Spanish.

⁎ Reference: United Nations Development Programme. Human Development Reports [Internet]. [cited 2022 Mar 5]. Available from: https://hdr.undp.org/en/indicators/137506#.

† Reference: World Bank. Rural population (% of total population) - Peru | Data [Internet]. The World Bank | Data. [cited 2022 Mar 5]. Available from: https://data.worldbank.org/indicator/SP.RUR.TOTL.ZS?locations=PE.

§ Reference: Carhuavilca D, Sanchez A. Evolución de la Pobreza Monetaria 2009-2020 [Internet]. Lima: Instituto Nacional de Estadística e Informática; 2020. Available from: https://www.inei.gob.pe/media/MenuRecursivo/publicaciones_digitales/Est/pobreza2020/Pobreza2020.pdf.

### Geographical trends

**Figure 1:**
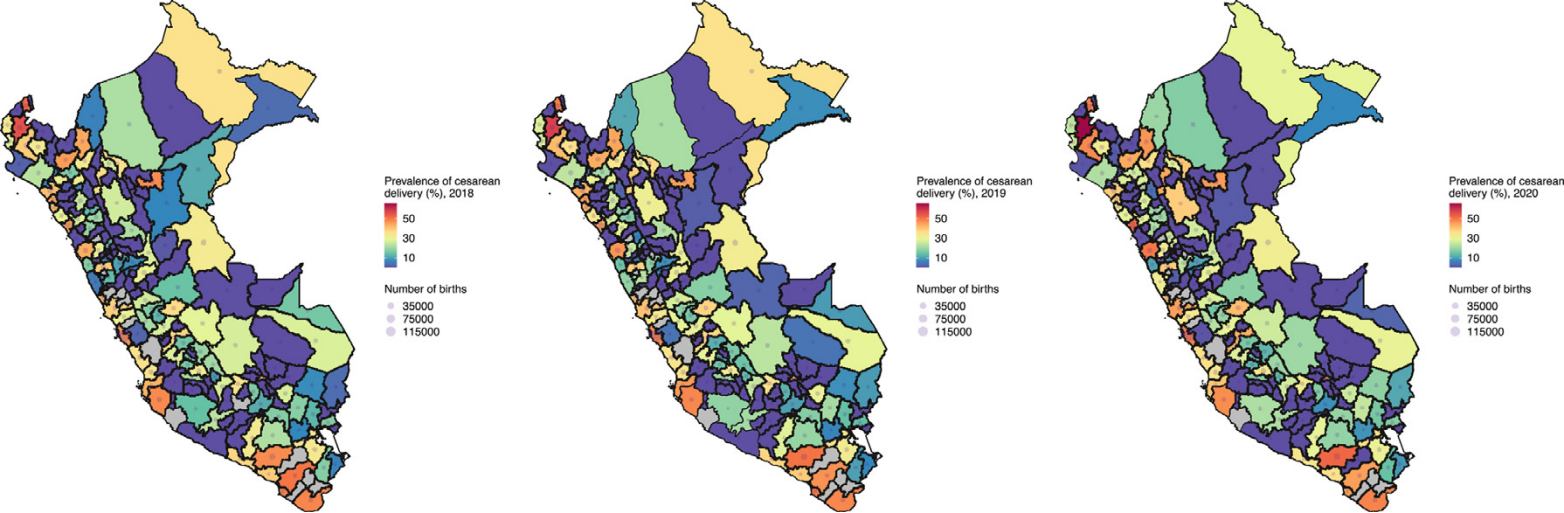
Geographic and temporal profiles of caesarean section rates in Peru between 2018 and 2020. Colours indicate the caesarean section rates and the size of the grey bubbles indicates the number of registered births, i.e., the denominator for the rate estimates. Both colours and bubbles are at the province level. The dark black lines indicate the boundaries of the regions. NB Peru is divided in 25 regions and these into 196 provinces. Maps for all years are presented as Supplementary Materials. The provinces with the highest caesarean section rates in 2020 were: Sullana (Coast), Arequipa (Highlands), Trujillo (Coast), Santa (Coast), Lima (Coast), Tumbes (Coast), Tacna (Coast), Piura (Coast), Ica (Coast), and Bagua (Amazon). The provinces with the lowest (non-zero) caesarean section rates in 2020 were: Viru (Coast), Chupaca (Highlands), Datem del Marañon (Amazon), San Ignacio (Amazon), San Marcos (Highlands), Calca (Highlands), Requena (Amazon), Huacaybamba (Highlands), Ferreñafe (Coast), and Huarochiri (Highlands).

Across the years, the rate of C-section was highest in the Coast and lowest in the Amazon (Figure 1 and *Supplementary Table* 1). In 2020, eight out of the ten provinces with the highest C-section rates were in the Coast, while five out of the ten provinces with the lowest (non-zero) rates were in the Highlands. A similar profile was observed throughout the study period. Moreover, some provinces showed very low C-section rates, namely Viru (Coast) that had 0·1%, Chupaca (Highlands) and Datem del Marañon (Amazon) that had 0·2% each in 2020 (Table 2).

**Table 2:** Provinces with the lowest and highest caesarean section rates, Peru: period 2012-2020.

	2012	2013	2014	2015	2016	2017	2018	2019	2020
**Coast caesarean section rates (%)**									
Lowest	Chiclayo 0·8 (0·0-2·9)	Contralmirante Villar 2·1 (0·6-7·4)	Ferreñafe 3·1 (2·0-4·6)	Ferreñafe 6·4 (4·8-8·3)	Ferreñafe 7·2 (5·5-9·3)	Palpa 1·3 (0·1-7·2)	Viru 0·2 (0·1-0·7)	Sechura 0·2 (0·0-0·9)	Viru 0·1 (0·0-0·5)
Highest	Piura 49·9 (47·9-51·8)	Santa 56·1 (51·2-60·8)	Santa 51·9 (50·3-53·5)	Tumbes 55·6 (54·0-57·2)	Tumbes 52·3 (50·7-53·9)	Tumbes 50·2 (48·6-51·8)	Sullana 54·7 (53·5-55·9)	Sullana 57·2 (56·0-58·3)	Sullana 64·4 (63·2-65·6)
**Highlands caesarean section rates (%)**									
Lowest	Ayabaca 4·5 (2·1-9·6)	Acobamba 7·2 (5·2-9·9)	Sanchez Carrion 0·6 (0·0-3·4)	Yungay 0·3 (0·0-1·5)	Ayabaca 0·1 (0·0-0·8)	Ayabaca 0·1 (0·0-0·7)	Sihuas 0·4 (0·0-2·2)	Huancabamba 0·4 (0·1-1·2)	Chupaca 0·2 (0·0-1·1)
Highest	Arequipa 47·8 (44·6-51·0)	Otuzco 47·5 (40·0-55·2)	Huaraz 44·1 (39·2-49·2)	Arequipa 45·2 (44·5-45·9)	Arequipa 45·7 (45·0-46·3)	Arequipa 46·3 (45·6-47·0)	Arequipa 47·1 (46·4-47·8)	Arequipa 49·8 (49·0-50·5)	Arequipa 51·6 (50·9-52·4)
**Amazon caesarean section rates (%)**									
Lowest	Padre Abad 1·2 (0·0-4·1)	Padre Abad 3·7 (2·4-5·7)	Loreto 0·6 (0·0-3·1)	San Ignacio 0·2 (0·0-0·9)	San Ignacio 0·1 (0·0-0·7)	San Ignacio 0·4 (0·1-1·1)	Atalaya 0·1 (0·0-0·8)	Datem del Marañon 0·2 (0·0-1·1)	Datem del Marañon 0·2 (0·0-1·0)
Highest	San Martin 57·4 (47·7-66·6)	Condorcanqui 48·5 (32·5-64·8)	San Martin 53·7 (51·9-55·5)	San Martin 51·5 (50·0-53·0)	San Martin 49·3 (47·8-50·7)	San Martin 47·1 (45·8-48·4)	San Martin 47·1 (45·8-48·5)	San Martin 45·0 (43·7-46·4)	Bagua 46·1 (43·2-49·0)

Results are presented as rate estimates along with their 95% confidence intervals (95% CI)**.**

In regions with both Coast and Highlands territory, the C-section rates increased from the Highlands to the Coast. For example, in Ancash in 2020, the Santa province (Coast) had the highest C-section rate (49·4% (CI 95%: 48·3%-50·5%)) yet the Recuay province (Highlands) had the lowest (non-zero) rate (2·3% (CI 95%: 0·8%-6·6%). Even though both provinces are in the same region, the rate was 21-fold higher (49·4% vs 2·3%) in Santa than in Recuay.

### Time trends

**Figure 2:**
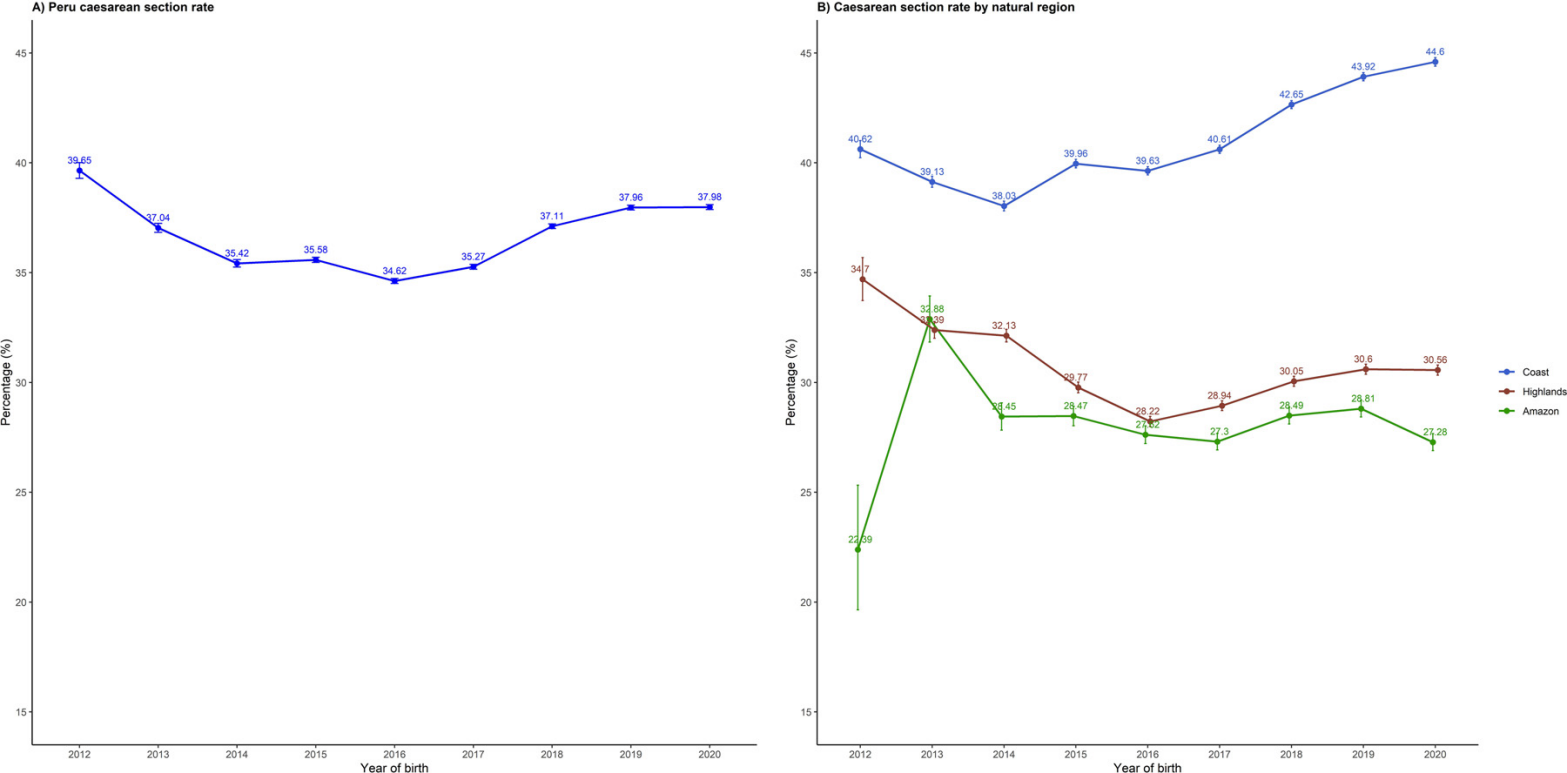
National time trends of caesarean section rates, in Peru between 2012 and 2020.

At the national level C-section rates were high and did not change substantially over time (Figure 2**A**), decreasing slightly from 39·7% in 2012 to 38·0% in 2020. Across the study period, the C-section rates in Peru ranged between 34·6% to 39·7%. However, we identified two relevant periods: a period of around 5% decrease from 2012 to 2016 (from 39.7% to 34.6%), and a period of around 3% increase from 2016 to 2020 (from 34.6% to 38.0%). When exploring the C-section rates by natural regions, the Coast had the highest C-section rates across the study period, followed by the Highlands and then the Amazon (Figure 2**B**). Moreover, C-section rate in the Coast increased while it decreased in the rest of the natural regions showing 14% and 17% difference with the Highlands and the Amazon, respectively, in 2020. C-section rates in most of the 25 regions in Peru showed a stagnant trend, particularly in the last four years. The top three largest increases were observed in Lambayeque (15·5% in 2014 and 33·5% in 2020), Amazonas (26·7% in 2014 and 36·3% in 2020) and Lima (39·5% in 2014 and 47·6% in 2020). Decreasing trends were less often observed. The top three largest decreases were observed in San Martin (52·9% in 2014 and 32·2% in 2020), Apurimac (34·5% in 2014 and 25·0% in 2020) and Ancash (46·0% in 2014 and 37·4% in 2020) *Supplementary Figure* 2-3).

### Equiplots

**Figure 3:**
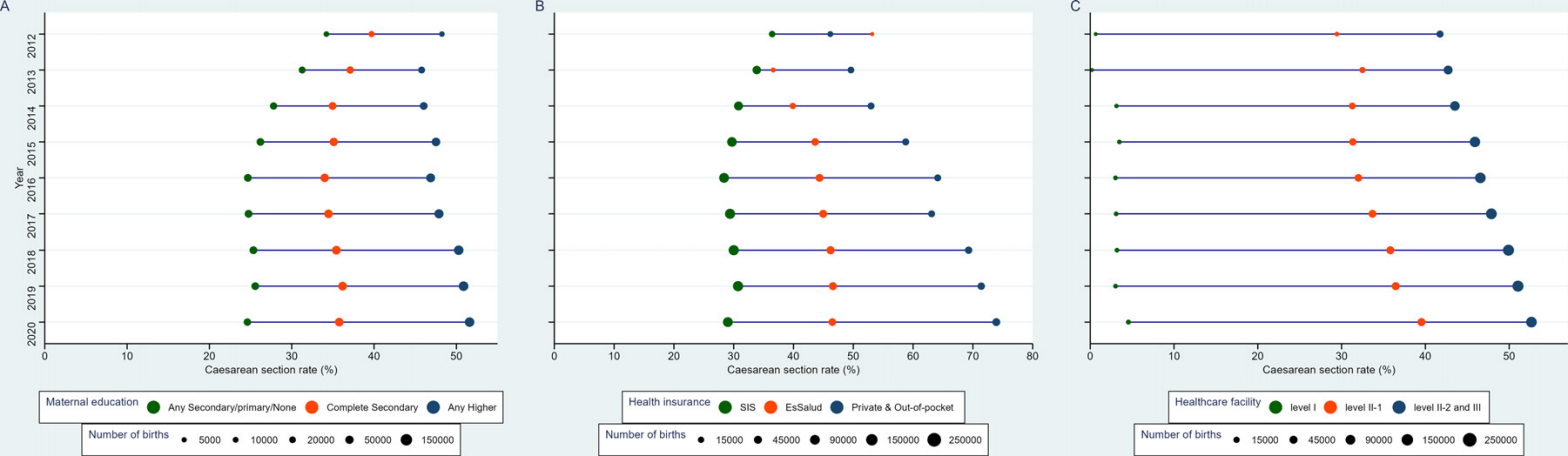
Equiplots of caesarean section rates according to maternal education attainment (A), health insurance provider (B) and level of the medical center (C). The size of the bubbles is relative to the number of births.

C-section rates were higher in the group of mothers with higher education (Figure 3**A**). Between 2012 to 2020, C-section rates amongst mothers with incomplete secondary or lower education decreased from 34·2% to 24·6%, while in mothers with complete secondary education decreased from 39·7 to 35·8%. Conversely, in mothers with any higher education C-section rates increased from 48·6% to 53·1% in the same period. C-section rates were the highest in mothers with a private health insurance and lowest in those with a public health insurance (e.g. SIS) (Figure 3**B**). This difference increased over time (Figure 3**B**). In private/out-of-pocket health expenditure settings, C-section rates increased from 46·2% in 2012 to 73·9% in 2020. In those with SIS insurance, C-section rates decreased from 36·4% to 29·0% in the same period. The C-section rates were highest in healthcare facility with a higher category, where C-section rates increased from 41·7% to 52·7% (Figure 3**C**).

### Correlations

**Figure 4:**
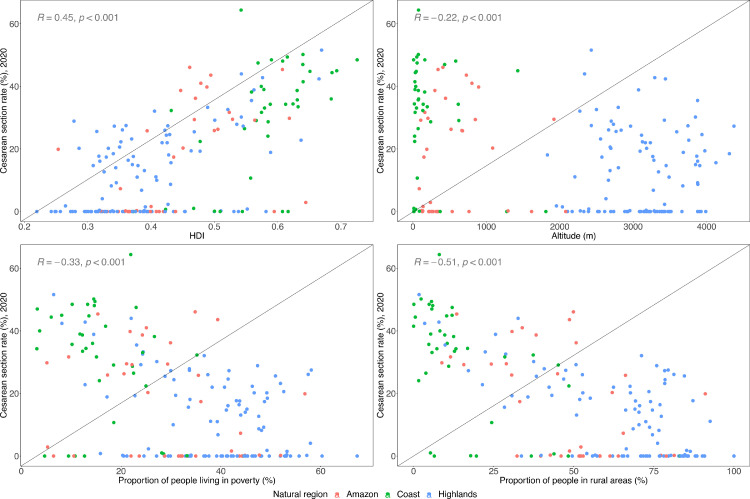
Scatterplots of caesarean section rates with human development index (HDI), altitude above sea level, poverty, and rurality. Each dot represents one province, and the colours show the region to which they belong. The Kendall's tau-b correlation coefficient is presented in each plot.

At the province level (ecological analysis), provinces with higher HDI showed higher C-section rates. Conversely, provinces at high altitude, those with higher proportions of people living in poverty and those living in rural areas had lower C-section rates (Figure 4).

## Discussion

### Main results

Our study shows that in Peru the national C-section rate in 2020 was 38%; that is, 2·5-fold times higher the international recommendations.^5^^,^^18^ We did not observe substantial changes in the time trends for C-section rates at the national and subnational levels. The national prevalence over the analysis period ranged between 35% and 40%, being consistently above the 10-15% recommended by the World Health Organization as the optimal rate for only medically necessary caesarean deliveries.^5^^,^^18^ There were substantial differences in the C-section rates at the subnational level through the study years. A widening gap was observed among natural regions, where the Coast showed an increase in C-section rates while the Amazon and the Highlands showed a decrease. A similar gap was observed on maternal education level and kind of health insurance during the study period. Provinces in the Coast always had higher C-section rates than provinces in the Highlands and in the Amazon. Importantly, large differences were observed among natural regions through the study years. While in the Coast the C-section rates raised, in the Amazon and in the Highlands they actually decreased, configuring a widening gap profile between the Coast and the rest of the country. Likewise, a widening gap depending on the maternal education level and the kind of health insurance of the mother was observed in the study period, with better educated mothers and those with private health insurance showing higher C-section rates. We additionally found provinces with alarming low C-section rates, where interventions are needed to bring C-section rates closer to the international recommendations. Our findings highlight a mixed subnational pattern of C-sections in Peru, one characterized by unacceptably high rates, particularly prevalent in better-off Coast provinces with higher access to public health services, most C-sections being most likely unnecessary and even harmful to women and newborns, and the other one mostly prevalent in the poorer Amazon and Highlands regions, with lower levels of access to private health services, where the C-section rates are consistently lower than in Coast provinces across the study period, including even provinces with alarming low rates, which could mean that a substantial proportion of deliveries needing C-section cannot actually benefit from this intervention, risking as a consequence the lives of mothers and newborns.

### Strengths and limitations

Strengths of our study include the use of a national birth registry with information covering the period 2012-2020 and including both public and private hospitals throughout Peru. This rich database allowed the description of national and subnational trends in C-section rates. Nonetheless, limitations need to be addressed. Misclassification bias due to differences in registration procedures across provinces cannot be excluded. This is a common limitation when analysing national registries and should not substantially modify the time and geographic trends. Notably, data entry in the national registration system followed standard procedures and was performed by health professionals using national guidelines.^19^, ^20^, ^21^, ^22^

We did not present results for all provinces throughout the observation period. Early in the implementation of the national birth registry not all provinces had access to the registry though in the last five years we included most of the country. Even in recent years when the national birth registry included almost all provinces in Peru, it is still possible that some births were missed. For example, births occurring at home and in remote areas may have been missed. We argue that missed births from the national registry were very few and should not have introduced bias to our results. Of note, the national birth registry complements the online registration with manual entries for births occurring where internet connection is not available. Kendall's tau-b correlations are not the ideal metric to quantify the association between two variables. We used Kendall's tau-b correlations as a hypothesis-generating approach and to help identifying possible linear relationships in the scatterplots. Thus, they should not be interpreted as the magnitude of the association, let alone as implicating casual associations.

### Potential explanations

The C-section rates in Peru were consistently well above the international recommendations and showed a marginal decrease during the study period. In the same lapse, the number of hospitals and gynaecology services also increased.^13^ This could have led to greater access to C-sections and thus more C-sections performed explaining why the C-section rates were consistently high.

We observed reductions of C-section rates in few regions: San Martin, Apurimac and Ancash. We cautiously speculate that national guidelines and other regulations aimed to promote vaginal delivery (e.g. the norm to standardize and protect users of vertical delivery), might have had some effect in these regions, but little effect in other regions where C-section rates did not change or even increased.^23^, ^24^, ^25^ Further research is needed to understand the drivers of C-section rates at the national and subnational levels. Equiplots showed a positive correlation between C-section rates and maternal education. C-section rates were higher amongst mothers with higher education. This is consistent with previous evidence in other low- and middle-income settings.^26^, ^27^, ^28^, ^29^ Similarly, and also consistent with the available evidence, C-section rates were higher amongst mothers with access to private health insurance (including those who directly paid for the procedure).^30^, ^31^, ^32^, ^33^ We hypothesize that, mothers with higher socioeconomic status, better educated, private health insurance holders and those able to afford out-of-pocket health expenditures, may have the greater access to C-section services. In this line, mothers and practitioners could prefer a C-section for convenience reasons, and because it is apparently less painful and easier to plan.^26^^,^^32^, ^33^, ^34^, ^35^, ^36^, ^37^, ^38^, ^39^ Whether all these C-sections were indeed needed is debatable and deserves further evaluation.

### Public health implications

Our findings are of particular relevance to low- and middle-income countries (LMICs), where two extreme situations can coexist in terms of C-sections, that is too little, too late (TLTL) and too much, too soon (TMTS).^28^ Previous research has rightly focused on TLTL C-sections, but as epidemiological patterns in LMICs change and access to health facilities increase, it is increasingly important to recognize that TMTS C-sections become a threat to maternal and foetal health, by simultaneously increasing costs and often concentrating disparities and abuse.^28^ Moreover, C-section rate is considered a key indicator of maternal healthcare at population level and a way to assess respectful maternity care.^28^ The World Health Organization recommends interventions to reduce unnecessary C-sections targeted at different levels: 1) women; 2) healthcare professionals; and 3) healthcare organizations, facilities or systems.^5^ Several interventions to reduce the unnecessary use of the C-section deliveries have been proposed. These include clinical interventions such as routine induction of labour and external cephalic version; and non-clinical interventions including financial incentives for health professionals, policies or legislatively imposed clinical guidelines.^35^^,^^36^^,^^40^, ^41^, ^42^, ^43^, ^44^, ^45^, ^46^, ^47^, ^48^, ^49^ Our results identify areas (e.g., provinces in the Coast) where these interventions may be needed to reduce C-section rates.

Although national guidelines with clear indications for when a C-section delivery is required have been introduced in Peru since 2004,^23^, ^24^, ^25^ C-section rates have remained high over the years. Our results provide new information for future surveillance and monitoring frameworks of C-section indicators in Peru, with detailed national and subnational trends. Stakeholders can use our results to advocate for the revision and update of available guidelines and policies. Our results may suggest that these policies may not be effective given the consistent high C-section rates nationally and sub-nationally. The differences observed between regions may imply that region-specific policies and interventions are needed, fostered, or articulated by the national government. Additionally, more research on the potential drivers of C-section rates or on the consequences of low and high C-section rates is needed.

## Conclusion

Peru has not experienced substantial changes in the C-section rates at the national and subnational levels between 2012 and 2020. During this period, C-section rates remained high and above internationally recommended levels. A widening gap of C-section rates was observed through the study years among the Coast that showed higher rates and the other natural regions that showed lower rates. Women of higher socioeconomic status, better educated and those with higher access to public health insurance showed higher C-section rates over time. Further comprehensive and locally adapted interventions are urgently needed to reduce the high C-section rates in Peru, while further efforts are warranted in areas with low rates that are below the recommended standards.

## Contributors

Conceptualization: all authors; data curation: HGQP, KNC-T and WCG-V; formal analysis: HGQ-P, KNC-T and WCG-V; writing original draft: HGQP and KNC-T; writing review & editing: HGQ-P, KNC-T, WCG-V, RMC-L, CT-M and LH. All authors approved the submitted version.

## Data sharing statement

All data herein analysed are open access and can be requested from the Ministry of Health in Peru: https://www.minsa.gob.pe/portada/transparencia/solicitud/frmFormulario.asp, and from the National institute of Statistics and Computing http://iinei.inei.gob.pe/microdatos/. We provide the data herein analysed and the analysis code of data preparation, scatterplots and maps as supplementary materials to this paper.

## Editor note

The *Lancet* Group takes a neutral position with respect to territorial claims in published maps and institutional affiliations.

## Declaration of interests

The authors declare no competing interests.

## Appendix Supplementary materials

Image, application 1

Image, application 2

## References

[1] Lumbiganon P, Laopaiboon M, Gülmezoglu AM (2010). Method of delivery and pregnancy outcomes in Asia: the WHO global survey on maternal and perinatal health 2007-08. Lancet.

[2] Boerma T, Ronsmans C, Melesse DY (2018). Global epidemiology of use of and disparities in caesarean sections. Lancet.

[3] Betran AP, Ye J, Moller A-B, Souza JP, Zhang J. (2021). Trends and projections of caesarean section rates: global and regional estimates. BMJ Global Health.

[4] Betrán AP, Ye J, Moller A-B, Zhang J, Gülmezoglu AM, Torloni MR. (2016). The increasing trend in caesarean section rates: global, regional and national estimates: 1990-2014. PLoS One.

[5] Opiyo N, Kingdon C, Oladapo OT (2020). Non-clinical interventions to reduce unnecessary caesarean sections: WHO recommendations. Bull World Health Organ.

[6] Tapia V, Betran AP, Gonzales GF. (2016). Caesarean section in peru: analysis of trends using the robson classification system. PLoS One.

[7] Ronsmans C, Holtz S, Stanton C. (2006). Socioeconomic differentials in caesarean rates in developing countries: a retrospective analysis. Lancet.

[8] Ministerio de Salud del Peru (MINSA) (2021). https://www.minsa.gob.pe/cnv/.

[9] Curioso WH, Pardo K, Loayza M. (2013). Transforming the Peruvian birth information system. Revista peruana de medicina experimental y salud publica.

[10] Ministerio de Salud del Peru (MINSA) (2019). Establecimientos de Salud Implementados..

[11] Instituto Nacional de Estadistica e Informatica (INEI) (2020). http://iinei.inei.gob.pe/microdatos/.

[12] The World Bank (2020).

[13] Instituto Nacional de Estadistica e Informatica (INEI) (2020).

[14] Alcalde-Rabanal JE, Lazo-González O, Nigenda G. (2011). The health system of Peru. Salud Publica Mex.

[15] Wang H, Switlick K, Ortiz C, Zurita B, Connor C. (2012).

[16] Ministerio de Salud (MINSA). Resolucion Ministerial N° 076-2014/MINSA, resolucion ministerial que aprueba la guia de categorizacion de establecimientos de salud. 2014.

[17] Vandenbroucke JP, von Elm E, Altman DG (2007). Strengthening the reporting of observational studies in epidemiology (STROBE): explanation and elaboration. PLoS Medicine.

[18] Betran AP, Torloni MR, Zhang J (2015). What is the optimal rate of caesarean section at population level? A systematic review of ecologic studies. Reprod Health.

[19] Registro Nacional de Identificación y Estado Civil (RENIEC). Resolucion Gerencial N° 001-2012/GOR/RENIEC, que aprueba el formulario de nacido vivo de emisión manual y en línea.

[20] Ministerio de Salud (MINSA). Resolución Ministerial N° 148-2012/MINSA, resolucion ministerial que aprueba la directiva administrativa que establece procedimiento para el registro de del certificado de nacido vivo en todos los establecimientos de salud. 2012.

[21] MInisterio de Salud (MINSA). Resolución Ministerial N° 766-2010/MINSA, que aprueba la directiva administrativa N° 166 MINSA/OGEI-V.01 “Procedimiento para el flujo y calidad de los formularios de hechos vitales del nacido vivo y de defunción”. 2010.

[22] Seguro Social de Salud (ESSALUD). Resolución de Gerencia General N° 1436 GG EsSalud 2013 - Sistema CNV, que dispone la implementación del registro en linea del certificado de nacido vivo en los centros asistenciales de las redes asistenciales del seguro social de salud (ESSALUD). 2013.

[23] Ministerio de salud (MINSA). Resolucion Ministerial 518-2016/MINSA, resolucion ministerial que aprueba la norma tecnica en salud para la atencion del parto vertical. 2016.

[24] Ministerio de Salud (MINSA). Resolución Ministerial N° 695-2006/MINSA, resolución ministerial que aprueba la guía técnica para la atención de las emergencias obstétricas según capacidad resolutiva y sus 10 anexos. 2006.

[25] Ministerio de Salud (MINSA). Resolución Ministerial N° 668-2004-MINSA, resolución ministerial que aprueba guías nacionales de atención integral de la salud sexual y reproductiva. 2004.

[26] Béhague DP, Victora CG, Barros FC. (2002). Consumer demand for caesarean sections in Brazil: informed decision making, patient choice, or social inequality? A population based birth cohort study linking ethnographic and epidemiological methods. BMJ.

[27] Manyeh AK, Amu A, Akpakli DE, Williams J, Gyapong M. (2018). Socioeconomic and demographic factors associated with caesarean section delivery in Southern Ghana: evidence from INDEPTH Network member site. BMC Pregnancy Childbirth.

[28] Zgheib SM, Kacim M, Kostev K. (2017). Prevalence of and risk factors associated with cesarean section in Lebanon — a retrospective study based on a sample of 29,270 women. Women Birth.

[29] Wang H, Frasco E, Takesue R, Tang K. (2021). Maternal education level and maternal healthcare utilization in the Democratic Republic of the Congo: an analysis of the multiple indicator cluster survey 2017/18. BMC Health Serv Res.

[30] Vallejo MC, Shapiro RE, Lilly CL, Nield LS, Ferrari ND. (2021). The influence of medical insurance on obstetrical care. J Healthc Risk Manag.

[31] Hoxha I, Syrogiannouli L, Braha M, Goodman DC, da Costa BR, Jüni P. (2017). Caesarean sections and private insurance: systematic review and meta-analysis. BMJ open.

[32] D'Souza R, Arulkumaran S. (2013). To ‘C’ or not to ‘C’? Caesarean delivery upon maternal request: a review of facts, figures and guidelines. J Perinat Med.

[33] Murray SF. (2000). Relation between private health insurance and high rates of caesarean section in Chile: qualitative and quantitative study. BMJ.

[34] Huang K, Tao F, Faragher B (2013). A mixed-method study of factors associated with differences in caesarean section rates at community level: The case of rural China. Midwifery.

[35] Chen I, Opiyo N, Tavender E (2018). Non-clinical interventions for reducing unnecessary caesarean section. Cochrane Database Syst Rev.

[36] Betrán AP, Temmerman M, Kingdon C (2018). Interventions to reduce unnecessary caesarean sections in healthy women and babies. Lancet.

[37] Shirzad M, Shakibazadeh E, Hajimiri K (2021). Prevalence of and reasons for women's, family members’, and health professionals’ preferences for cesarean section in Iran: a mixed-methods systematic review. Reprod Health.

[38] Long Q, Kingdon C, Yang F (2018). Prevalence of and reasons for women's, family members', and health professionals' preferences for cesarean section in China: a mixed-methods systematic review. PLoS Med.

[39] Colomar M, Opiyo N, Kingdon C (2021). Do women prefer caesarean sections? A qualitative evidence synthesis of their views and experiences. PLoS One.

[40] Dodd JM, Crowther CA, Hiller JE, Haslam RR, Robinson JS. (2007). Birth after caesarean study–planned vaginal birth or planned elective repeat caesarean for women at term with a single previous caesarean birth: protocol for a patient preference study and randomised trial. BMC Pregnancy Childbirth.

[41] Crowther CA, Dodd JM, Hiller JE, Haslam RR, Robinson JS. (2012). Planned vaginal birth or elective repeat caesarean: patient preference restricted cohort with nested randomised trial. PLoS Med.

[42] Middleton P, Shepherd E, Crowther CA. (2018). Induction of labour for improving birth outcomes for women at or beyond term. Cochrane Database Syst Rev.

[43] Brown HC, Paranjothy S, Dowswell T, Thomas J. (2008). Package of care for active management in labour for reducing caesarean section rates in low-risk women. Cochrane Database Syst Rev.

[44] Hofmeyr GJ, Kulier R. (2012). External cephalic version for breech presentation at term. Cochrane Database Syst Rev.

[45] Dodd JM, Crowther CA, Huertas E, Guise JM, Horey D. (2004). Planned elective repeat caesarean section versus planned vaginal birth for women with a previous caesarean birth. Cochrane Database Syst Rev.

[46] Shorten A, Fagerlin A, Illuzzi J (2015). Developing an internet-based decision aid for women choosing between vaginal birth after cesarean and planned repeat cesarean. J Midwif Womens Health.

[47] Althabe F, Belizán JM, Villar J (2004). Mandatory second opinion to reduce rates of unnecessary caesarean sections in Latin America: a cluster randomised controlled trial. Lancet.

[48] Chaillet N, Dumont A, Abrahamowicz M (2015). A cluster-randomized trial to reduce cesarean delivery rates in Quebec. New Engl J Med.

[49] Keeler EB, Fok T. (1996). Equalizing physician fees had little effect on cesarean rates. Med Care Res Rev.

