# Supplementary material for: Flat trend of high caesarean section rates in Peru: A pooled analysis of 3,376,062 births from the national birth registry, 2012 to 2020
Source: Lancet Reg Health Am. 2022 Jun 17;12:100293. doi: 10.1016/j.lana.2022.100293 (PMC9378316; doi:10.1016/j.lana.2022.100293)
Supplement: Supplementary file 1 [file mmc1.pdf]

Content

1. Supplementary Table 1. Caesarean section rates by region, province and year.

2. Supplementary Figure 1. Flow chart of the participants included in the analysis

3. Supplementary Figure 2. Caesarean section rates by region and year.

4. Supplementary Figure 3. Geographic and temporal profiles of caesarean section rates in Peru between 2012 and 2020.

5. Strengthening the Reporting of Observational studies in Epidemiology (STROBE) checklist.

Supplementary Table 1. C-section rate region, natural region, province and year.

Year	Natural region	Natural region C-section rate (%, 95% CI)	Region	Region C-section rate (%, 95% CI)	Province	Number of births	Province C-section rate (%, 95% CI)
2012	Amazon	22 (20 - 25)	AMAZONAS	22 (19 - 26)	BAGUA	181	43 (35 - 50)
2012	Amazon	22 (20 - 25)	AMAZONAS	22 (19 - 26)	CHACHAPOYAS	227	15 (11 - 20)
2012	Amazon	22 (20 - 25)	AMAZONAS	22 (19 - 26)	LUYA	45	0
2012	Amazon	22 (20 - 25)	SAN MARTIN	57 (47 - 67)	SAN MARTIN	101	57 (47 - 67)
2012	Coast	41 (40 - 41)	CALLAO	30 (29 - 31)	CALLAO	6941	30 (29 - 31)
2012	Coast	41 (40 - 41)	ICA	32 (27 - 37)	CHINCHA	129	43 (34 - 52)
2012	Coast	41 (40 - 41)	ICA	32 (27 - 37)	ICA	211	26 (20 - 32)
2012	Coast	41 (40 - 41)	LA LIBERTAD	48 (43 - 52)	TRUJILLO	523	48 (43 - 52)
2012	Coast	41 (40 - 41)	LIMA	42 (42 - 42)	HUAURA	1681	37 (34 - 39)
2012	Coast	41 (40 - 41)	LIMA	42 (42 - 42)	LIMA	48915	42 (42 - 43)
2012	Coast	41 (40 - 41)	PIURA	47 (45 - 49)	AYABACA	132	5 (2 - 10)
2012	Coast	41 (40 - 41)	PIURA	47 (45 - 49)	PIURA	2607	50 (48 - 52)
2012	Coast	41 (40 - 41)	PIURA	47 (45 - 49)	SULLANA	181	39 (32 - 46)
2012	Coast	41 (40 - 41)	TACNA	44 (39 - 48)	TACNA	448	44 (39 - 48)
2012	Highlands	35 (34 - 36)	APURIMAC	28 (26 - 32)	ABANCAY	740	28 (25 - 31)
2012	Highlands	35 (34 - 36)	APURIMAC	28 (26 - 32)	ANDAHUAYLAS	149	32 (24 - 40)
2012	Highlands	35 (34 - 36)	AREQUIPA	48 (45 - 51)	AREQUIPA	941	48 (45 - 51)
2012	Highlands	35 (34 - 36)	CAJAMARCA	39 (36 - 42)	CAJAMARCA	1151	39 (36 - 42)
2012	Highlands	35 (34 - 36)	CUSCO	33 (31 - 34)	CANCHIS	622	32 (28 - 35)
2012	Highlands	35 (34 - 36)	CUSCO	33 (31 - 34)	CUSCO	3824	33 (31 - 34)
2012	Highlands	35 (34 - 36)	HUANUCO	33 (31 - 35)	HUANUCO	909	38 (34 - 41)
2012	Highlands	35 (34 - 36)	HUANUCO	33 (31 - 35)	LEONCIO PRADO	611	26 (23 - 30)
2012	Highlands	35 (34 - 36)	PUNO	33 (27 - 39)	PUNO	67	33 (22 - 45)
2012	Highlands	35 (34 - 36)	PUNO	33 (27 - 39)	SAN ROMAN	161	33 (26 - 41)
2013	Amazon	33 (32 - 34)	AMAZONAS	32 (30 - 34)	BAGUA	774	47 (43 - 50)
2013	Amazon	33 (32 - 34)	AMAZONAS	32 (30 - 34)	BONGARA	77	0
2013	Amazon	33 (32 - 34)	AMAZONAS	32 (30 - 34)	CHACHAPOYAS	1203	30 (27 - 32)

2013	Amazon	33 (32 - 34)	AMAZONAS	32 (30 - 34)	CONDORCANQUI	33	48 (31 - 66)
2013	Amazon	33 (32 - 34)	AMAZONAS	32 (30 - 34)	LUYA	106	0
2013	Amazon	33 (32 - 34)	AMAZONAS	32 (30 - 34)	RODRIGUEZ DE MENDOZA	124	0
2013	Amazon	33 (32 - 34)	AMAZONAS	32 (30 - 34)	UTCUBAMBA	581	33 (29 - 37)
2013	Amazon	33 (32 - 34)	MADRE DE DIOS	22 (20 - 25)	TAMBOPATA	1080	22 (20 - 25)
2013	Amazon	33 (32 - 34)	SAN MARTIN	46 (44 - 48)	SAN MARTIN	2535	46 (44 - 48)
2013	Amazon	33 (32 - 34)	UCAYALI	19 (17 - 21)	CORONEL PORTILLO	756	29 (26 - 33)
2013	Amazon	33 (32 - 34)	UCAYALI	19 (17 - 21)	PADRE ABAD	538	4 (2 - 6)
2013	Coast	39 (39 - 39)	CALLAO	34 (33 - 34)	CALLAO	10239	34 (33 - 34)
2013	Coast	39 (39 - 39)	ICA	33 (32 - 35)	CHINCHA	2348	41 (39 - 43)
2013	Coast	39 (39 - 39)	ICA	33 (32 - 35)	ICA	2738	27 (25 - 29)
2013	Coast	39 (39 - 39)	LA LIBERTAD	40 (39 - 41)	ASCOPE	165	0
2013	Coast	39 (39 - 39)	LA LIBERTAD	40 (39 - 41)	CHEPEN	410	52 (47 - 57)
2013	Coast	39 (39 - 39)	LA LIBERTAD	40 (39 - 41)	GRAN CHIMU	46	0
2013	Coast	39 (39 - 39)	LA LIBERTAD	40 (39 - 41)	OTUZCO	162	48 (40 - 56)
2013	Coast	39 (39 - 39)	LA LIBERTAD	40 (39 - 41)	PACASMAYO	1065	41 (38 - 44)
2013	Coast	39 (39 - 39)	LA LIBERTAD	40 (39 - 41)	SANCHEZ CARRION	102	30 (22 - 40)
2013	Coast	39 (39 - 39)	LA LIBERTAD	40 (39 - 41)	TRUJILLO	8572	41 (40 - 42)
2013	Coast	39 (39 - 39)	LA LIBERTAD	40 (39 - 41)	VIRU	258	0
2013	Coast	39 (39 - 39)	LAMBAYEQUE	32 (30 - 33)	CHICLAYO	1657	43 (41 - 45)
2013	Coast	39 (39 - 39)	LAMBAYEQUE	32 (30 - 33)	FERREÑAFE	629	6 (4 - 8)
2013	Coast	39 (39 - 39)	LAMBAYEQUE	32 (30 - 33)	LAMBAYEQUE	1536	30 (28 - 32)
2013	Coast	39 (39 - 39)	LIMA	39 (39 - 39)	CAÑETE	1644	35 (33 - 37)
2013	Coast	39 (39 - 39)	LIMA	39 (39 - 39)	HUARAL	801	26 (23 - 30)
2013	Coast	39 (39 - 39)	LIMA	39 (39 - 39)	HUAURA	2962	36 (34 - 38)
2013	Coast	39 (39 - 39)	LIMA	39 (39 - 39)	LIMA	93763	39 (39 - 40)
2013	Coast	39 (39 - 39)	MOQUEGUA	40 (37 - 42)	ILO	667	42 (38 - 45)
2013	Coast	39 (39 - 39)	MOQUEGUA	40 (37 - 42)	MARISCAL NIETO	696	38 (34 - 42)
2013	Coast	39 (39 - 39)	PIURA	45 (44 - 46)	AYABACA	117	0
2013	Coast	39 (39 - 39)	PIURA	45 (44 - 46)	MORROPON	911	30 (27 - 33)
2013	Coast	39 (39 - 39)	PIURA	45 (44 - 46)	PIURA	3876	51 (49 - 53)

2013	Coast	39 (39 - 39)	PIURA	45 (44 - 46)	SECHURA	207	0
2013	Coast	39 (39 - 39)	PIURA	45 (44 - 46)	SULLANA	4097	47 (46 - 49)
2013	Coast	39 (39 - 39)	PIURA	45 (44 - 46)	TALARA	114	0
2013	Coast	39 (39 - 39)	TACNA	46 (44 - 47)	TACNA	3490	46 (44 - 47)
2013	Coast	39 (39 - 39)	TUMBES	49 (47 - 50)	CONTRALMIRANTE VILLAR	94	2 (0 - 7)
2013	Coast	39 (39 - 39)	TUMBES	49 (47 - 50)	TUMBES	2993	55 (53 - 57)
2013	Coast	39 (39 - 39)	TUMBES	49 (47 - 50)	ZARUMILLA	275	0
2013	Highlands	32 (32 - 33)	ANCASH	49 (44 - 53)	HUAYLAS	136	26 (19 - 35)
2013	Highlands	32 (32 - 33)	ANCASH	49 (44 - 53)	SANTA	412	56 (51 - 61)
2013	Highlands	32 (32 - 33)	APURIMAC	37 (35 - 39)	ABANCAY	1915	35 (33 - 38)
2013	Highlands	32 (32 - 33)	APURIMAC	37 (35 - 39)	ANDAHUAYLAS	1065	40 (37 - 43)
2013	Highlands	32 (32 - 33)	AREQUIPA	38 (37 - 39)	AREQUIPA	12503	40 (39 - 41)
2013	Highlands	32 (32 - 33)	AREQUIPA	38 (37 - 39)	CAMANA	1017	32 (29 - 35)
2013	Highlands	32 (32 - 33)	AREQUIPA	38 (37 - 39)	CASTILLA	53	45 (32 - 60)
2013	Highlands	32 (32 - 33)	AREQUIPA	38 (37 - 39)	CAYLLOMA	290	9 (6 - 12)
2013	Highlands	32 (32 - 33)	AREQUIPA	38 (37 - 39)	ISLAY	42	0
2013	Highlands	32 (32 - 33)	AYACUCHO	27 (26 - 28)	CANGALLO	261	26 (21 - 32)
2013	Highlands	32 (32 - 33)	AYACUCHO	27 (26 - 28)	HUAMANGA	3959	31 (29 - 32)
2013	Highlands	32 (32 - 33)	AYACUCHO	27 (26 - 28)	HUANCA SANCOS	61	0
2013	Highlands	32 (32 - 33)	AYACUCHO	27 (26 - 28)	HUANTA	941	26 (23 - 29)
2013	Highlands	32 (32 - 33)	AYACUCHO	27 (26 - 28)	LA MAR	576	26 (23 - 30)
2013	Highlands	32 (32 - 33)	AYACUCHO	27 (26 - 28)	LUCANAS	371	13 (10 - 17)
2013	Highlands	32 (32 - 33)	AYACUCHO	27 (26 - 28)	PARINACOCHAS	372	27 (22 - 31)
2013	Highlands	32 (32 - 33)	AYACUCHO	27 (26 - 28)	SUCRE	63	0
2013	Highlands	32 (32 - 33)	AYACUCHO	27 (26 - 28)	VICTOR FAJARDO	43	0
2013	Highlands	32 (32 - 33)	AYACUCHO	27 (26 - 28)	VILCAS HUAMAN	149	0
2013	Highlands	32 (32 - 33)	CAJAMARCA	28 (27 - 30)	CAJABAMBA	444	18 (15 - 22)
2013	Highlands	32 (32 - 33)	CAJAMARCA	28 (27 - 30)	CAJAMARCA	3932	29 (28 - 30)
2013	Highlands	32 (32 - 33)	CAJAMARCA	28 (27 - 30)	CHOTA	390	28 (24 - 33)
2013	Highlands	32 (32 - 33)	CAJAMARCA	28 (27 - 30)	JAEN	806	44 (41 - 48)
2013	Highlands	32 (32 - 33)	CAJAMARCA	28 (27 - 30)	SAN IGNACIO	117	0

2013	Highlands	32 (32 - 33)	CAJAMARCA	28 (27 - 30)	SAN MARCOS	233	0
2013	Highlands	32 (32 - 33)	CUSCO	33 (32 - 34)	ANTA	79	0
2013	Highlands	32 (32 - 33)	CUSCO	33 (32 - 34)	CALCA	180	0
2013	Highlands	32 (32 - 33)	CUSCO	33 (32 - 34)	CANCHIS	1335	25 (23 - 28)
2013	Highlands	32 (32 - 33)	CUSCO	33 (32 - 34)	CUSCO	9280	36 (35 - 37)
2013	Highlands	32 (32 - 33)	CUSCO	33 (32 - 34)	ESPINAR	533	16 (13 - 20)
2013	Highlands	32 (32 - 33)	CUSCO	33 (32 - 34)	LA CONVENCION	952	33 (30 - 36)
2013	Highlands	32 (32 - 33)	CUSCO	33 (32 - 34)	URUBAMBA	100	0
2013	Highlands	32 (32 - 33)	HUANCAVELICA	23 (21 - 24)	ACOBAMBA	472	7 (5 - 10)
2013	Highlands	32 (32 - 33)	HUANCAVELICA	23 (21 - 24)	ANGARAES	330	13 (10 - 17)
2013	Highlands	32 (32 - 33)	HUANCAVELICA	23 (21 - 24)	HUANCAVELICA	1135	28 (26 - 31)
2013	Highlands	32 (32 - 33)	HUANCAVELICA	23 (21 - 24)	TAYACAJA	664	29 (26 - 33)
2013	Highlands	32 (32 - 33)	HUANUCO	29 (28 - 30)	HUANUCO	3539	33 (32 - 35)
2013	Highlands	32 (32 - 33)	HUANUCO	29 (28 - 30)	LEONCIO PRADO	2413	25 (23 - 26)
2013	Highlands	32 (32 - 33)	HUANUCO	29 (28 - 30)	PACHITEA	223	0
2013	Highlands	32 (32 - 33)	JUNIN	33 (31 - 35)	CHANCHAMAYO	793	36 (33 - 40)
2013	Highlands	32 (32 - 33)	JUNIN	33 (31 - 35)	HUANCAYO	1548	33 (31 - 36)
2013	Highlands	32 (32 - 33)	JUNIN	33 (31 - 35)	JAUJA	399	26 (21 - 30)
2013	Highlands	32 (32 - 33)	PUNO	31 (30 - 33)	PUNO	2102	25 (24 - 27)
2013	Highlands	32 (32 - 33)	PUNO	31 (30 - 33)	SAN ROMAN	2733	36 (34 - 38)
2014	Amazon	28 (28 - 29)	AMAZONAS	27 (25 - 28)	BAGUA	851	43 (39 - 46)
2014	Amazon	28 (28 - 29)	AMAZONAS	27 (25 - 28)	BONGARA	143	0
2014	Amazon	28 (28 - 29)	AMAZONAS	27 (25 - 28)	CHACHAPOYAS	1414	24 (22 - 27)
2014	Amazon	28 (28 - 29)	AMAZONAS	27 (25 - 28)	CONDORCANQUI	113	15 (9 - 23)
2014	Amazon	28 (28 - 29)	AMAZONAS	27 (25 - 28)	LUYA	135	0
2014	Amazon	28 (28 - 29)	AMAZONAS	27 (25 - 28)	RODRIGUEZ DE MENDOZA	161	0
2014	Amazon	28 (28 - 29)	AMAZONAS	27 (25 - 28)	UTCUBAMBA	1039	29 (27 - 32)
2014	Amazon	28 (28 - 29)	LORETO	23 (22 - 25)	ALTO AMAZONAS	725	27 (24 - 31)
2014	Amazon	28 (28 - 29)	LORETO	23 (22 - 25)	DATEM DEL MARAÑON	132	1 (0 - 4)
2014	Amazon	28 (28 - 29)	LORETO	23 (22 - 25)	LORETO	177	1 (0 - 3)
2014	Amazon	28 (28 - 29)	LORETO	23 (22 - 25)	MARISCAL RAMON CASTILLA	86	0

2014	Amazon	28 (28 - 29)	LORETO	23 (22 - 25)	MAYNAS	2893	26 (24 - 27)
2014	Amazon	28 (28 - 29)	MADRE DE DIOS	24 (23 - 26)	TAHUAMANU	100	19 (12 - 28)
2014	Amazon	28 (28 - 29)	MADRE DE DIOS	24 (23 - 26)	TAMBOPATA	2050	25 (23 - 27)
2014	Amazon	28 (28 - 29)	SAN MARTIN	53 (51 - 55)	MOYOBAMBA	187	43 (36 - 51)
2014	Amazon	28 (28 - 29)	SAN MARTIN	53 (51 - 55)	SAN MARTIN	2946	54 (52 - 56)
2014	Amazon	28 (28 - 29)	UCAYALI	23 (22 - 24)	ATALAYA	242	11 (7 - 15)
2014	Amazon	28 (28 - 29)	UCAYALI	23 (22 - 24)	CORONEL PORTILLO	6563	25 (24 - 26)
2014	Amazon	28 (28 - 29)	UCAYALI	23 (22 - 24)	PADRE ABAD	662	4 (2 - 5)
2014	Coast	38 (38 - 38)	CALLAO	34 (33 - 35)	CALLAO	11595	34 (33 - 35)
2014	Coast	38 (38 - 38)	ICA	35 (34 - 36)	CHINCHA	2891	39 (38 - 41)
2014	Coast	38 (38 - 38)	ICA	35 (34 - 36)	ICA	4001	33 (31 - 34)
2014	Coast	38 (38 - 38)	ICA	35 (34 - 36)	PISCO	728	30 (27 - 34)
2014	Coast	38 (38 - 38)	LA LIBERTAD	36 (35 - 37)	ASCOPE	554	28 (25 - 32)
2014	Coast	38 (38 - 38)	LA LIBERTAD	36 (35 - 37)	CHEPEN	1189	48 (45 - 51)
2014	Coast	38 (38 - 38)	LA LIBERTAD	36 (35 - 37)	GRAN CHIMU	94	0
2014	Coast	38 (38 - 38)	LA LIBERTAD	36 (35 - 37)	OTUZCO	340	35 (30 - 40)
2014	Coast	38 (38 - 38)	LA LIBERTAD	36 (35 - 37)	PACASMAYO	1161	43 (40 - 46)
2014	Coast	38 (38 - 38)	LA LIBERTAD	36 (35 - 37)	PATAZ	82	0
2014	Coast	38 (38 - 38)	LA LIBERTAD	36 (35 - 37)	SANCHEZ CARRION	163	1 (0 - 3)
2014	Coast	38 (38 - 38)	LA LIBERTAD	36 (35 - 37)	SANTIAGO DE CHUCO	127	31 (24 - 40)
2014	Coast	38 (38 - 38)	LA LIBERTAD	36 (35 - 37)	TRUJILLO	10774	37 (36 - 38)
2014	Coast	38 (38 - 38)	LA LIBERTAD	36 (35 - 37)	VIRU	473	0
2014	Coast	38 (38 - 38)	LAMBAYEQUE	16 (15 - 16)	CHICLAYO	3037	14 (13 - 16)
2014	Coast	38 (38 - 38)	LAMBAYEQUE	16 (15 - 16)	FERREÑAFE	686	3 (2 - 5)
2014	Coast	38 (38 - 38)	LAMBAYEQUE	16 (15 - 16)	LAMBAYEQUE	3201	19 (18 - 21)
2014	Coast	38 (38 - 38)	LIMA	39 (39 - 40)	CAÑETE	2508	39 (37 - 41)
2014	Coast	38 (38 - 38)	LIMA	39 (39 - 40)	HUARAL	1943	25 (23 - 27)
2014	Coast	38 (38 - 38)	LIMA	39 (39 - 40)	HUAROCHIRI	130	13 (8 - 20)
2014	Coast	38 (38 - 38)	LIMA	39 (39 - 40)	HUAURA	2981	36 (35 - 38)
2014	Coast	38 (38 - 38)	LIMA	39 (39 - 40)	LIMA	108248	40 (40 - 40)
2014	Coast	38 (38 - 38)	MOQUEGUA	41 (39 - 43)	ILO	1173	42 (39 - 45)

2014	Coast	38 (38 - 38)	MOQUEGUA	41 (39 - 43)	MARISCAL NIETO	1267	39 (37 - 42)
2014	Coast	38 (38 - 38)	PIURA	40 (39 - 40)	AYABACA	178	0
2014	Coast	38 (38 - 38)	PIURA	40 (39 - 40)	HUANCABAMBA	38	0
2014	Coast	38 (38 - 38)	PIURA	40 (39 - 40)	MORROPON	1702	32 (30 - 34)
2014	Coast	38 (38 - 38)	PIURA	40 (39 - 40)	PAITA	948	17 (15 - 20)
2014	Coast	38 (38 - 38)	PIURA	40 (39 - 40)	PIURA	7755	48 (47 - 49)
2014	Coast	38 (38 - 38)	PIURA	40 (39 - 40)	SECHURA	352	0
2014	Coast	38 (38 - 38)	PIURA	40 (39 - 40)	SULLANA	4402	42 (40 - 43)
2014	Coast	38 (38 - 38)	PIURA	40 (39 - 40)	TALARA	705	16 (13 - 19)
2014	Coast	38 (38 - 38)	TACNA	45 (43 - 46)	TACNA	4242	45 (43 - 46)
2014	Coast	38 (38 - 38)	TUMBES	46 (44 - 47)	CONTRALMIRANTE VILLAR	107	0
2014	Coast	38 (38 - 38)	TUMBES	46 (44 - 47)	TUMBES	3498	51 (49 - 53)
2014	Coast	38 (38 - 38)	TUMBES	46 (44 - 47)	ZARUMILLA	275	0
2014	Highlands	32 (32 - 32)	ANCASH	46 (45 - 47)	HUARAZ	374	44 (39 - 49)
2014	Highlands	32 (32 - 32)	ANCASH	46 (45 - 47)	HUAYLAS	749	24 (21 - 27)
2014	Highlands	32 (32 - 32)	ANCASH	46 (45 - 47)	RECUAY	91	18 (10 - 27)
2014	Highlands	32 (32 - 32)	ANCASH	46 (45 - 47)	SANTA	3592	52 (50 - 54)
2014	Highlands	32 (32 - 32)	APURIMAC	35 (33 - 36)	ABANCAY	2406	35 (34 - 37)
2014	Highlands	32 (32 - 32)	APURIMAC	35 (33 - 36)	ANDAHUAYLAS	2009	38 (36 - 40)
2014	Highlands	32 (32 - 32)	APURIMAC	35 (33 - 36)	COTABAMBAS	409	15 (11 - 18)
2014	Highlands	32 (32 - 32)	APURIMAC	35 (33 - 36)	GRAU	33	0
2014	Highlands	32 (32 - 32)	AREQUIPA	41 (40 - 42)	AREQUIPA	20045	44 (43 - 44)
2014	Highlands	32 (32 - 32)	AREQUIPA	41 (40 - 42)	CAMANA	957	33 (30 - 36)
2014	Highlands	32 (32 - 32)	AREQUIPA	41 (40 - 42)	CARAVELI	195	0
2014	Highlands	32 (32 - 32)	AREQUIPA	41 (40 - 42)	CASTILLA	456	25 (22 - 30)
2014	Highlands	32 (32 - 32)	AREQUIPA	41 (40 - 42)	CAYLLOMA	1149	20 (17 - 22)
2014	Highlands	32 (32 - 32)	AREQUIPA	41 (40 - 42)	ISLAY	262	17 (13 - 22)
2014	Highlands	32 (32 - 32)	AYACUCHO	26 (25 - 27)	CANGALLO	431	28 (24 - 33)
2014	Highlands	32 (32 - 32)	AYACUCHO	26 (25 - 27)	HUAMANGA	5348	32 (31 - 34)
2014	Highlands	32 (32 - 32)	AYACUCHO	26 (25 - 27)	HUANCA SANCOS	80	0
2014	Highlands	32 (32 - 32)	AYACUCHO	26 (25 - 27)	HUANTA	1368	20 (18 - 22)

2014	Highlands	32 (32 - 32)	AYACUCHO	26 (25 - 27)	LA MAR	1100	19 (16 - 21)
2014	Highlands	32 (32 - 32)	AYACUCHO	26 (25 - 27)	LUCANAS	455	15 (12 - 18)
2014	Highlands	32 (32 - 32)	AYACUCHO	26 (25 - 27)	PARINACOCHAS	455	13 (10 - 17)
2014	Highlands	32 (32 - 32)	AYACUCHO	26 (25 - 27)	SUCRE	55	0
2014	Highlands	32 (32 - 32)	AYACUCHO	26 (25 - 27)	VICTOR FAJARDO	66	0
2014	Highlands	32 (32 - 32)	AYACUCHO	26 (25 - 27)	VILCAS HUAMAN	154	0
2014	Highlands	32 (32 - 32)	CAJAMARCA	24 (23 - 25)	CAJABAMBA	815	15 (13 - 18)
2014	Highlands	32 (32 - 32)	CAJAMARCA	24 (23 - 25)	CAJAMARCA	4899	22 (21 - 24)
2014	Highlands	32 (32 - 32)	CAJAMARCA	24 (23 - 25)	CELENDIN	438	3 (2 - 6)
2014	Highlands	32 (32 - 32)	CAJAMARCA	24 (23 - 25)	CHOTA	1431	29 (27 - 31)
2014	Highlands	32 (32 - 32)	CAJAMARCA	24 (23 - 25)	CUTERVO	797	28 (25 - 31)
2014	Highlands	32 (32 - 32)	CAJAMARCA	24 (23 - 25)	HUALGAYOC	35	6 (1 - 19)
2014	Highlands	32 (32 - 32)	CAJAMARCA	24 (23 - 25)	JAEN	1807	44 (42 - 46)
2014	Highlands	32 (32 - 32)	CAJAMARCA	24 (23 - 25)	SAN IGNACIO	442	0
2014	Highlands	32 (32 - 32)	CAJAMARCA	24 (23 - 25)	SAN MARCOS	335	0
2014	Highlands	32 (32 - 32)	CAJAMARCA	24 (23 - 25)	SANTA CRUZ	61	7 (2 - 16)
2014	Highlands	32 (32 - 32)	CUSCO	31 (30 - 32)	ANTA	360	0
2014	Highlands	32 (32 - 32)	CUSCO	31 (30 - 32)	CALCA	622	0
2014	Highlands	32 (32 - 32)	CUSCO	31 (30 - 32)	CANCHIS	1459	29 (27 - 32)
2014	Highlands	32 (32 - 32)	CUSCO	31 (30 - 32)	CUSCO	11535	37 (36 - 38)
2014	Highlands	32 (32 - 32)	CUSCO	31 (30 - 32)	ESPINAR	690	10 (8 - 12)
2014	Highlands	32 (32 - 32)	CUSCO	31 (30 - 32)	LA CONVENCION	1438	27 (25 - 29)
2014	Highlands	32 (32 - 32)	CUSCO	31 (30 - 32)	URUBAMBA	414	0
2014	Highlands	32 (32 - 32)	HUANCAVELICA	21 (20 - 23)	ACOBAMBA	688	11 (8 - 13)
2014	Highlands	32 (32 - 32)	HUANCAVELICA	21 (20 - 23)	ANGARAES	532	15 (12 - 18)
2014	Highlands	32 (32 - 32)	HUANCAVELICA	21 (20 - 23)	CHURCAMPA	108	1 (0 - 5)
2014	Highlands	32 (32 - 32)	HUANCAVELICA	21 (20 - 23)	HUANCAVELICA	1962	28 (26 - 30)
2014	Highlands	32 (32 - 32)	HUANCAVELICA	21 (20 - 23)	TAYACAJA	961	23 (20 - 25)
2014	Highlands	32 (32 - 32)	HUANUCO	24 (23 - 25)	AMBO	316	0
2014	Highlands	32 (32 - 32)	HUANUCO	24 (23 - 25)	DOS DE MAYO	208	8 (5 - 13)
2014	Highlands	32 (32 - 32)	HUANUCO	24 (23 - 25)	HUACAYBAMBA	46	0

2014	Highlands	32 (32 - 32)	HUANUCO	24 (23 - 25)	HUAMALIES	266	9 (6 - 13)
2014	Highlands	32 (32 - 32)	HUANUCO	24 (23 - 25)	HUANUCO	5304	29 (28 - 31)
2014	Highlands	32 (32 - 32)	HUANUCO	24 (23 - 25)	LEONCIO PRADO	2668	25 (24 - 27)
2014	Highlands	32 (32 - 32)	HUANUCO	24 (23 - 25)	PACHITEA	513	0
2014	Highlands	32 (32 - 32)	HUANUCO	24 (23 - 25)	PUERTO INCA	52	0
2014	Highlands	32 (32 - 32)	HUANUCO	24 (23 - 25)	YAROWILCA	39	0
2014	Highlands	32 (32 - 32)	JUNIN	35 (34 - 36)	CHANCHAMAYO	2088	36 (34 - 38)
2014	Highlands	32 (32 - 32)	JUNIN	35 (34 - 36)	HUANCAYO	5453	37 (36 - 38)
2014	Highlands	32 (32 - 32)	JUNIN	35 (34 - 36)	JAUJA	1090	20 (18 - 23)
2014	Highlands	32 (32 - 32)	PASCO	29 (27 - 30)	OXAPAMPA	569	28 (25 - 32)
2014	Highlands	32 (32 - 32)	PASCO	29 (27 - 30)	PASCO	1632	29 (26 - 31)
2014	Highlands	32 (32 - 32)	PUNO	31 (30 - 32)	AZANGARO	36	3 (0 - 15)
2014	Highlands	32 (32 - 32)	PUNO	31 (30 - 32)	CARABAYA	171	8 (5 - 13)
2014	Highlands	32 (32 - 32)	PUNO	31 (30 - 32)	CHUCUITO	211	10 (7 - 15)
2014	Highlands	32 (32 - 32)	PUNO	31 (30 - 32)	EL COLLAO	452	15 (12 - 18)
2014	Highlands	32 (32 - 32)	PUNO	31 (30 - 32)	HUANCANE	179	8 (5 - 13)
2014	Highlands	32 (32 - 32)	PUNO	31 (30 - 32)	LAMPA	105	19 (12 - 28)
2014	Highlands	32 (32 - 32)	PUNO	31 (30 - 32)	MELGAR	388	34 (29 - 38)
2014	Highlands	32 (32 - 32)	PUNO	31 (30 - 32)	PUNO	2239	26 (24 - 28)
2014	Highlands	32 (32 - 32)	PUNO	31 (30 - 32)	SAN ROMAN	3575	41 (39 - 42)
2014	Highlands	32 (32 - 32)	PUNO	31 (30 - 32)	SANDIA	88	27 (18 - 38)
2014	Highlands	32 (32 - 32)	PUNO	31 (30 - 32)	YUNGUYO	153	7 (4 - 12)
2015	Amazon	28 (28 - 29)	AMAZONAS	28 (27 - 29)	BAGUA	1132	39 (37 - 42)
2015	Amazon	28 (28 - 29)	AMAZONAS	28 (27 - 29)	BONGARA	177	0
2015	Amazon	28 (28 - 29)	AMAZONAS	28 (27 - 29)	CHACHAPOYAS	1481	29 (27 - 32)
2015	Amazon	28 (28 - 29)	AMAZONAS	28 (27 - 29)	CONDORCANQUI	186	10 (6 - 15)
2015	Amazon	28 (28 - 29)	AMAZONAS	28 (27 - 29)	LUYA	146	0
2015	Amazon	28 (28 - 29)	AMAZONAS	28 (27 - 29)	RODRIGUEZ DE MENDOZA	157	0
2015	Amazon	28 (28 - 29)	AMAZONAS	28 (27 - 29)	UTCUBAMBA	1256	29 (27 - 32)
2015	Amazon	28 (28 - 29)	LORETO	25 (24 - 25)	ALTO AMAZONAS	2231	25 (23 - 27)
2015	Amazon	28 (28 - 29)	LORETO	25 (24 - 25)	DATEM DEL MARAÑON	253	0

2015	Amazon	28 (28 - 29)	LORETO	25 (24 - 25)	LORETO	447	1 (0 - 2)
2015	Amazon	28 (28 - 29)	LORETO	25 (24 - 25)	MARISCAL RAMON CASTILLA	265	0
2015	Amazon	28 (28 - 29)	LORETO	25 (24 - 25)	MAYNAS	8485	27 (26 - 28)
2015	Amazon	28 (28 - 29)	LORETO	25 (24 - 25)	UCAYALI	41	0
2015	Amazon	28 (28 - 29)	MADRE DE DIOS	30 (28 - 31)	TAHUAMANU	137	23 (16 - 31)
2015	Amazon	28 (28 - 29)	MADRE DE DIOS	30 (28 - 31)	TAMBOPATA	2668	30 (28 - 32)
2015	Amazon	28 (28 - 29)	SAN MARTIN	36 (35 - 37)	BELLAVISTA	368	0
2015	Amazon	28 (28 - 29)	SAN MARTIN	36 (35 - 37)	EL DORADO	419	0
2015	Amazon	28 (28 - 29)	SAN MARTIN	36 (35 - 37)	LAMAS	316	0
2015	Amazon	28 (28 - 29)	SAN MARTIN	36 (35 - 37)	MARISCAL CACERES	790	30 (27 - 33)
2015	Amazon	28 (28 - 29)	SAN MARTIN	36 (35 - 37)	MOYOBAMBA	2056	32 (30 - 34)
2015	Amazon	28 (28 - 29)	SAN MARTIN	36 (35 - 37)	RIOJA	1759	31 (29 - 33)
2015	Amazon	28 (28 - 29)	SAN MARTIN	36 (35 - 37)	SAN MARTIN	4258	51 (50 - 53)
2015	Amazon	28 (28 - 29)	SAN MARTIN	36 (35 - 37)	TOCACHE	838	30 (27 - 34)
2015	Amazon	28 (28 - 29)	UCAYALI	25 (24 - 26)	ATALAYA	533	8 (5 - 10)
2015	Amazon	28 (28 - 29)	UCAYALI	25 (24 - 26)	CORONEL PORTILLO	8924	27 (27 - 28)
2015	Amazon	28 (28 - 29)	UCAYALI	25 (24 - 26)	PADRE ABAD	556	1 (1 - 3)
2015	Coast	40 (40 - 40)	CALLAO	36 (35 - 37)	CALLAO	16863	36 (35 - 37)
2015	Coast	40 (40 - 40)	ICA	39 (38 - 40)	CHINCHA	3890	37 (35 - 38)
2015	Coast	40 (40 - 40)	ICA	39 (38 - 40)	ICA	8149	42 (41 - 44)
2015	Coast	40 (40 - 40)	ICA	39 (38 - 40)	NAZCA	418	24 (20 - 28)
2015	Coast	40 (40 - 40)	ICA	39 (38 - 40)	PISCO	2870	36 (34 - 38)
2015	Coast	40 (40 - 40)	LA LIBERTAD	37 (36 - 38)	ASCOPE	1270	29 (27 - 32)
2015	Coast	40 (40 - 40)	LA LIBERTAD	37 (36 - 38)	CHEPEN	1183	43 (40 - 46)
2015	Coast	40 (40 - 40)	LA LIBERTAD	37 (36 - 38)	GRAN CHIMU	70	0
2015	Coast	40 (40 - 40)	LA LIBERTAD	37 (36 - 38)	JULCAN	121	0
2015	Coast	40 (40 - 40)	LA LIBERTAD	37 (36 - 38)	OTUZCO	483	33 (28 - 37)
2015	Coast	40 (40 - 40)	LA LIBERTAD	37 (36 - 38)	PACASMAYO	1155	43 (40 - 46)
2015	Coast	40 (40 - 40)	LA LIBERTAD	37 (36 - 38)	PATAZ	184	5 (3 - 10)
2015	Coast	40 (40 - 40)	LA LIBERTAD	37 (36 - 38)	SANCHEZ CARRION	1112	26 (23 - 28)
2015	Coast	40 (40 - 40)	LA LIBERTAD	37 (36 - 38)	SANTIAGO DE CHUCO	593	25 (21 - 28)

2015	Coast	40 (40 - 40)	LA LIBERTAD	37 (36 - 38)	TRUJILLO	15325	40 (40 - 41)
2015	Coast	40 (40 - 40)	LA LIBERTAD	37 (36 - 38)	VIRU	542	0
2015	Coast	40 (40 - 40)	LAMBAYEQUE	28 (27 - 29)	CHICLAYO	9384	33 (32 - 34)
2015	Coast	40 (40 - 40)	LAMBAYEQUE	28 (27 - 29)	FERREÑAFE	740	6 (5 - 8)
2015	Coast	40 (40 - 40)	LAMBAYEQUE	28 (27 - 29)	LAMBAYEQUE	3896	20 (19 - 21)
2015	Coast	40 (40 - 40)	LIMA	42 (42 - 42)	BARRANCA	1363	41 (38 - 44)
2015	Coast	40 (40 - 40)	LIMA	42 (42 - 42)	CAÑETE	2997	37 (35 - 38)
2015	Coast	40 (40 - 40)	LIMA	42 (42 - 42)	HUARAL	2821	24 (22 - 26)
2015	Coast	40 (40 - 40)	LIMA	42 (42 - 42)	HUAROCHIRI	171	8 (5 - 13)
2015	Coast	40 (40 - 40)	LIMA	42 (42 - 42)	HUAURA	2967	38 (37 - 40)
2015	Coast	40 (40 - 40)	LIMA	42 (42 - 42)	LIMA	131510	43 (42 - 43)
2015	Coast	40 (40 - 40)	MOQUEGUA	40 (38 - 42)	GENERAL SANCHEZ CERRO	35	0
2015	Coast	40 (40 - 40)	MOQUEGUA	40 (38 - 42)	ILO	1187	43 (40 - 45)
2015	Coast	40 (40 - 40)	MOQUEGUA	40 (38 - 42)	MARISCAL NIETO	1349	39 (37 - 42)
2015	Coast	40 (40 - 40)	PIURA	36 (36 - 37)	AYABACA	442	0
2015	Coast	40 (40 - 40)	PIURA	36 (36 - 37)	HUANCABAMBA	490	4 (3 - 6)
2015	Coast	40 (40 - 40)	PIURA	36 (36 - 37)	MORROPON	2051	28 (26 - 30)
2015	Coast	40 (40 - 40)	PIURA	36 (36 - 37)	PAITA	1381	16 (14 - 18)
2015	Coast	40 (40 - 40)	PIURA	36 (36 - 37)	PIURA	9660	43 (42 - 44)
2015	Coast	40 (40 - 40)	PIURA	36 (36 - 37)	SECHURA	597	7 (5 - 9)
2015	Coast	40 (40 - 40)	PIURA	36 (36 - 37)	SULLANA	4307	46 (44 - 47)
2015	Coast	40 (40 - 40)	PIURA	36 (36 - 37)	TALARA	927	21 (19 - 24)
2015	Coast	40 (40 - 40)	TACNA	47 (45 - 48)	TACNA	5080	47 (45 - 48)
2015	Coast	40 (40 - 40)	TUMBES	51 (49 - 52)	CONTRALMIRANTE VILLAR	121	0
2015	Coast	40 (40 - 40)	TUMBES	51 (49 - 52)	TUMBES	3844	56 (54 - 57)
2015	Coast	40 (40 - 40)	TUMBES	51 (49 - 52)	ZARUMILLA	264	0
2015	Highlands	30 (30 - 30)	ANCASH	34 (33 - 35)	CARHUAZ	528	1 (0 - 2)
2015	Highlands	30 (30 - 30)	ANCASH	34 (33 - 35)	HUARAZ	3713	33 (32 - 35)
2015	Highlands	30 (30 - 30)	ANCASH	34 (33 - 35)	HUARI	542	23 (19 - 26)
2015	Highlands	30 (30 - 30)	ANCASH	34 (33 - 35)	HUAYLAS	799	27 (24 - 30)
2015	Highlands	30 (30 - 30)	ANCASH	34 (33 - 35)	POMABAMBA	305	18 (14 - 22)

2015	Highlands	30 (30 - 30)	ANCASH	34 (33 - 35)	RECUAY	139	14 (9 - 21)
2015	Highlands	30 (30 - 30)	ANCASH	34 (33 - 35)	SANTA	5659	44 (43 - 45)
2015	Highlands	30 (30 - 30)	ANCASH	34 (33 - 35)	SIHUAS	210	0
2015	Highlands	30 (30 - 30)	ANCASH	34 (33 - 35)	YUNGAY	368	0
2015	Highlands	30 (30 - 30)	APURIMAC	31 (30 - 32)	ABANCAY	2685	39 (37 - 41)
2015	Highlands	30 (30 - 30)	APURIMAC	31 (30 - 32)	ANDAHUAYLAS	2262	33 (31 - 35)
2015	Highlands	30 (30 - 30)	APURIMAC	31 (30 - 32)	ANTABAMBA	43	0
2015	Highlands	30 (30 - 30)	APURIMAC	31 (30 - 32)	AYMARAES	44	0
2015	Highlands	30 (30 - 30)	APURIMAC	31 (30 - 32)	CHINCHEROS	208	5 (2 - 9)
2015	Highlands	30 (30 - 30)	APURIMAC	31 (30 - 32)	COTABAMBAS	701	10 (8 - 13)
2015	Highlands	30 (30 - 30)	APURIMAC	31 (30 - 32)	GRAU	133	0
2015	Highlands	30 (30 - 30)	AREQUIPA	42 (42 - 43)	AREQUIPA	20571	45 (45 - 46)
2015	Highlands	30 (30 - 30)	AREQUIPA	42 (42 - 43)	CAMANA	981	35 (32 - 38)
2015	Highlands	30 (30 - 30)	AREQUIPA	42 (42 - 43)	CARAVELI	190	0
2015	Highlands	30 (30 - 30)	AREQUIPA	42 (42 - 43)	CASTILLA	405	28 (23 - 32)
2015	Highlands	30 (30 - 30)	AREQUIPA	42 (42 - 43)	CAYLLOMA	1194	19 (17 - 21)
2015	Highlands	30 (30 - 30)	AREQUIPA	42 (42 - 43)	CONDESUYOS	49	2 (0 - 11)
2015	Highlands	30 (30 - 30)	AREQUIPA	42 (42 - 43)	ISLAY	455	31 (26 - 35)
2015	Highlands	30 (30 - 30)	AYACUCHO	25 (24 - 26)	CANGALLO	464	20 (16 - 24)
2015	Highlands	30 (30 - 30)	AYACUCHO	25 (24 - 26)	HUAMANGA	6482	32 (31 - 33)
2015	Highlands	30 (30 - 30)	AYACUCHO	25 (24 - 26)	HUANCA SANCOS	81	0
2015	Highlands	30 (30 - 30)	AYACUCHO	25 (24 - 26)	HUANTA	1615	18 (16 - 20)
2015	Highlands	30 (30 - 30)	AYACUCHO	25 (24 - 26)	LA MAR	1483	14 (12 - 16)
2015	Highlands	30 (30 - 30)	AYACUCHO	25 (24 - 26)	LUCANAS	495	12 (10 - 16)
2015	Highlands	30 (30 - 30)	AYACUCHO	25 (24 - 26)	PARINACOCHAS	479	24 (20 - 28)
2015	Highlands	30 (30 - 30)	AYACUCHO	25 (24 - 26)	PAUCAR DEL SARA SARA	65	0
2015	Highlands	30 (30 - 30)	AYACUCHO	25 (24 - 26)	SUCRE	70	0
2015	Highlands	30 (30 - 30)	AYACUCHO	25 (24 - 26)	VICTOR FAJARDO	55	0
2015	Highlands	30 (30 - 30)	AYACUCHO	25 (24 - 26)	VILCAS HUAMAN	149	0
2015	Highlands	30 (30 - 30)	CAJAMARCA	23 (22 - 24)	CAJABAMBA	899	17 (15 - 20)
2015	Highlands	30 (30 - 30)	CAJAMARCA	23 (22 - 24)	CAJAMARCA	6220	22 (21 - 23)

2015	Highlands	30 (30 - 30)	CAJAMARCA	23 (22 - 24)	CELENDIN	506	1 (0 - 2)
2015	Highlands	30 (30 - 30)	CAJAMARCA	23 (22 - 24)	CHOTA	1556	31 (29 - 34)
2015	Highlands	30 (30 - 30)	CAJAMARCA	23 (22 - 24)	CONTUMAZA	55	0
2015	Highlands	30 (30 - 30)	CAJAMARCA	23 (22 - 24)	CUTERVO	961	29 (26 - 32)
2015	Highlands	30 (30 - 30)	CAJAMARCA	23 (22 - 24)	HUALGAYOC	615	12 (10 - 15)
2015	Highlands	30 (30 - 30)	CAJAMARCA	23 (22 - 24)	JAEN	2432	37 (35 - 39)
2015	Highlands	30 (30 - 30)	CAJAMARCA	23 (22 - 24)	SAN IGNACIO	595	0
2015	Highlands	30 (30 - 30)	CAJAMARCA	23 (22 - 24)	SAN MARCOS	309	0
2015	Highlands	30 (30 - 30)	CAJAMARCA	23 (22 - 24)	SANTA CRUZ	155	4 (1 - 8)
2015	Highlands	30 (30 - 30)	CUSCO	31 (30 - 32)	ANTA	328	0
2015	Highlands	30 (30 - 30)	CUSCO	31 (30 - 32)	CALCA	562	0
2015	Highlands	30 (30 - 30)	CUSCO	31 (30 - 32)	CANAS	120	0
2015	Highlands	30 (30 - 30)	CUSCO	31 (30 - 32)	CANCHIS	1898	22 (20 - 24)
2015	Highlands	30 (30 - 30)	CUSCO	31 (30 - 32)	CHUMBIVILCAS	82	17 (10 - 27)
2015	Highlands	30 (30 - 30)	CUSCO	31 (30 - 32)	CUSCO	12238	40 (39 - 41)
2015	Highlands	30 (30 - 30)	CUSCO	31 (30 - 32)	ESPINAR	722	6 (5 - 8)
2015	Highlands	30 (30 - 30)	CUSCO	31 (30 - 32)	LA CONVENCION	2329	24 (23 - 26)
2015	Highlands	30 (30 - 30)	CUSCO	31 (30 - 32)	QUISPICANCHI	447	0
2015	Highlands	30 (30 - 30)	CUSCO	31 (30 - 32)	URUBAMBA	381	0
2015	Highlands	30 (30 - 30)	HUANCAVELICA	19 (18 - 21)	ACOBAMBA	616	12 (9 - 15)
2015	Highlands	30 (30 - 30)	HUANCAVELICA	19 (18 - 21)	ANGARAES	647	15 (13 - 18)
2015	Highlands	30 (30 - 30)	HUANCAVELICA	19 (18 - 21)	CHURCAMPA	431	0
2015	Highlands	30 (30 - 30)	HUANCAVELICA	19 (18 - 21)	HUANCAVELICA	2335	27 (25 - 29)
2015	Highlands	30 (30 - 30)	HUANCAVELICA	19 (18 - 21)	HUAYTARA	69	0
2015	Highlands	30 (30 - 30)	HUANCAVELICA	19 (18 - 21)	TAYACAJA	907	19 (16 - 21)
2015	Highlands	30 (30 - 30)	HUANUCO	21 (21 - 22)	AMBO	452	0
2015	Highlands	30 (30 - 30)	HUANUCO	21 (21 - 22)	DOS DE MAYO	373	7 (5 - 10)
2015	Highlands	30 (30 - 30)	HUANUCO	21 (21 - 22)	HUACAYBAMBA	104	0
2015	Highlands	30 (30 - 30)	HUANUCO	21 (21 - 22)	HUAMALIES	632	8 (6 - 10)
2015	Highlands	30 (30 - 30)	HUANUCO	21 (21 - 22)	HUANUCO	6175	27 (26 - 28)
2015	Highlands	30 (30 - 30)	HUANUCO	21 (21 - 22)	LAURICOCHA	73	0

2015	Highlands	30 (30 - 30)	HUANUCO	21 (21 - 22)	LEONCIO PRADO	3166	28 (26 - 29)
2015	Highlands	30 (30 - 30)	HUANUCO	21 (21 - 22)	MARAÑON	69	0
2015	Highlands	30 (30 - 30)	HUANUCO	21 (21 - 22)	PACHITEA	853	0
2015	Highlands	30 (30 - 30)	HUANUCO	21 (21 - 22)	PUERTO INCA	195	0
2015	Highlands	30 (30 - 30)	HUANUCO	21 (21 - 22)	YAROWILCA	201	0
2015	Highlands	30 (30 - 30)	JUNIN	31 (30 - 31)	CHANCHAMAYO	2286	38 (36 - 40)
2015	Highlands	30 (30 - 30)	JUNIN	31 (30 - 31)	CHUPACA	260	0
2015	Highlands	30 (30 - 30)	JUNIN	31 (30 - 31)	CONCEPCION	150	0
2015	Highlands	30 (30 - 30)	JUNIN	31 (30 - 31)	HUANCAYO	7146	34 (33 - 35)
2015	Highlands	30 (30 - 30)	JUNIN	31 (30 - 31)	JAUJA	1034	22 (20 - 25)
2015	Highlands	30 (30 - 30)	JUNIN	31 (30 - 31)	JUNIN	188	8 (5 - 13)
2015	Highlands	30 (30 - 30)	JUNIN	31 (30 - 31)	SATIPO	901	26 (23 - 29)
2015	Highlands	30 (30 - 30)	JUNIN	31 (30 - 31)	TARMA	648	14 (12 - 17)
2015	Highlands	30 (30 - 30)	JUNIN	31 (30 - 31)	YAULI	174	20 (14 - 26)
2015	Highlands	30 (30 - 30)	PASCO	21 (20 - 22)	DANIEL ALCIDES CARRION	157	0
2015	Highlands	30 (30 - 30)	PASCO	21 (20 - 22)	OXAPAMPA	1502	18 (16 - 20)
2015	Highlands	30 (30 - 30)	PASCO	21 (20 - 22)	PASCO	2540	24 (22 - 26)
2015	Highlands	30 (30 - 30)	PUNO	26 (26 - 27)	AZANGARO	683	9 (7 - 12)
2015	Highlands	30 (30 - 30)	PUNO	26 (26 - 27)	CARABAYA	325	7 (5 - 11)
2015	Highlands	30 (30 - 30)	PUNO	26 (26 - 27)	CHUCUITO	616	9 (7 - 11)
2015	Highlands	30 (30 - 30)	PUNO	26 (26 - 27)	EL COLLAO	811	13 (10 - 15)
2015	Highlands	30 (30 - 30)	PUNO	26 (26 - 27)	HUANCANE	435	6 (4 - 9)
2015	Highlands	30 (30 - 30)	PUNO	26 (26 - 27)	LAMPA	215	11 (7 - 16)
2015	Highlands	30 (30 - 30)	PUNO	26 (26 - 27)	MELGAR	728	23 (20 - 26)
2015	Highlands	30 (30 - 30)	PUNO	26 (26 - 27)	MOHO	35	0
2015	Highlands	30 (30 - 30)	PUNO	26 (26 - 27)	PUNO	3020	30 (28 - 32)
2015	Highlands	30 (30 - 30)	PUNO	26 (26 - 27)	SAN ROMAN	4564	38 (37 - 40)
2015	Highlands	30 (30 - 30)	PUNO	26 (26 - 27)	SANDIA	190	13 (8 - 18)
2015	Highlands	30 (30 - 30)	PUNO	26 (26 - 27)	YUNGUYO	358	7 (5 - 10)
2016	Amazon	28 (27 - 28)	AMAZONAS	29 (27 - 30)	BAGUA	1135	46 (43 - 49)
2016	Amazon	28 (27 - 28)	AMAZONAS	29 (27 - 30)	BONGARA	212	0

2016	Amazon	28 (27 - 28)	AMAZONAS	29 (27 - 30)	CHACHAPOYAS	1613	33 (31 - 36)
2016	Amazon	28 (27 - 28)	AMAZONAS	29 (27 - 30)	CONDORCANQUI	400	9 (6 - 12)
2016	Amazon	28 (27 - 28)	AMAZONAS	29 (27 - 30)	LUYA	161	0
2016	Amazon	28 (27 - 28)	AMAZONAS	29 (27 - 30)	RODRIGUEZ DE MENDOZA	154	0
2016	Amazon	28 (27 - 28)	AMAZONAS	29 (27 - 30)	UTCUBAMBA	1306	25 (23 - 28)
2016	Amazon	28 (27 - 28)	LORETO	25 (24 - 26)	ALTO AMAZONAS	2470	23 (22 - 25)
2016	Amazon	28 (27 - 28)	LORETO	25 (24 - 26)	DATEM DEL MARAÑON	361	0
2016	Amazon	28 (27 - 28)	LORETO	25 (24 - 26)	LORETO	484	0
2016	Amazon	28 (27 - 28)	LORETO	25 (24 - 26)	MARISCAL RAMON CASTILLA	494	0
2016	Amazon	28 (27 - 28)	LORETO	25 (24 - 26)	MAYNAS	10250	31 (30 - 32)
2016	Amazon	28 (27 - 28)	LORETO	25 (24 - 26)	REQUENA	590	1 (0 - 1)
2016	Amazon	28 (27 - 28)	LORETO	25 (24 - 26)	UCAYALI	428	5 (3 - 8)
2016	Amazon	28 (27 - 28)	MADRE DE DIOS	27 (25 - 29)	MANU	37	0
2016	Amazon	28 (27 - 28)	MADRE DE DIOS	27 (25 - 29)	TAHUAMANU	110	12 (6 - 19)
2016	Amazon	28 (27 - 28)	MADRE DE DIOS	27 (25 - 29)	TAMBOPATA	2882	28 (26 - 30)
2016	Amazon	28 (27 - 28)	SAN MARTIN	30 (30 - 31)	BELLAVISTA	498	0
2016	Amazon	28 (27 - 28)	SAN MARTIN	30 (30 - 31)	EL DORADO	540	0
2016	Amazon	28 (27 - 28)	SAN MARTIN	30 (30 - 31)	HUALLAGA	201	0
2016	Amazon	28 (27 - 28)	SAN MARTIN	30 (30 - 31)	LAMAS	749	0
2016	Amazon	28 (27 - 28)	SAN MARTIN	30 (30 - 31)	MARISCAL CACERES	938	29 (27 - 32)
2016	Amazon	28 (27 - 28)	SAN MARTIN	30 (30 - 31)	MOYOBAMBA	2483	33 (31 - 35)
2016	Amazon	28 (27 - 28)	SAN MARTIN	30 (30 - 31)	PICOTA	332	0
2016	Amazon	28 (27 - 28)	SAN MARTIN	30 (30 - 31)	RIOJA	2244	23 (21 - 25)
2016	Amazon	28 (27 - 28)	SAN MARTIN	30 (30 - 31)	SAN MARTIN	4842	49 (48 - 51)
2016	Amazon	28 (27 - 28)	SAN MARTIN	30 (30 - 31)	TOCACHE	1186	23 (20 - 25)
2016	Amazon	28 (27 - 28)	UCAYALI	27 (27 - 28)	ATALAYA	598	5 (3 - 7)
2016	Amazon	28 (27 - 28)	UCAYALI	27 (27 - 28)	CORONEL PORTILLO	9751	31 (30 - 32)
2016	Amazon	28 (27 - 28)	UCAYALI	27 (27 - 28)	PADRE ABAD	812	2 (2 - 4)
2016	Coast	40 (39 - 40)	CALLAO	37 (36 - 37)	CALLAO	16309	37 (36 - 37)
2016	Coast	40 (39 - 40)	ICA	39 (38 - 39)	CHINCHA	3882	35 (33 - 36)
2016	Coast	40 (39 - 40)	ICA	39 (38 - 39)	ICA	8070	43 (42 - 44)

2016	Coast	40 (39 - 40)	ICA	39 (38 - 39)	NAZCA	945	29 (26 - 32)
2016	Coast	40 (39 - 40)	ICA	39 (38 - 39)	PALPA	122	0
2016	Coast	40 (39 - 40)	ICA	39 (38 - 39)	PISCO	2922	38 (36 - 39)
2016	Coast	40 (39 - 40)	LA LIBERTAD	36 (35 - 37)	ASCOPE	1245	25 (23 - 27)
2016	Coast	40 (39 - 40)	LA LIBERTAD	36 (35 - 37)	BOLIVAR	45	0
2016	Coast	40 (39 - 40)	LA LIBERTAD	36 (35 - 37)	CHEPEN	1310	45 (42 - 47)
2016	Coast	40 (39 - 40)	LA LIBERTAD	36 (35 - 37)	GRAN CHIMU	113	0
2016	Coast	40 (39 - 40)	LA LIBERTAD	36 (35 - 37)	JULCAN	217	0
2016	Coast	40 (39 - 40)	LA LIBERTAD	36 (35 - 37)	OTUZCO	581	33 (29 - 37)
2016	Coast	40 (39 - 40)	LA LIBERTAD	36 (35 - 37)	PACASMAYO	1150	39 (36 - 42)
2016	Coast	40 (39 - 40)	LA LIBERTAD	36 (35 - 37)	PATAZ	480	0
2016	Coast	40 (39 - 40)	LA LIBERTAD	36 (35 - 37)	SANCHEZ CARRION	1818	22 (20 - 24)
2016	Coast	40 (39 - 40)	LA LIBERTAD	36 (35 - 37)	SANTIAGO DE CHUCO	487	20 (17 - 24)
2016	Coast	40 (39 - 40)	LA LIBERTAD	36 (35 - 37)	TRUJILLO	15166	42 (42 - 43)
2016	Coast	40 (39 - 40)	LA LIBERTAD	36 (35 - 37)	VIRU	923	0
2016	Coast	40 (39 - 40)	LAMBAYEQUE	33 (32 - 33)	CHICLAYO	13161	38 (37 - 38)
2016	Coast	40 (39 - 40)	LAMBAYEQUE	33 (32 - 33)	FERREÑAFE	713	7 (5 - 9)
2016	Coast	40 (39 - 40)	LAMBAYEQUE	33 (32 - 33)	LAMBAYEQUE	4163	21 (20 - 22)
2016	Coast	40 (39 - 40)	LIMA	42 (42 - 43)	BARRANCA	2224	40 (38 - 42)
2016	Coast	40 (39 - 40)	LIMA	42 (42 - 43)	CAÑETE	4117	29 (28 - 31)
2016	Coast	40 (39 - 40)	LIMA	42 (42 - 43)	HUARAL	2667	26 (24 - 27)
2016	Coast	40 (39 - 40)	LIMA	42 (42 - 43)	HUAROCHIRI	241	5 (3 - 9)
2016	Coast	40 (39 - 40)	LIMA	42 (42 - 43)	HUAURA	3080	38 (36 - 39)
2016	Coast	40 (39 - 40)	LIMA	42 (42 - 43)	LIMA	138565	43 (43 - 43)
2016	Coast	40 (39 - 40)	MOQUEGUA	40 (38 - 42)	GENERAL SANCHEZ CERRO	42	0
2016	Coast	40 (39 - 40)	MOQUEGUA	40 (38 - 42)	ILO	1086	42 (39 - 45)
2016	Coast	40 (39 - 40)	MOQUEGUA	40 (38 - 42)	MARISCAL NIETO	1312	39 (36 - 42)
2016	Coast	40 (39 - 40)	PIURA	32 (31 - 32)	AYABACA	691	0
2016	Coast	40 (39 - 40)	PIURA	32 (31 - 32)	HUANCABAMBA	733	3 (2 - 4)
2016	Coast	40 (39 - 40)	PIURA	32 (31 - 32)	MORROPON	2294	32 (30 - 34)
2016	Coast	40 (39 - 40)	PIURA	32 (31 - 32)	PAITA	1598	21 (19 - 23)

2016	Coast	40 (39 - 40)	PIURA	32 (31 - 32)	PIURA	10804	35 (34 - 36)
2016	Coast	40 (39 - 40)	PIURA	32 (31 - 32)	SECHURA	899	8 (6 - 10)
2016	Coast	40 (39 - 40)	PIURA	32 (31 - 32)	SULLANA	4770	42 (41 - 44)
2016	Coast	40 (39 - 40)	PIURA	32 (31 - 32)	TALARA	753	18 (16 - 21)
2016	Coast	40 (39 - 40)	TACNA	45 (44 - 46)	TACNA	5018	45 (44 - 47)
2016	Coast	40 (39 - 40)	TUMBES	47 (46 - 49)	CONTRALMIRANTE VILLAR	111	0
2016	Coast	40 (39 - 40)	TUMBES	47 (46 - 49)	TUMBES	3792	52 (51 - 54)
2016	Coast	40 (39 - 40)	TUMBES	47 (46 - 49)	ZARUMILLA	290	0
2016	Highlands	28 (28 - 28)	ANCASH	33 (32 - 34)	ANTONIO RAIMONDI	58	0
2016	Highlands	28 (28 - 28)	ANCASH	33 (32 - 34)	ASUNCION	45	0
2016	Highlands	28 (28 - 28)	ANCASH	33 (32 - 34)	CARHUAZ	506	0
2016	Highlands	28 (28 - 28)	ANCASH	33 (32 - 34)	CARLOS FERMIN FITZCARRALD	120	0
2016	Highlands	28 (28 - 28)	ANCASH	33 (32 - 34)	HUARAZ	4194	35 (34 - 37)
2016	Highlands	28 (28 - 28)	ANCASH	33 (32 - 34)	HUARI	553	23 (20 - 27)
2016	Highlands	28 (28 - 28)	ANCASH	33 (32 - 34)	HUAYLAS	827	26 (23 - 29)
2016	Highlands	28 (28 - 28)	ANCASH	33 (32 - 34)	MARISCAL LUZURIAGA	74	0
2016	Highlands	28 (28 - 28)	ANCASH	33 (32 - 34)	POMABAMBA	379	20 (16 - 24)
2016	Highlands	28 (28 - 28)	ANCASH	33 (32 - 34)	RECUAY	168	20 (14 - 26)
2016	Highlands	28 (28 - 28)	ANCASH	33 (32 - 34)	SANTA	6427	43 (41 - 44)
2016	Highlands	28 (28 - 28)	ANCASH	33 (32 - 34)	SIHUAS	253	1 (0 - 3)
2016	Highlands	28 (28 - 28)	ANCASH	33 (32 - 34)	YUNGAY	473	0
2016	Highlands	28 (28 - 28)	APURIMAC	26 (25 - 27)	ABANCAY	2656	39 (37 - 41)
2016	Highlands	28 (28 - 28)	APURIMAC	26 (25 - 27)	ANDAHUAYLAS	2984	24 (23 - 26)
2016	Highlands	28 (28 - 28)	APURIMAC	26 (25 - 27)	ANTABAMBA	42	0
2016	Highlands	28 (28 - 28)	APURIMAC	26 (25 - 27)	AYMARAES	122	0
2016	Highlands	28 (28 - 28)	APURIMAC	26 (25 - 27)	CHINCHEROS	647	10 (8 - 13)
2016	Highlands	28 (28 - 28)	APURIMAC	26 (25 - 27)	COTABAMBAS	795	10 (8 - 12)
2016	Highlands	28 (28 - 28)	APURIMAC	26 (25 - 27)	GRAU	130	0
2016	Highlands	28 (28 - 28)	AREQUIPA	43 (42 - 43)	AREQUIPA	19705	46 (45 - 46)
2016	Highlands	28 (28 - 28)	AREQUIPA	43 (42 - 43)	CAMANA	1070	38 (35 - 41)
2016	Highlands	28 (28 - 28)	AREQUIPA	43 (42 - 43)	CARAVELI	152	0

2016	Highlands	28 (28 - 28)	AREQUIPA	43 (42 - 43)	CASTILLA	445	23 (19 - 27)
2016	Highlands	28 (28 - 28)	AREQUIPA	43 (42 - 43)	CAYLLOMA	1228	18 (16 - 20)
2016	Highlands	28 (28 - 28)	AREQUIPA	43 (42 - 43)	CONDESUYOS	65	0
2016	Highlands	28 (28 - 28)	AREQUIPA	43 (42 - 43)	ISLAY	422	36 (31 - 41)
2016	Highlands	28 (28 - 28)	AYACUCHO	25 (24 - 26)	CANGALLO	493	28 (24 - 32)
2016	Highlands	28 (28 - 28)	AYACUCHO	25 (24 - 26)	HUAMANGA	6793	32 (31 - 33)
2016	Highlands	28 (28 - 28)	AYACUCHO	25 (24 - 26)	HUANCA SANCOS	65	0
2016	Highlands	28 (28 - 28)	AYACUCHO	25 (24 - 26)	HUANTA	1581	16 (14 - 18)
2016	Highlands	28 (28 - 28)	AYACUCHO	25 (24 - 26)	LA MAR	1509	11 (9 - 13)
2016	Highlands	28 (28 - 28)	AYACUCHO	25 (24 - 26)	LUCANAS	501	16 (13 - 20)
2016	Highlands	28 (28 - 28)	AYACUCHO	25 (24 - 26)	PARINACOCHAS	418	22 (18 - 26)
2016	Highlands	28 (28 - 28)	AYACUCHO	25 (24 - 26)	PAUCAR DEL SARA SARA	59	0
2016	Highlands	28 (28 - 28)	AYACUCHO	25 (24 - 26)	SUCRE	52	0
2016	Highlands	28 (28 - 28)	AYACUCHO	25 (24 - 26)	VICTOR FAJARDO	59	0
2016	Highlands	28 (28 - 28)	AYACUCHO	25 (24 - 26)	VILCAS HUAMAN	146	0
2016	Highlands	28 (28 - 28)	CAJAMARCA	23 (22 - 23)	CAJABAMBA	886	19 (16 - 21)
2016	Highlands	28 (28 - 28)	CAJAMARCA	23 (22 - 23)	CAJAMARCA	7119	23 (22 - 24)
2016	Highlands	28 (28 - 28)	CAJAMARCA	23 (22 - 23)	CELENDIN	548	0
2016	Highlands	28 (28 - 28)	CAJAMARCA	23 (22 - 23)	CHOTA	1782	29 (27 - 31)
2016	Highlands	28 (28 - 28)	CAJAMARCA	23 (22 - 23)	CONTUMAZA	159	0
2016	Highlands	28 (28 - 28)	CAJAMARCA	23 (22 - 23)	CUTERVO	1093	27 (24 - 29)
2016	Highlands	28 (28 - 28)	CAJAMARCA	23 (22 - 23)	HUALGAYOC	753	10 (8 - 13)
2016	Highlands	28 (28 - 28)	CAJAMARCA	23 (22 - 23)	JAEN	3013	38 (36 - 40)
2016	Highlands	28 (28 - 28)	CAJAMARCA	23 (22 - 23)	SAN IGNACIO	824	0
2016	Highlands	28 (28 - 28)	CAJAMARCA	23 (22 - 23)	SAN MARCOS	343	0
2016	Highlands	28 (28 - 28)	CAJAMARCA	23 (22 - 23)	SAN MIGUEL	188	0
2016	Highlands	28 (28 - 28)	CAJAMARCA	23 (22 - 23)	SAN PABLO	141	0
2016	Highlands	28 (28 - 28)	CAJAMARCA	23 (22 - 23)	SANTA CRUZ	181	4 (2 - 8)
2016	Highlands	28 (28 - 28)	CUSCO	31 (31 - 32)	ANTA	276	0
2016	Highlands	28 (28 - 28)	CUSCO	31 (31 - 32)	CALCA	561	0
2016	Highlands	28 (28 - 28)	CUSCO	31 (31 - 32)	CANAS	142	0

2016	Highlands	28 (28 - 28)	CUSCO	31 (31 - 32)	CANCHIS	1796	23 (21 - 25)
2016	Highlands	28 (28 - 28)	CUSCO	31 (31 - 32)	CHUMBIVILCAS	613	17 (14 - 20)
2016	Highlands	28 (28 - 28)	CUSCO	31 (31 - 32)	CUSCO	12165	42 (41 - 43)
2016	Highlands	28 (28 - 28)	CUSCO	31 (31 - 32)	ESPINAR	967	12 (10 - 14)
2016	Highlands	28 (28 - 28)	CUSCO	31 (31 - 32)	LA CONVENCION	2204	25 (23 - 27)
2016	Highlands	28 (28 - 28)	CUSCO	31 (31 - 32)	PARURO	30	0
2016	Highlands	28 (28 - 28)	CUSCO	31 (31 - 32)	PAUCARTAMBO	256	0
2016	Highlands	28 (28 - 28)	CUSCO	31 (31 - 32)	QUISPICANCHI	554	0
2016	Highlands	28 (28 - 28)	CUSCO	31 (31 - 32)	URUBAMBA	398	0
2016	Highlands	28 (28 - 28)	HUANCAVELICA	18 (17 - 20)	ACOBAMBA	646	14 (11 - 17)
2016	Highlands	28 (28 - 28)	HUANCAVELICA	18 (17 - 20)	ANGARAES	703	14 (11 - 16)
2016	Highlands	28 (28 - 28)	HUANCAVELICA	18 (17 - 20)	CASTROVIRREYNA	74	0
2016	Highlands	28 (28 - 28)	HUANCAVELICA	18 (17 - 20)	CHURCAMPA	405	0
2016	Highlands	28 (28 - 28)	HUANCAVELICA	18 (17 - 20)	HUANCAVELICA	2225	26 (24 - 28)
2016	Highlands	28 (28 - 28)	HUANCAVELICA	18 (17 - 20)	HUAYTARA	106	0
2016	Highlands	28 (28 - 28)	HUANCAVELICA	18 (17 - 20)	TAYACAJA	880	19 (17 - 22)
2016	Highlands	28 (28 - 28)	HUANUCO	20 (19 - 21)	AMBO	597	0
2016	Highlands	28 (28 - 28)	HUANUCO	20 (19 - 21)	DOS DE MAYO	433	8 (5 - 11)
2016	Highlands	28 (28 - 28)	HUANUCO	20 (19 - 21)	HUACAYBAMBA	124	0
2016	Highlands	28 (28 - 28)	HUANUCO	20 (19 - 21)	HUAMALIES	679	6 (5 - 9)
2016	Highlands	28 (28 - 28)	HUANUCO	20 (19 - 21)	HUANUCO	6413	26 (24 - 27)
2016	Highlands	28 (28 - 28)	HUANUCO	20 (19 - 21)	LAURICOCHA	197	0
2016	Highlands	28 (28 - 28)	HUANUCO	20 (19 - 21)	LEONCIO PRADO	3044	30 (28 - 31)
2016	Highlands	28 (28 - 28)	HUANUCO	20 (19 - 21)	MARAÑON	83	0
2016	Highlands	28 (28 - 28)	HUANUCO	20 (19 - 21)	PACHITEA	850	0
2016	Highlands	28 (28 - 28)	HUANUCO	20 (19 - 21)	PUERTO INCA	329	0
2016	Highlands	28 (28 - 28)	HUANUCO	20 (19 - 21)	YAROWILCA	284	0
2016	Highlands	28 (28 - 28)	JUNIN	27 (26 - 27)	CHANCHAMAYO	2732	37 (36 - 39)
2016	Highlands	28 (28 - 28)	JUNIN	27 (26 - 27)	CHUPACA	497	0
2016	Highlands	28 (28 - 28)	JUNIN	27 (26 - 27)	CONCEPCION	356	0
2016	Highlands	28 (28 - 28)	JUNIN	27 (26 - 27)	HUANCAYO	9953	30 (29 - 31)

2016	Highlands	28 (28 - 28)	JUNIN	27 (26 - 27)	JAUJA	1066	26 (23 - 29)
2016	Highlands	28 (28 - 28)	JUNIN	27 (26 - 27)	JUNIN	408	12 (9 - 15)
2016	Highlands	28 (28 - 28)	JUNIN	27 (26 - 27)	SATIPO	2363	20 (19 - 22)
2016	Highlands	28 (28 - 28)	JUNIN	27 (26 - 27)	TARMA	1443	11 (10 - 13)
2016	Highlands	28 (28 - 28)	JUNIN	27 (26 - 27)	YAULI	406	29 (24 - 33)
2016	Highlands	28 (28 - 28)	PASCO	20 (19 - 21)	DANIEL ALCIDES CARRION	176	1 (0 - 3)
2016	Highlands	28 (28 - 28)	PASCO	20 (19 - 21)	OXAPAMPA	1696	13 (12 - 15)
2016	Highlands	28 (28 - 28)	PASCO	20 (19 - 21)	PASCO	2442	25 (24 - 27)
2016	Highlands	28 (28 - 28)	PUNO	22 (22 - 23)	AZANGARO	1214	9 (7 - 10)
2016	Highlands	28 (28 - 28)	PUNO	22 (22 - 23)	CARABAYA	460	8 (6 - 11)
2016	Highlands	28 (28 - 28)	PUNO	22 (22 - 23)	CHUCUITO	841	5 (3 - 6)
2016	Highlands	28 (28 - 28)	PUNO	22 (22 - 23)	EL COLLAO	751	6 (5 - 8)
2016	Highlands	28 (28 - 28)	PUNO	22 (22 - 23)	HUANCANE	594	13 (11 - 16)
2016	Highlands	28 (28 - 28)	PUNO	22 (22 - 23)	LAMPA	262	6 (4 - 10)
2016	Highlands	28 (28 - 28)	PUNO	22 (22 - 23)	MELGAR	910	20 (17 - 22)
2016	Highlands	28 (28 - 28)	PUNO	22 (22 - 23)	MOHO	80	0
2016	Highlands	28 (28 - 28)	PUNO	22 (22 - 23)	PUNO	3377	33 (31 - 34)
2016	Highlands	28 (28 - 28)	PUNO	22 (22 - 23)	SAN ANTONIO DE PUTINA	80	0
2016	Highlands	28 (28 - 28)	PUNO	22 (22 - 23)	SAN ROMAN	5930	28 (27 - 30)
2016	Highlands	28 (28 - 28)	PUNO	22 (22 - 23)	SANDIA	393	9 (7 - 13)
2016	Highlands	28 (28 - 28)	PUNO	22 (22 - 23)	YUNGUYO	383	14 (11 - 18)
2017	Amazon	27 (27 - 28)	AMAZONAS	29 (28 - 30)	BAGUA	1279	45 (43 - 48)
2017	Amazon	27 (27 - 28)	AMAZONAS	29 (28 - 30)	BONGARA	204	0
2017	Amazon	27 (27 - 28)	AMAZONAS	29 (28 - 30)	CHACHAPOYAS	1750	33 (31 - 35)
2017	Amazon	27 (27 - 28)	AMAZONAS	29 (28 - 30)	CONDORCANQUI	454	4 (2 - 6)
2017	Amazon	27 (27 - 28)	AMAZONAS	29 (28 - 30)	LUYA	168	0
2017	Amazon	27 (27 - 28)	AMAZONAS	29 (28 - 30)	RODRIGUEZ DE MENDOZA	166	0
2017	Amazon	27 (27 - 28)	AMAZONAS	29 (28 - 30)	UTCUBAMBA	1430	28 (25 - 30)
2017	Amazon	27 (27 - 28)	LORETO	25 (25 - 26)	ALTO AMAZONAS	3411	21 (20 - 23)
2017	Amazon	27 (27 - 28)	LORETO	25 (25 - 26)	DATEM DEL MARAÑON	485	0
2017	Amazon	27 (27 - 28)	LORETO	25 (25 - 26)	LORETO	669	0

2017	Amazon	27 (27 - 28)	LORETO	25 (25 - 26)	MARISCAL RAMON CASTILLA	861	2 (1 - 3)
2017	Amazon	27 (27 - 28)	LORETO	25 (25 - 26)	MAYNAS	10870	34 (34 - 35)
2017	Amazon	27 (27 - 28)	LORETO	25 (25 - 26)	PUTUMAYO	56	0
2017	Amazon	27 (27 - 28)	LORETO	25 (25 - 26)	REQUENA	695	0
2017	Amazon	27 (27 - 28)	LORETO	25 (25 - 26)	UCAYALI	845	6 (5 - 8)
2017	Amazon	27 (27 - 28)	MADRE DE DIOS	25 (24 - 27)	MANU	123	0
2017	Amazon	27 (27 - 28)	MADRE DE DIOS	25 (24 - 27)	TAHUAMANU	131	11 (6 - 17)
2017	Amazon	27 (27 - 28)	MADRE DE DIOS	25 (24 - 27)	TAMBOPATA	3414	27 (25 - 28)
2017	Amazon	27 (27 - 28)	SAN MARTIN	30 (29 - 31)	BELLAVISTA	600	0
2017	Amazon	27 (27 - 28)	SAN MARTIN	30 (29 - 31)	EL DORADO	489	0
2017	Amazon	27 (27 - 28)	SAN MARTIN	30 (29 - 31)	HUALLAGA	161	0
2017	Amazon	27 (27 - 28)	SAN MARTIN	30 (29 - 31)	LAMAS	836	0
2017	Amazon	27 (27 - 28)	SAN MARTIN	30 (29 - 31)	MARISCAL CACERES	1007	24 (22 - 27)
2017	Amazon	27 (27 - 28)	SAN MARTIN	30 (29 - 31)	MOYOBAMBA	2413	33 (31 - 35)
2017	Amazon	27 (27 - 28)	SAN MARTIN	30 (29 - 31)	PICOTA	462	0
2017	Amazon	27 (27 - 28)	SAN MARTIN	30 (29 - 31)	RIOJA	2457	27 (25 - 29)
2017	Amazon	27 (27 - 28)	SAN MARTIN	30 (29 - 31)	SAN MARTIN	5425	47 (46 - 48)
2017	Amazon	27 (27 - 28)	SAN MARTIN	30 (29 - 31)	TOCACHE	1385	21 (19 - 24)
2017	Amazon	27 (27 - 28)	UCAYALI	27 (26 - 28)	ATALAYA	711	4 (3 - 6)
2017	Amazon	27 (27 - 28)	UCAYALI	27 (26 - 28)	CORONEL PORTILLO	10585	31 (30 - 32)
2017	Amazon	27 (27 - 28)	UCAYALI	27 (26 - 28)	PADRE ABAD	904	0
2017	Amazon	27 (27 - 28)	UCAYALI	27 (26 - 28)	PURUS	63	0
2017	Coast	41 (40 - 41)	CALLAO	39 (38 - 39)	CALLAO	18553	39 (38 - 39)
2017	Coast	41 (40 - 41)	ICA	41 (41 - 42)	CHINCHA	3926	38 (36 - 39)
2017	Coast	41 (40 - 41)	ICA	41 (41 - 42)	ICA	7862	45 (44 - 47)
2017	Coast	41 (40 - 41)	ICA	41 (41 - 42)	NAZCA	1175	33 (31 - 36)
2017	Coast	41 (40 - 41)	ICA	41 (41 - 42)	PALPA	75	1 (0 - 7)
2017	Coast	41 (40 - 41)	ICA	41 (41 - 42)	PISCO	2996	40 (38 - 42)
2017	Coast	41 (40 - 41)	LA LIBERTAD	36 (35 - 36)	ASCOPE	1160	28 (25 - 30)
2017	Coast	41 (40 - 41)	LA LIBERTAD	36 (35 - 36)	BOLIVAR	35	0
2017	Coast	41 (40 - 41)	LA LIBERTAD	36 (35 - 36)	CHEPEN	1212	49 (46 - 52)

2017	Coast	41 (40 - 41)	LA LIBERTAD	36 (35 - 36)	GRAN CHIMU	94	0
2017	Coast	41 (40 - 41)	LA LIBERTAD	36 (35 - 36)	JULCAN	246	0
2017	Coast	41 (40 - 41)	LA LIBERTAD	36 (35 - 36)	OTUZCO	578	28 (24 - 32)
2017	Coast	41 (40 - 41)	LA LIBERTAD	36 (35 - 36)	PACASMAYO	1065	40 (37 - 43)
2017	Coast	41 (40 - 41)	LA LIBERTAD	36 (35 - 36)	PATAZ	681	0
2017	Coast	41 (40 - 41)	LA LIBERTAD	36 (35 - 36)	SANCHEZ CARRION	2142	17 (16 - 19)
2017	Coast	41 (40 - 41)	LA LIBERTAD	36 (35 - 36)	SANTIAGO DE CHUCO	554	20 (17 - 23)
2017	Coast	41 (40 - 41)	LA LIBERTAD	36 (35 - 36)	TRUJILLO	15801	43 (42 - 44)
2017	Coast	41 (40 - 41)	LA LIBERTAD	36 (35 - 36)	VIRU	922	0
2017	Coast	41 (40 - 41)	LAMBAYEQUE	33 (32 - 33)	CHICLAYO	13702	37 (37 - 38)
2017	Coast	41 (40 - 41)	LAMBAYEQUE	33 (32 - 33)	FERREÑAFE	799	4 (3 - 6)
2017	Coast	41 (40 - 41)	LAMBAYEQUE	33 (32 - 33)	LAMBAYEQUE	4645	23 (22 - 25)
2017	Coast	41 (40 - 41)	LIMA	43 (43 - 44)	BARRANCA	2224	37 (35 - 39)
2017	Coast	41 (40 - 41)	LIMA	43 (43 - 44)	CAÑETE	4258	32 (31 - 34)
2017	Coast	41 (40 - 41)	LIMA	43 (43 - 44)	HUARAL	2704	30 (28 - 32)
2017	Coast	41 (40 - 41)	LIMA	43 (43 - 44)	HUAROCHIRI	201	0
2017	Coast	41 (40 - 41)	LIMA	43 (43 - 44)	HUAURA	3498	38 (36 - 40)
2017	Coast	41 (40 - 41)	LIMA	43 (43 - 44)	LIMA	140026	44 (44 - 44)
2017	Coast	41 (40 - 41)	MOQUEGUA	42 (40 - 44)	ILO	1078	40 (37 - 43)
2017	Coast	41 (40 - 41)	MOQUEGUA	42 (40 - 44)	MARISCAL NIETO	1320	45 (42 - 48)
2017	Coast	41 (40 - 41)	PIURA	34 (33 - 34)	AYABACA	816	0
2017	Coast	41 (40 - 41)	PIURA	34 (33 - 34)	HUANCABAMBA	732	3 (2 - 5)
2017	Coast	41 (40 - 41)	PIURA	34 (33 - 34)	MORROPON	2326	35 (33 - 37)
2017	Coast	41 (40 - 41)	PIURA	34 (33 - 34)	PAITA	1775	28 (25 - 30)
2017	Coast	41 (40 - 41)	PIURA	34 (33 - 34)	PIURA	11254	37 (36 - 37)
2017	Coast	41 (40 - 41)	PIURA	34 (33 - 34)	SECHURA	632	1 (1 - 2)
2017	Coast	41 (40 - 41)	PIURA	34 (33 - 34)	SULLANA	5718	44 (43 - 46)
2017	Coast	41 (40 - 41)	PIURA	34 (33 - 34)	TALARA	703	13 (11 - 16)
2017	Coast	41 (40 - 41)	TACNA	47 (45 - 48)	TACNA	5084	47 (46 - 48)
2017	Coast	41 (40 - 41)	TUMBES	45 (44 - 47)	CONTRALMIRANTE VILLAR	105	0
2017	Coast	41 (40 - 41)	TUMBES	45 (44 - 47)	TUMBES	3784	50 (49 - 52)

2017	Coast	41 (40 - 41)	TUMBES	45 (44 - 47)	ZARUMILLA	304	0
2017	Highlands	29 (29 - 29)	ANCASH	31 (30 - 32)	ANTONIO RAIMONDI	78	0
2017	Highlands	29 (29 - 29)	ANCASH	31 (30 - 32)	ASUNCION	146	0
2017	Highlands	29 (29 - 29)	ANCASH	31 (30 - 32)	BOLOGNESI	35	0
2017	Highlands	29 (29 - 29)	ANCASH	31 (30 - 32)	CARHUAZ	520	9 (6 - 11)
2017	Highlands	29 (29 - 29)	ANCASH	31 (30 - 32)	CARLOS FERMIN FITZCARRALD	142	0
2017	Highlands	29 (29 - 29)	ANCASH	31 (30 - 32)	CASMA	235	12 (8 - 17)
2017	Highlands	29 (29 - 29)	ANCASH	31 (30 - 32)	HUARAZ	4206	36 (35 - 38)
2017	Highlands	29 (29 - 29)	ANCASH	31 (30 - 32)	HUARI	572	22 (19 - 26)
2017	Highlands	29 (29 - 29)	ANCASH	31 (30 - 32)	HUAYLAS	868	34 (31 - 38)
2017	Highlands	29 (29 - 29)	ANCASH	31 (30 - 32)	MARISCAL LUZURIAGA	82	0
2017	Highlands	29 (29 - 29)	ANCASH	31 (30 - 32)	PALLASCA	88	0
2017	Highlands	29 (29 - 29)	ANCASH	31 (30 - 32)	POMABAMBA	390	14 (11 - 18)
2017	Highlands	29 (29 - 29)	ANCASH	31 (30 - 32)	RECUAY	145	19 (13 - 27)
2017	Highlands	29 (29 - 29)	ANCASH	31 (30 - 32)	SANTA	7686	37 (36 - 38)
2017	Highlands	29 (29 - 29)	ANCASH	31 (30 - 32)	SIHUAS	245	0
2017	Highlands	29 (29 - 29)	ANCASH	31 (30 - 32)	YUNGAY	486	0
2017	Highlands	29 (29 - 29)	APURIMAC	26 (25 - 27)	ABANCAY	2720	38 (36 - 39)
2017	Highlands	29 (29 - 29)	APURIMAC	26 (25 - 27)	ANDAHUAYLAS	2953	25 (23 - 27)
2017	Highlands	29 (29 - 29)	APURIMAC	26 (25 - 27)	ANTABAMBA	60	0
2017	Highlands	29 (29 - 29)	APURIMAC	26 (25 - 27)	AYMARAES	135	0
2017	Highlands	29 (29 - 29)	APURIMAC	26 (25 - 27)	CHINCHEROS	635	14 (11 - 17)
2017	Highlands	29 (29 - 29)	APURIMAC	26 (25 - 27)	COTABAMBAS	749	13 (11 - 16)
2017	Highlands	29 (29 - 29)	APURIMAC	26 (25 - 27)	GRAU	123	0
2017	Highlands	29 (29 - 29)	AREQUIPA	43 (42 - 44)	AREQUIPA	19241	46 (46 - 47)
2017	Highlands	29 (29 - 29)	AREQUIPA	43 (42 - 44)	CAMANA	1070	37 (34 - 40)
2017	Highlands	29 (29 - 29)	AREQUIPA	43 (42 - 44)	CARAVELI	130	0
2017	Highlands	29 (29 - 29)	AREQUIPA	43 (42 - 44)	CASTILLA	418	28 (24 - 33)
2017	Highlands	29 (29 - 29)	AREQUIPA	43 (42 - 44)	CAYLLOMA	1380	19 (17 - 21)
2017	Highlands	29 (29 - 29)	AREQUIPA	43 (42 - 44)	CONDESUYOS	75	0
2017	Highlands	29 (29 - 29)	AREQUIPA	43 (42 - 44)	ISLAY	425	31 (26 - 35)

2017	Highlands	29 (29 - 29)	AREQUIPA	43 (42 - 44)	LA UNION	35	0
2017	Highlands	29 (29 - 29)	AYACUCHO	27 (26 - 28)	CANGALLO	509	23 (20 - 27)
2017	Highlands	29 (29 - 29)	AYACUCHO	27 (26 - 28)	HUAMANGA	7091	33 (32 - 34)
2017	Highlands	29 (29 - 29)	AYACUCHO	27 (26 - 28)	HUANCA SANCOS	53	0
2017	Highlands	29 (29 - 29)	AYACUCHO	27 (26 - 28)	HUANTA	1671	20 (18 - 22)
2017	Highlands	29 (29 - 29)	AYACUCHO	27 (26 - 28)	LA MAR	1838	22 (21 - 24)
2017	Highlands	29 (29 - 29)	AYACUCHO	27 (26 - 28)	LUCANAS	476	14 (11 - 18)
2017	Highlands	29 (29 - 29)	AYACUCHO	27 (26 - 28)	PARINACOCHAS	395	21 (17 - 26)
2017	Highlands	29 (29 - 29)	AYACUCHO	27 (26 - 28)	PAUCAR DEL SARA SARA	61	0
2017	Highlands	29 (29 - 29)	AYACUCHO	27 (26 - 28)	SUCRE	73	0
2017	Highlands	29 (29 - 29)	AYACUCHO	27 (26 - 28)	VICTOR FAJARDO	53	0
2017	Highlands	29 (29 - 29)	AYACUCHO	27 (26 - 28)	VILCAS HUAMAN	124	0
2017	Highlands	29 (29 - 29)	CAJAMARCA	24 (24 - 25)	CAJABAMBA	880	14 (12 - 16)
2017	Highlands	29 (29 - 29)	CAJAMARCA	24 (24 - 25)	CAJAMARCA	7443	26 (25 - 27)
2017	Highlands	29 (29 - 29)	CAJAMARCA	24 (24 - 25)	CELENDIN	607	0
2017	Highlands	29 (29 - 29)	CAJAMARCA	24 (24 - 25)	CHOTA	1952	31 (29 - 33)
2017	Highlands	29 (29 - 29)	CAJAMARCA	24 (24 - 25)	CONTUMAZA	163	0
2017	Highlands	29 (29 - 29)	CAJAMARCA	24 (24 - 25)	CUTERVO	1295	23 (20 - 25)
2017	Highlands	29 (29 - 29)	CAJAMARCA	24 (24 - 25)	HUALGAYOC	777	8 (6 - 10)
2017	Highlands	29 (29 - 29)	CAJAMARCA	24 (24 - 25)	JAEN	3334	41 (40 - 43)
2017	Highlands	29 (29 - 29)	CAJAMARCA	24 (24 - 25)	SAN IGNACIO	817	0
2017	Highlands	29 (29 - 29)	CAJAMARCA	24 (24 - 25)	SAN MARCOS	418	0
2017	Highlands	29 (29 - 29)	CAJAMARCA	24 (24 - 25)	SAN MIGUEL	161	0
2017	Highlands	29 (29 - 29)	CAJAMARCA	24 (24 - 25)	SAN PABLO	116	0
2017	Highlands	29 (29 - 29)	CAJAMARCA	24 (24 - 25)	SANTA CRUZ	172	7 (4 - 12)
2017	Highlands	29 (29 - 29)	CUSCO	31 (30 - 32)	ANTA	267	0
2017	Highlands	29 (29 - 29)	CUSCO	31 (30 - 32)	CALCA	512	0
2017	Highlands	29 (29 - 29)	CUSCO	31 (30 - 32)	CANAS	267	0
2017	Highlands	29 (29 - 29)	CUSCO	31 (30 - 32)	CANCHIS	1946	25 (23 - 27)
2017	Highlands	29 (29 - 29)	CUSCO	31 (30 - 32)	CHUMBIVILCAS	637	13 (10 - 16)
2017	Highlands	29 (29 - 29)	CUSCO	31 (30 - 32)	CUSCO	12585	42 (41 - 42)

2017	Highlands	29 (29 - 29)	CUSCO	31 (30 - 32)	ESPINAR	1013	14 (11 - 16)
2017	Highlands	29 (29 - 29)	CUSCO	31 (30 - 32)	LA CONVENCION	2373	24 (22 - 26)
2017	Highlands	29 (29 - 29)	CUSCO	31 (30 - 32)	PARURO	69	0
2017	Highlands	29 (29 - 29)	CUSCO	31 (30 - 32)	PAUCARTAMBO	272	0
2017	Highlands	29 (29 - 29)	CUSCO	31 (30 - 32)	QUISPICANCHI	581	0
2017	Highlands	29 (29 - 29)	CUSCO	31 (30 - 32)	URUBAMBA	395	0
2017	Highlands	29 (29 - 29)	HUANCAVELICA	19 (18 - 20)	ACOBAMBA	700	18 (15 - 21)
2017	Highlands	29 (29 - 29)	HUANCAVELICA	19 (18 - 20)	ANGARAES	718	13 (10 - 15)
2017	Highlands	29 (29 - 29)	HUANCAVELICA	19 (18 - 20)	CASTROVIRREYNA	78	0
2017	Highlands	29 (29 - 29)	HUANCAVELICA	19 (18 - 20)	CHURCAMPA	415	0
2017	Highlands	29 (29 - 29)	HUANCAVELICA	19 (18 - 20)	HUANCAVELICA	2352	27 (25 - 29)
2017	Highlands	29 (29 - 29)	HUANCAVELICA	19 (18 - 20)	HUAYTARA	73	0
2017	Highlands	29 (29 - 29)	HUANCAVELICA	19 (18 - 20)	TAYACAJA	1011	18 (15 - 20)
2017	Highlands	29 (29 - 29)	HUANUCO	22 (21 - 22)	AMBO	624	0
2017	Highlands	29 (29 - 29)	HUANUCO	22 (21 - 22)	DOS DE MAYO	512	6 (4 - 9)
2017	Highlands	29 (29 - 29)	HUANUCO	22 (21 - 22)	HUACAYBAMBA	138	0
2017	Highlands	29 (29 - 29)	HUANUCO	22 (21 - 22)	HUAMALIES	721	8 (6 - 10)
2017	Highlands	29 (29 - 29)	HUANUCO	22 (21 - 22)	HUANUCO	6871	29 (27 - 30)
2017	Highlands	29 (29 - 29)	HUANUCO	22 (21 - 22)	LAURICOCHA	209	0
2017	Highlands	29 (29 - 29)	HUANUCO	22 (21 - 22)	LEONCIO PRADO	3378	30 (28 - 31)
2017	Highlands	29 (29 - 29)	HUANUCO	22 (21 - 22)	PACHITEA	987	0
2017	Highlands	29 (29 - 29)	HUANUCO	22 (21 - 22)	PUERTO INCA	416	0
2017	Highlands	29 (29 - 29)	HUANUCO	22 (21 - 22)	YAROWILCA	251	0
2017	Highlands	29 (29 - 29)	JUNIN	30 (29 - 30)	CHANCHAMAYO	2907	33 (31 - 34)
2017	Highlands	29 (29 - 29)	JUNIN	30 (29 - 30)	CHUPACA	471	0
2017	Highlands	29 (29 - 29)	JUNIN	30 (29 - 30)	CONCEPCION	334	0
2017	Highlands	29 (29 - 29)	JUNIN	30 (29 - 30)	HUANCAYO	10290	36 (35 - 37)
2017	Highlands	29 (29 - 29)	JUNIN	30 (29 - 30)	JAUJA	1055	29 (26 - 32)
2017	Highlands	29 (29 - 29)	JUNIN	30 (29 - 30)	JUNIN	348	10 (7 - 14)
2017	Highlands	29 (29 - 29)	JUNIN	30 (29 - 30)	SATIPO	2607	28 (26 - 30)
2017	Highlands	29 (29 - 29)	JUNIN	30 (29 - 30)	TARMA	1399	9 (8 - 11)

2017	Highlands	29 (29 - 29)	JUNIN	30 (29 - 30)	YAULI	373	19 (15 - 23)
2017	Highlands	29 (29 - 29)	PASCO	20 (19 - 21)	DANIEL ALCIDES CARRION	179	0
2017	Highlands	29 (29 - 29)	PASCO	20 (19 - 21)	OXAPAMPA	1900	15 (14 - 17)
2017	Highlands	29 (29 - 29)	PASCO	20 (19 - 21)	PASCO	2489	25 (23 - 27)
2017	Highlands	29 (29 - 29)	PUNO	23 (22 - 23)	AZANGARO	1143	12 (10 - 14)
2017	Highlands	29 (29 - 29)	PUNO	23 (22 - 23)	CARABAYA	516	11 (9 - 15)
2017	Highlands	29 (29 - 29)	PUNO	23 (22 - 23)	CHUCUITO	769	5 (4 - 7)
2017	Highlands	29 (29 - 29)	PUNO	23 (22 - 23)	EL COLLAO	732	10 (8 - 12)
2017	Highlands	29 (29 - 29)	PUNO	23 (22 - 23)	HUANCANE	592	11 (8 - 14)
2017	Highlands	29 (29 - 29)	PUNO	23 (22 - 23)	LAMPA	236	5 (3 - 9)
2017	Highlands	29 (29 - 29)	PUNO	23 (22 - 23)	MELGAR	877	22 (19 - 25)
2017	Highlands	29 (29 - 29)	PUNO	23 (22 - 23)	MOHO	79	0
2017	Highlands	29 (29 - 29)	PUNO	23 (22 - 23)	PUNO	3484	32 (31 - 34)
2017	Highlands	29 (29 - 29)	PUNO	23 (22 - 23)	SAN ANTONIO DE PUTINA	247	0
2017	Highlands	29 (29 - 29)	PUNO	23 (22 - 23)	SAN ROMAN	6121	29 (28 - 31)
2017	Highlands	29 (29 - 29)	PUNO	23 (22 - 23)	SANDIA	462	5 (3 - 7)
2017	Highlands	29 (29 - 29)	PUNO	23 (22 - 23)	YUNGUYO	345	9 (6 - 13)
2018	Amazon	28 (28 - 29)	AMAZONAS	31 (30 - 33)	BAGUA	1193	44 (41 - 47)
2018	Amazon	28 (28 - 29)	AMAZONAS	31 (30 - 33)	BONGARA	173	0
2018	Amazon	28 (28 - 29)	AMAZONAS	31 (30 - 33)	CHACHAPOYAS	1727	36 (34 - 39)
2018	Amazon	28 (28 - 29)	AMAZONAS	31 (30 - 33)	CONDORCANQUI	428	7 (4 - 9)
2018	Amazon	28 (28 - 29)	AMAZONAS	31 (30 - 33)	LUYA	166	0
2018	Amazon	28 (28 - 29)	AMAZONAS	31 (30 - 33)	RODRIGUEZ DE MENDOZA	130	0
2018	Amazon	28 (28 - 29)	AMAZONAS	31 (30 - 33)	UTCUBAMBA	1388	33 (31 - 36)
2018	Amazon	28 (28 - 29)	LORETO	26 (26 - 27)	ALTO AMAZONAS	3299	22 (20 - 23)
2018	Amazon	28 (28 - 29)	LORETO	26 (26 - 27)	DATEM DEL MARAÑON	582	0
2018	Amazon	28 (28 - 29)	LORETO	26 (26 - 27)	LORETO	656	0
2018	Amazon	28 (28 - 29)	LORETO	26 (26 - 27)	MARISCAL RAMON CASTILLA	974	4 (3 - 5)
2018	Amazon	28 (28 - 29)	LORETO	26 (26 - 27)	MAYNAS	10883	35 (34 - 36)
2018	Amazon	28 (28 - 29)	LORETO	26 (26 - 27)	PUTUMAYO	53	0
2018	Amazon	28 (28 - 29)	LORETO	26 (26 - 27)	REQUENA	790	12 (9 - 14)

2018	Amazon	28 (28 - 29)	LORETO	26 (26 - 27)	UCAYALI	715	7 (5 - 9)
2018	Amazon	28 (28 - 29)	MADRE DE DIOS	26 (25 - 28)	MANU	107	0
2018	Amazon	28 (28 - 29)	MADRE DE DIOS	26 (25 - 28)	TAHUAMANU	126	16 (10 - 23)
2018	Amazon	28 (28 - 29)	MADRE DE DIOS	26 (25 - 28)	TAMBOPATA	3582	27 (26 - 29)
2018	Amazon	28 (28 - 29)	SAN MARTIN	31 (30 - 32)	BELLAVISTA	563	0
2018	Amazon	28 (28 - 29)	SAN MARTIN	31 (30 - 32)	EL DORADO	470	0
2018	Amazon	28 (28 - 29)	SAN MARTIN	31 (30 - 32)	HUALLAGA	164	0
2018	Amazon	28 (28 - 29)	SAN MARTIN	31 (30 - 32)	LAMAS	660	0
2018	Amazon	28 (28 - 29)	SAN MARTIN	31 (30 - 32)	MARISCAL CACERES	923	26 (24 - 29)
2018	Amazon	28 (28 - 29)	SAN MARTIN	31 (30 - 32)	MOYOBAMBA	2463	36 (34 - 38)
2018	Amazon	28 (28 - 29)	SAN MARTIN	31 (30 - 32)	PICOTA	418	0
2018	Amazon	28 (28 - 29)	SAN MARTIN	31 (30 - 32)	RIOJA	2222	27 (25 - 29)
2018	Amazon	28 (28 - 29)	SAN MARTIN	31 (30 - 32)	SAN MARTIN	5193	47 (46 - 49)
2018	Amazon	28 (28 - 29)	SAN MARTIN	31 (30 - 32)	TOCACHE	1350	22 (20 - 24)
2018	Amazon	28 (28 - 29)	UCAYALI	28 (27 - 29)	ATALAYA	716	0
2018	Amazon	28 (28 - 29)	UCAYALI	28 (27 - 29)	CORONEL PORTILLO	10217	33 (32 - 34)
2018	Amazon	28 (28 - 29)	UCAYALI	28 (27 - 29)	PADRE ABAD	976	1 (1 - 2)
2018	Amazon	28 (28 - 29)	UCAYALI	28 (27 - 29)	PURUS	45	0
2018	Coast	43 (42 - 43)	CALLAO	41 (40 - 41)	CALLAO	20024	41 (40 - 41)
2018	Coast	43 (42 - 43)	ICA	42 (42 - 43)	CHINCHA	4174	35 (33 - 36)
2018	Coast	43 (42 - 43)	ICA	42 (42 - 43)	ICA	8105	47 (46 - 48)
2018	Coast	43 (42 - 43)	ICA	42 (42 - 43)	NAZCA	1130	37 (34 - 40)
2018	Coast	43 (42 - 43)	ICA	42 (42 - 43)	PALPA	65	0
2018	Coast	43 (42 - 43)	ICA	42 (42 - 43)	PISCO	2829	43 (41 - 44)
2018	Coast	43 (42 - 43)	LA LIBERTAD	37 (36 - 37)	ASCOPE	1244	30 (27 - 32)
2018	Coast	43 (42 - 43)	LA LIBERTAD	37 (36 - 37)	BOLIVAR	68	0
2018	Coast	43 (42 - 43)	LA LIBERTAD	37 (36 - 37)	CHEPEN	1264	44 (41 - 46)
2018	Coast	43 (42 - 43)	LA LIBERTAD	37 (36 - 37)	GRAN CHIMU	137	0
2018	Coast	43 (42 - 43)	LA LIBERTAD	37 (36 - 37)	JULCAN	236	0
2018	Coast	43 (42 - 43)	LA LIBERTAD	37 (36 - 37)	OTUZCO	529	28 (24 - 32)
2018	Coast	43 (42 - 43)	LA LIBERTAD	37 (36 - 37)	PACASMAYO	1068	41 (38 - 44)

2018	Coast	43 (42 - 43)	LA LIBERTAD	37 (36 - 37)	PATAZ	678	0
2018	Coast	43 (42 - 43)	LA LIBERTAD	37 (36 - 37)	SANCHEZ CARRION	2296	20 (19 - 22)
2018	Coast	43 (42 - 43)	LA LIBERTAD	37 (36 - 37)	SANTIAGO DE CHUCO	526	19 (16 - 23)
2018	Coast	43 (42 - 43)	LA LIBERTAD	37 (36 - 37)	TRUJILLO	16624	44 (43 - 45)
2018	Coast	43 (42 - 43)	LA LIBERTAD	37 (36 - 37)	VIRU	991	0
2018	Coast	43 (42 - 43)	LAMBAYEQUE	35 (34 - 35)	CHICLAYO	13753	41 (40 - 41)
2018	Coast	43 (42 - 43)	LAMBAYEQUE	35 (34 - 35)	FERREÑAFE	854	7 (5 - 9)
2018	Coast	43 (42 - 43)	LAMBAYEQUE	35 (34 - 35)	LAMBAYEQUE	4974	23 (22 - 25)
2018	Coast	43 (42 - 43)	LIMA	45 (45 - 46)	BARRANCA	2223	41 (38 - 43)
2018	Coast	43 (42 - 43)	LIMA	45 (45 - 46)	CAÑETE	4110	36 (34 - 37)
2018	Coast	43 (42 - 43)	LIMA	45 (45 - 46)	HUARAL	2742	30 (28 - 32)
2018	Coast	43 (42 - 43)	LIMA	45 (45 - 46)	HUAROCHIRI	217	3 (1 - 6)
2018	Coast	43 (42 - 43)	LIMA	45 (45 - 46)	HUAURA	4768	38 (37 - 40)
2018	Coast	43 (42 - 43)	LIMA	45 (45 - 46)	LIMA	148099	46 (46 - 46)
2018	Coast	43 (42 - 43)	MOQUEGUA	48 (46 - 50)	ILO	997	48 (45 - 51)
2018	Coast	43 (42 - 43)	MOQUEGUA	48 (46 - 50)	MARISCAL NIETO	1216	50 (47 - 52)
2018	Coast	43 (42 - 43)	PIURA	38 (37 - 39)	AYABACA	795	0
2018	Coast	43 (42 - 43)	PIURA	38 (37 - 39)	HUANCABAMBA	786	1 (0 - 2)
2018	Coast	43 (42 - 43)	PIURA	38 (37 - 39)	MORROPON	2399	33 (31 - 35)
2018	Coast	43 (42 - 43)	PIURA	38 (37 - 39)	PAITA	1958	28 (26 - 30)
2018	Coast	43 (42 - 43)	PIURA	38 (37 - 39)	PIURA	12430	39 (38 - 40)
2018	Coast	43 (42 - 43)	PIURA	38 (37 - 39)	SECHURA	610	2 (1 - 4)
2018	Coast	43 (42 - 43)	PIURA	38 (37 - 39)	SULLANA	6701	55 (53 - 56)
2018	Coast	43 (42 - 43)	PIURA	38 (37 - 39)	TALARA	1474	33 (30 - 35)
2018	Coast	43 (42 - 43)	TACNA	48 (46 - 49)	TACNA	5004	48 (47 - 49)
2018	Coast	43 (42 - 43)	TUMBES	46 (44 - 47)	CONTRALMIRANTE VILLAR	124	0
2018	Coast	43 (42 - 43)	TUMBES	46 (44 - 47)	TUMBES	3739	51 (49 - 53)
2018	Coast	43 (42 - 43)	TUMBES	46 (44 - 47)	ZARUMILLA	329	0
2018	Highlands	30 (30 - 30)	ANCASH	34 (33 - 35)	ANTONIO RAIMONDI	70	0
2018	Highlands	30 (30 - 30)	ANCASH	34 (33 - 35)	ASUNCION	120	0
2018	Highlands	30 (30 - 30)	ANCASH	34 (33 - 35)	BOLOGNESI	128	0

2018	Highlands	30 (30 - 30)	ANCASH	34 (33 - 35)	CARHUAZ	491	9 (6 - 11)
2018	Highlands	30 (30 - 30)	ANCASH	34 (33 - 35)	CARLOS FERMIN FITZCARRALD	118	0
2018	Highlands	30 (30 - 30)	ANCASH	34 (33 - 35)	CASMA	467	17 (14 - 21)
2018	Highlands	30 (30 - 30)	ANCASH	34 (33 - 35)	HUARAZ	4025	39 (38 - 41)
2018	Highlands	30 (30 - 30)	ANCASH	34 (33 - 35)	HUARI	616	11 (8 - 13)
2018	Highlands	30 (30 - 30)	ANCASH	34 (33 - 35)	HUARMEY	111	5 (2 - 11)
2018	Highlands	30 (30 - 30)	ANCASH	34 (33 - 35)	HUAYLAS	813	30 (27 - 34)
2018	Highlands	30 (30 - 30)	ANCASH	34 (33 - 35)	MARISCAL LUZURIAGA	62	0
2018	Highlands	30 (30 - 30)	ANCASH	34 (33 - 35)	PALLASCA	134	0
2018	Highlands	30 (30 - 30)	ANCASH	34 (33 - 35)	POMABAMBA	382	14 (11 - 18)
2018	Highlands	30 (30 - 30)	ANCASH	34 (33 - 35)	RECUAY	117	14 (8 - 21)
2018	Highlands	30 (30 - 30)	ANCASH	34 (33 - 35)	SANTA	8595	43 (42 - 44)
2018	Highlands	30 (30 - 30)	ANCASH	34 (33 - 35)	SIHUAS	253	0
2018	Highlands	30 (30 - 30)	ANCASH	34 (33 - 35)	YUNGAY	472	0
2018	Highlands	30 (30 - 30)	APURIMAC	27 (26 - 28)	ABANCAY	2807	35 (33 - 37)
2018	Highlands	30 (30 - 30)	APURIMAC	27 (26 - 28)	ANDAHUAYLAS	2816	27 (26 - 29)
2018	Highlands	30 (30 - 30)	APURIMAC	27 (26 - 28)	AYMARAES	125	0
2018	Highlands	30 (30 - 30)	APURIMAC	27 (26 - 28)	CHINCHEROS	620	9 (7 - 12)
2018	Highlands	30 (30 - 30)	APURIMAC	27 (26 - 28)	COTABAMBAS	767	17 (14 - 20)
2018	Highlands	30 (30 - 30)	APURIMAC	27 (26 - 28)	GRAU	89	0
2018	Highlands	30 (30 - 30)	AREQUIPA	44 (44 - 45)	AREQUIPA	19220	47 (46 - 48)
2018	Highlands	30 (30 - 30)	AREQUIPA	44 (44 - 45)	CAMANA	1049	37 (34 - 40)
2018	Highlands	30 (30 - 30)	AREQUIPA	44 (44 - 45)	CARAVELI	166	0
2018	Highlands	30 (30 - 30)	AREQUIPA	44 (44 - 45)	CASTILLA	398	33 (28 - 38)
2018	Highlands	30 (30 - 30)	AREQUIPA	44 (44 - 45)	CAYLLOMA	1329	22 (20 - 25)
2018	Highlands	30 (30 - 30)	AREQUIPA	44 (44 - 45)	CONDESUYOS	80	0
2018	Highlands	30 (30 - 30)	AREQUIPA	44 (44 - 45)	ISLAY	365	39 (34 - 44)
2018	Highlands	30 (30 - 30)	AREQUIPA	44 (44 - 45)	LA UNION	60	0
2018	Highlands	30 (30 - 30)	AYACUCHO	26 (26 - 27)	CANGALLO	497	21 (18 - 25)
2018	Highlands	30 (30 - 30)	AYACUCHO	26 (26 - 27)	HUAMANGA	6903	32 (31 - 33)
2018	Highlands	30 (30 - 30)	AYACUCHO	26 (26 - 27)	HUANCA SANCOS	63	0

2018	Highlands	30 (30 - 30)	AYACUCHO	26 (26 - 27)	HUANTA	1587	20 (18 - 22)
2018	Highlands	30 (30 - 30)	AYACUCHO	26 (26 - 27)	LA MAR	1662	21 (19 - 23)
2018	Highlands	30 (30 - 30)	AYACUCHO	26 (26 - 27)	LUCANAS	458	14 (11 - 18)
2018	Highlands	30 (30 - 30)	AYACUCHO	26 (26 - 27)	PARINACOCHAS	386	24 (19 - 28)
2018	Highlands	30 (30 - 30)	AYACUCHO	26 (26 - 27)	PAUCAR DEL SARA SARA	39	0
2018	Highlands	30 (30 - 30)	AYACUCHO	26 (26 - 27)	SUCRE	62	0
2018	Highlands	30 (30 - 30)	AYACUCHO	26 (26 - 27)	VICTOR FAJARDO	61	0
2018	Highlands	30 (30 - 30)	AYACUCHO	26 (26 - 27)	VILCAS HUAMAN	102	0
2018	Highlands	30 (30 - 30)	CAJAMARCA	25 (24 - 26)	CAJABAMBA	845	14 (12 - 16)
2018	Highlands	30 (30 - 30)	CAJAMARCA	25 (24 - 26)	CAJAMARCA	7259	25 (24 - 26)
2018	Highlands	30 (30 - 30)	CAJAMARCA	25 (24 - 26)	CELENDIN	574	1 (0 - 2)
2018	Highlands	30 (30 - 30)	CAJAMARCA	25 (24 - 26)	CHOTA	1963	31 (29 - 34)
2018	Highlands	30 (30 - 30)	CAJAMARCA	25 (24 - 26)	CONTUMAZA	134	0
2018	Highlands	30 (30 - 30)	CAJAMARCA	25 (24 - 26)	CUTERVO	1395	17 (15 - 19)
2018	Highlands	30 (30 - 30)	CAJAMARCA	25 (24 - 26)	HUALGAYOC	766	9 (7 - 11)
2018	Highlands	30 (30 - 30)	CAJAMARCA	25 (24 - 26)	JAEN	3710	45 (44 - 47)
2018	Highlands	30 (30 - 30)	CAJAMARCA	25 (24 - 26)	SAN IGNACIO	812	0
2018	Highlands	30 (30 - 30)	CAJAMARCA	25 (24 - 26)	SAN MARCOS	350	0
2018	Highlands	30 (30 - 30)	CAJAMARCA	25 (24 - 26)	SAN MIGUEL	157	0
2018	Highlands	30 (30 - 30)	CAJAMARCA	25 (24 - 26)	SAN PABLO	109	0
2018	Highlands	30 (30 - 30)	CAJAMARCA	25 (24 - 26)	SANTA CRUZ	173	2 (0 - 5)
2018	Highlands	30 (30 - 30)	CUSCO	31 (30 - 32)	ACOMAYO	99	0
2018	Highlands	30 (30 - 30)	CUSCO	31 (30 - 32)	ANTA	269	0
2018	Highlands	30 (30 - 30)	CUSCO	31 (30 - 32)	CALCA	527	0
2018	Highlands	30 (30 - 30)	CUSCO	31 (30 - 32)	CANAS	216	0
2018	Highlands	30 (30 - 30)	CUSCO	31 (30 - 32)	CANCHIS	1841	27 (25 - 29)
2018	Highlands	30 (30 - 30)	CUSCO	31 (30 - 32)	CHUMBIVILCAS	849	12 (10 - 14)
2018	Highlands	30 (30 - 30)	CUSCO	31 (30 - 32)	CUSCO	12344	41 (40 - 42)
2018	Highlands	30 (30 - 30)	CUSCO	31 (30 - 32)	ESPINAR	980	17 (15 - 20)
2018	Highlands	30 (30 - 30)	CUSCO	31 (30 - 32)	LA CONVENCION	2337	27 (25 - 29)
2018	Highlands	30 (30 - 30)	CUSCO	31 (30 - 32)	PARURO	36	0

2018	Highlands	30 (30 - 30)	CUSCO	31 (30 - 32)	PAUCARTAMBO	241	0
2018	Highlands	30 (30 - 30)	CUSCO	31 (30 - 32)	QUISPICANCHI	766	0
2018	Highlands	30 (30 - 30)	CUSCO	31 (30 - 32)	URUBAMBA	364	0
2018	Highlands	30 (30 - 30)	HUANCAVELICA	19 (18 - 20)	ACOBAMBA	569	15 (12 - 18)
2018	Highlands	30 (30 - 30)	HUANCAVELICA	19 (18 - 20)	ANGARAES	710	16 (14 - 19)
2018	Highlands	30 (30 - 30)	HUANCAVELICA	19 (18 - 20)	CASTROVIRREYNA	67	0
2018	Highlands	30 (30 - 30)	HUANCAVELICA	19 (18 - 20)	CHURCAMPA	423	0
2018	Highlands	30 (30 - 30)	HUANCAVELICA	19 (18 - 20)	HUANCAVELICA	2275	28 (26 - 29)
2018	Highlands	30 (30 - 30)	HUANCAVELICA	19 (18 - 20)	HUAYTARA	81	0
2018	Highlands	30 (30 - 30)	HUANCAVELICA	19 (18 - 20)	TAYACAJA	1063	16 (14 - 18)
2018	Highlands	30 (30 - 30)	HUANUCO	22 (21 - 23)	AMBO	650	0
2018	Highlands	30 (30 - 30)	HUANUCO	22 (21 - 23)	DOS DE MAYO	467	6 (4 - 9)
2018	Highlands	30 (30 - 30)	HUANUCO	22 (21 - 23)	HUACAYBAMBA	180	0
2018	Highlands	30 (30 - 30)	HUANUCO	22 (21 - 23)	HUAMALIES	699	8 (6 - 10)
2018	Highlands	30 (30 - 30)	HUANUCO	22 (21 - 23)	HUANUCO	6841	30 (29 - 31)
2018	Highlands	30 (30 - 30)	HUANUCO	22 (21 - 23)	LAURICOCHA	204	0
2018	Highlands	30 (30 - 30)	HUANUCO	22 (21 - 23)	LEONCIO PRADO	3184	28 (26 - 29)
2018	Highlands	30 (30 - 30)	HUANUCO	22 (21 - 23)	MARAÑON	100	0
2018	Highlands	30 (30 - 30)	HUANUCO	22 (21 - 23)	PACHITEA	791	0
2018	Highlands	30 (30 - 30)	HUANUCO	22 (21 - 23)	PUERTO INCA	419	0
2018	Highlands	30 (30 - 30)	HUANUCO	22 (21 - 23)	YAROWILCA	268	0
2018	Highlands	30 (30 - 30)	JUNIN	32 (31 - 33)	CHANCHAMAYO	2894	37 (35 - 38)
2018	Highlands	30 (30 - 30)	JUNIN	32 (31 - 33)	CHUPACA	414	0
2018	Highlands	30 (30 - 30)	JUNIN	32 (31 - 33)	CONCEPCION	309	0
2018	Highlands	30 (30 - 30)	JUNIN	32 (31 - 33)	HUANCAYO	10266	37 (36 - 38)
2018	Highlands	30 (30 - 30)	JUNIN	32 (31 - 33)	JAUJA	978	27 (25 - 30)
2018	Highlands	30 (30 - 30)	JUNIN	32 (31 - 33)	JUNIN	329	17 (13 - 22)
2018	Highlands	30 (30 - 30)	JUNIN	32 (31 - 33)	SATIPO	2614	29 (27 - 30)
2018	Highlands	30 (30 - 30)	JUNIN	32 (31 - 33)	TARMA	1374	13 (11 - 15)
2018	Highlands	30 (30 - 30)	JUNIN	32 (31 - 33)	YAULI	402	21 (17 - 25)
2018	Highlands	30 (30 - 30)	PASCO	20 (19 - 22)	DANIEL ALCIDES CARRION	181	0

2018	Highlands	30 (30 - 30)	PASCO	20 (19 - 22)	OXAPAMPA	1688	15 (13 - 17)
2018	Highlands	30 (30 - 30)	PASCO	20 (19 - 22)	PASCO	2276	26 (24 - 28)
2018	Highlands	30 (30 - 30)	PUNO	25 (24 - 26)	AZANGARO	1106	13 (11 - 15)
2018	Highlands	30 (30 - 30)	PUNO	25 (24 - 26)	CARABAYA	661	8 (6 - 10)
2018	Highlands	30 (30 - 30)	PUNO	25 (24 - 26)	CHUCUITO	714	7 (5 - 9)
2018	Highlands	30 (30 - 30)	PUNO	25 (24 - 26)	EL COLLAO	762	18 (15 - 20)
2018	Highlands	30 (30 - 30)	PUNO	25 (24 - 26)	HUANCANE	643	18 (15 - 21)
2018	Highlands	30 (30 - 30)	PUNO	25 (24 - 26)	LAMPA	272	7 (5 - 11)
2018	Highlands	30 (30 - 30)	PUNO	25 (24 - 26)	MELGAR	883	18 (15 - 21)
2018	Highlands	30 (30 - 30)	PUNO	25 (24 - 26)	MOHO	81	0
2018	Highlands	30 (30 - 30)	PUNO	25 (24 - 26)	PUNO	3263	33 (32 - 35)
2018	Highlands	30 (30 - 30)	PUNO	25 (24 - 26)	SAN ANTONIO DE PUTINA	227	0
2018	Highlands	30 (30 - 30)	PUNO	25 (24 - 26)	SAN ROMAN	6321	34 (33 - 35)
2018	Highlands	30 (30 - 30)	PUNO	25 (24 - 26)	SANDIA	487	4 (2 - 6)
2018	Highlands	30 (30 - 30)	PUNO	25 (24 - 26)	YUNGUYO	328	10 (7 - 14)
2019	Amazon	29 (28 - 29)	AMAZONAS	34 (33 - 35)	BAGUA	1282	44 (41 - 47)
2019	Amazon	29 (28 - 29)	AMAZONAS	34 (33 - 35)	BONGARA	186	0
2019	Amazon	29 (28 - 29)	AMAZONAS	34 (33 - 35)	CHACHAPOYAS	1805	39 (36 - 41)
2019	Amazon	29 (28 - 29)	AMAZONAS	34 (33 - 35)	CONDORCANQUI	377	11 (8 - 15)
2019	Amazon	29 (28 - 29)	AMAZONAS	34 (33 - 35)	LUYA	114	0
2019	Amazon	29 (28 - 29)	AMAZONAS	34 (33 - 35)	RODRIGUEZ DE MENDOZA	100	0
2019	Amazon	29 (28 - 29)	AMAZONAS	34 (33 - 35)	UTCUBAMBA	1330	34 (32 - 37)
2019	Amazon	29 (28 - 29)	LORETO	26 (25 - 27)	ALTO AMAZONAS	3322	22 (20 - 23)
2019	Amazon	29 (28 - 29)	LORETO	26 (25 - 27)	DATEM DEL MARAÑON	530	0
2019	Amazon	29 (28 - 29)	LORETO	26 (25 - 27)	LORETO	746	0
2019	Amazon	29 (28 - 29)	LORETO	26 (25 - 27)	MARISCAL RAMON CASTILLA	926	8 (6 - 10)
2019	Amazon	29 (28 - 29)	LORETO	26 (25 - 27)	MAYNAS	10851	35 (34 - 36)
2019	Amazon	29 (28 - 29)	LORETO	26 (25 - 27)	PUTUMAYO	45	0
2019	Amazon	29 (28 - 29)	LORETO	26 (25 - 27)	REQUENA	665	0
2019	Amazon	29 (28 - 29)	LORETO	26 (25 - 27)	UCAYALI	799	2 (1 - 3)
2019	Amazon	29 (28 - 29)	MADRE DE DIOS	28 (26 - 29)	MANU	83	5 (1 - 12)

2019	Amazon	29 (28 - 29)	MADRE DE DIOS	28 (26 - 29)	TAHUAMANU	134	10 (5 - 16)
2019	Amazon	29 (28 - 29)	MADRE DE DIOS	28 (26 - 29)	TAMBOPATA	3528	29 (28 - 31)
2019	Amazon	29 (28 - 29)	SAN MARTIN	32 (31 - 32)	BELLAVISTA	522	0
2019	Amazon	29 (28 - 29)	SAN MARTIN	32 (31 - 32)	EL DORADO	423	0
2019	Amazon	29 (28 - 29)	SAN MARTIN	32 (31 - 32)	HUALLAGA	170	0
2019	Amazon	29 (28 - 29)	SAN MARTIN	32 (31 - 32)	LAMAS	712	0
2019	Amazon	29 (28 - 29)	SAN MARTIN	32 (31 - 32)	MARISCAL CACERES	1004	31 (28 - 34)
2019	Amazon	29 (28 - 29)	SAN MARTIN	32 (31 - 32)	MOYOBAMBA	2508	37 (35 - 39)
2019	Amazon	29 (28 - 29)	SAN MARTIN	32 (31 - 32)	PICOTA	391	0
2019	Amazon	29 (28 - 29)	SAN MARTIN	32 (31 - 32)	RIOJA	2175	30 (28 - 32)
2019	Amazon	29 (28 - 29)	SAN MARTIN	32 (31 - 32)	SAN MARTIN	5483	45 (44 - 46)
2019	Amazon	29 (28 - 29)	SAN MARTIN	32 (31 - 32)	TOCACHE	1370	23 (20 - 25)
2019	Amazon	29 (28 - 29)	UCAYALI	28 (27 - 28)	ATALAYA	824	3 (2 - 4)
2019	Amazon	29 (28 - 29)	UCAYALI	28 (27 - 28)	CORONEL PORTILLO	10758	32 (31 - 33)
2019	Amazon	29 (28 - 29)	UCAYALI	28 (27 - 28)	PADRE ABAD	980	0
2019	Amazon	29 (28 - 29)	UCAYALI	28 (27 - 28)	PURUS	60	0
2019	Coast	44 (44 - 44)	CALLAO	43 (42 - 44)	CALLAO	19281	43 (42 - 44)
2019	Coast	44 (44 - 44)	ICA	43 (42 - 44)	CHINCHA	4118	35 (33 - 36)
2019	Coast	44 (44 - 44)	ICA	43 (42 - 44)	ICA	7814	48 (47 - 49)
2019	Coast	44 (44 - 44)	ICA	43 (42 - 44)	NAZCA	1122	38 (35 - 41)
2019	Coast	44 (44 - 44)	ICA	43 (42 - 44)	PALPA	59	0
2019	Coast	44 (44 - 44)	ICA	43 (42 - 44)	PISCO	2921	46 (44 - 47)
2019	Coast	44 (44 - 44)	LA LIBERTAD	37 (36 - 38)	ASCOPE	1080	34 (31 - 37)
2019	Coast	44 (44 - 44)	LA LIBERTAD	37 (36 - 38)	BOLIVAR	94	0
2019	Coast	44 (44 - 44)	LA LIBERTAD	37 (36 - 38)	CHEPEN	1126	45 (42 - 48)
2019	Coast	44 (44 - 44)	LA LIBERTAD	37 (36 - 38)	GRAN CHIMU	132	0
2019	Coast	44 (44 - 44)	LA LIBERTAD	37 (36 - 38)	JULCAN	229	0
2019	Coast	44 (44 - 44)	LA LIBERTAD	37 (36 - 38)	OTUZCO	503	27 (23 - 31)
2019	Coast	44 (44 - 44)	LA LIBERTAD	37 (36 - 38)	PACASMAYO	1038	43 (40 - 46)
2019	Coast	44 (44 - 44)	LA LIBERTAD	37 (36 - 38)	PATAZ	679	0
2019	Coast	44 (44 - 44)	LA LIBERTAD	37 (36 - 38)	SANCHEZ CARRION	2154	18 (16 - 19)

2019	Coast	44 (44 - 44)	LA LIBERTAD	37 (36 - 38)	SANTIAGO DE CHUCO	556	29 (25 - 33)
2019	Coast	44 (44 - 44)	LA LIBERTAD	37 (36 - 38)	TRUJILLO	17084	44 (44 - 45)
2019	Coast	44 (44 - 44)	LA LIBERTAD	37 (36 - 38)	VIRU	1164	0
2019	Coast	44 (44 - 44)	LAMBAYEQUE	37 (36 - 37)	CHICLAYO	13508	43 (42 - 44)
2019	Coast	44 (44 - 44)	LAMBAYEQUE	37 (36 - 37)	FERREÑAFE	765	3 (2 - 5)
2019	Coast	44 (44 - 44)	LAMBAYEQUE	37 (36 - 37)	LAMBAYEQUE	4737	24 (23 - 25)
2019	Coast	44 (44 - 44)	LIMA	47 (46 - 47)	BARRANCA	1978	39 (37 - 42)
2019	Coast	44 (44 - 44)	LIMA	47 (46 - 47)	CAÑETE	3953	35 (34 - 37)
2019	Coast	44 (44 - 44)	LIMA	47 (46 - 47)	HUARAL	2706	33 (31 - 35)
2019	Coast	44 (44 - 44)	LIMA	47 (46 - 47)	HUAROCHIRI	230	5 (2 - 8)
2019	Coast	44 (44 - 44)	LIMA	47 (46 - 47)	HUAURA	4488	40 (39 - 42)
2019	Coast	44 (44 - 44)	LIMA	47 (46 - 47)	LIMA	143799	48 (47 - 48)
2019	Coast	44 (44 - 44)	MOQUEGUA	47 (45 - 49)	ILO	1028	49 (46 - 52)
2019	Coast	44 (44 - 44)	MOQUEGUA	47 (45 - 49)	MARISCAL NIETO	1228	47 (44 - 49)
2019	Coast	44 (44 - 44)	PIURA	40 (39 - 40)	AYABACA	773	0
2019	Coast	44 (44 - 44)	PIURA	40 (39 - 40)	HUANCABAMBA	757	0
2019	Coast	44 (44 - 44)	PIURA	40 (39 - 40)	MORROPON	2392	36 (34 - 38)
2019	Coast	44 (44 - 44)	PIURA	40 (39 - 40)	PAITA	1903	26 (24 - 28)
2019	Coast	44 (44 - 44)	PIURA	40 (39 - 40)	PIURA	11430	41 (41 - 42)
2019	Coast	44 (44 - 44)	PIURA	40 (39 - 40)	SECHURA	633	0
2019	Coast	44 (44 - 44)	PIURA	40 (39 - 40)	SULLANA	6599	57 (56 - 58)
2019	Coast	44 (44 - 44)	PIURA	40 (39 - 40)	TALARA	1253	28 (25 - 30)
2019	Coast	44 (44 - 44)	TACNA	47 (46 - 49)	TACNA	4846	48 (46 - 49)
2019	Coast	44 (44 - 44)	TUMBES	44 (42 - 45)	CONTRALMIRANTE VILLAR	124	0
2019	Coast	44 (44 - 44)	TUMBES	44 (42 - 45)	TUMBES	3676	49 (47 - 50)
2019	Coast	44 (44 - 44)	TUMBES	44 (42 - 45)	ZARUMILLA	282	0
2019	Highlands	31 (30 - 31)	ANCASH	35 (35 - 36)	ANTONIO RAIMONDI	70	0
2019	Highlands	31 (30 - 31)	ANCASH	35 (35 - 36)	ASUNCION	111	0
2019	Highlands	31 (30 - 31)	ANCASH	35 (35 - 36)	BOLOGNESI	107	0
2019	Highlands	31 (30 - 31)	ANCASH	35 (35 - 36)	CARHUAZ	485	8 (5 - 10)
2019	Highlands	31 (30 - 31)	ANCASH	35 (35 - 36)	CARLOS FERMIN FITZCARRALD	112	0

2019	Highlands	31 (30 - 31)	ANCASH	35 (35 - 36)	CASMA	480	15 (12 - 19)
2019	Highlands	31 (30 - 31)	ANCASH	35 (35 - 36)	HUARAZ	4090	38 (37 - 40)
2019	Highlands	31 (30 - 31)	ANCASH	35 (35 - 36)	HUARI	633	19 (16 - 22)
2019	Highlands	31 (30 - 31)	ANCASH	35 (35 - 36)	HUARMEY	210	19 (14 - 25)
2019	Highlands	31 (30 - 31)	ANCASH	35 (35 - 36)	HUAYLAS	716	25 (21 - 28)
2019	Highlands	31 (30 - 31)	ANCASH	35 (35 - 36)	MARISCAL LUZURIAGA	86	0
2019	Highlands	31 (30 - 31)	ANCASH	35 (35 - 36)	PALLASCA	115	0
2019	Highlands	31 (30 - 31)	ANCASH	35 (35 - 36)	POMABAMBA	359	18 (14 - 22)
2019	Highlands	31 (30 - 31)	ANCASH	35 (35 - 36)	RECUAY	124	23 (16 - 32)
2019	Highlands	31 (30 - 31)	ANCASH	35 (35 - 36)	SANTA	8326	46 (45 - 47)
2019	Highlands	31 (30 - 31)	ANCASH	35 (35 - 36)	SIHUAS	286	7 (5 - 11)
2019	Highlands	31 (30 - 31)	ANCASH	35 (35 - 36)	YUNGAY	455	0
2019	Highlands	31 (30 - 31)	APURIMAC	26 (25 - 27)	ABANCAY	2743	35 (33 - 37)
2019	Highlands	31 (30 - 31)	APURIMAC	26 (25 - 27)	ANDAHUAYLAS	2711	25 (23 - 26)
2019	Highlands	31 (30 - 31)	APURIMAC	26 (25 - 27)	ANTABAMBA	37	0
2019	Highlands	31 (30 - 31)	APURIMAC	26 (25 - 27)	AYMARAES	93	0
2019	Highlands	31 (30 - 31)	APURIMAC	26 (25 - 27)	CHINCHEROS	577	13 (11 - 16)
2019	Highlands	31 (30 - 31)	APURIMAC	26 (25 - 27)	COTABAMBAS	741	13 (10 - 15)
2019	Highlands	31 (30 - 31)	APURIMAC	26 (25 - 27)	GRAU	78	0
2019	Highlands	31 (30 - 31)	AREQUIPA	46 (45 - 47)	AREQUIPA	18460	50 (49 - 50)
2019	Highlands	31 (30 - 31)	AREQUIPA	46 (45 - 47)	CAMANA	1036	38 (35 - 41)
2019	Highlands	31 (30 - 31)	AREQUIPA	46 (45 - 47)	CARAVELI	121	0
2019	Highlands	31 (30 - 31)	AREQUIPA	46 (45 - 47)	CASTILLA	393	28 (24 - 33)
2019	Highlands	31 (30 - 31)	AREQUIPA	46 (45 - 47)	CAYLLOMA	1376	19 (17 - 22)
2019	Highlands	31 (30 - 31)	AREQUIPA	46 (45 - 47)	CONDESUYOS	76	0
2019	Highlands	31 (30 - 31)	AREQUIPA	46 (45 - 47)	ISLAY	354	39 (34 - 44)
2019	Highlands	31 (30 - 31)	AREQUIPA	46 (45 - 47)	LA UNION	65	0
2019	Highlands	31 (30 - 31)	AYACUCHO	25 (25 - 26)	CANGALLO	418	20 (16 - 24)
2019	Highlands	31 (30 - 31)	AYACUCHO	25 (25 - 26)	HUAMANGA	6815	32 (31 - 33)
2019	Highlands	31 (30 - 31)	AYACUCHO	25 (25 - 26)	HUANCA SANCOS	46	0
2019	Highlands	31 (30 - 31)	AYACUCHO	25 (25 - 26)	HUANTA	1595	16 (15 - 18)

2019	Highlands	31 (30 - 31)	AYACUCHO	25 (25 - 26)	LA MAR	1569	15 (13 - 17)
2019	Highlands	31 (30 - 31)	AYACUCHO	25 (25 - 26)	LUCANAS	437	19 (15 - 23)
2019	Highlands	31 (30 - 31)	AYACUCHO	25 (25 - 26)	PARINACOCHAS	426	21 (17 - 25)
2019	Highlands	31 (30 - 31)	AYACUCHO	25 (25 - 26)	PAUCAR DEL SARA SARA	46	0
2019	Highlands	31 (30 - 31)	AYACUCHO	25 (25 - 26)	SUCRE	72	0
2019	Highlands	31 (30 - 31)	AYACUCHO	25 (25 - 26)	VICTOR FAJARDO	48	0
2019	Highlands	31 (30 - 31)	AYACUCHO	25 (25 - 26)	VILCAS HUAMAN	88	0
2019	Highlands	31 (30 - 31)	CAJAMARCA	26 (25 - 26)	CAJABAMBA	1088	19 (16 - 21)
2019	Highlands	31 (30 - 31)	CAJAMARCA	26 (25 - 26)	CAJAMARCA	7311	26 (25 - 27)
2019	Highlands	31 (30 - 31)	CAJAMARCA	26 (25 - 26)	CELENDIN	609	3 (2 - 4)
2019	Highlands	31 (30 - 31)	CAJAMARCA	26 (25 - 26)	CHOTA	1817	30 (28 - 32)
2019	Highlands	31 (30 - 31)	CAJAMARCA	26 (25 - 26)	CONTUMAZA	126	0
2019	Highlands	31 (30 - 31)	CAJAMARCA	26 (25 - 26)	CUTERVO	1287	21 (19 - 23)
2019	Highlands	31 (30 - 31)	CAJAMARCA	26 (25 - 26)	HUALGAYOC	704	18 (15 - 21)
2019	Highlands	31 (30 - 31)	CAJAMARCA	26 (25 - 26)	JAEN	3587	43 (42 - 45)
2019	Highlands	31 (30 - 31)	CAJAMARCA	26 (25 - 26)	SAN IGNACIO	733	0
2019	Highlands	31 (30 - 31)	CAJAMARCA	26 (25 - 26)	SAN MARCOS	345	0
2019	Highlands	31 (30 - 31)	CAJAMARCA	26 (25 - 26)	SAN MIGUEL	150	0
2019	Highlands	31 (30 - 31)	CAJAMARCA	26 (25 - 26)	SAN PABLO	116	0
2019	Highlands	31 (30 - 31)	CAJAMARCA	26 (25 - 26)	SANTA CRUZ	154	1 (0 - 4)
2019	Highlands	31 (30 - 31)	CUSCO	31 (30 - 32)	ACOMAYO	90	0
2019	Highlands	31 (30 - 31)	CUSCO	31 (30 - 32)	ANTA	223	0
2019	Highlands	31 (30 - 31)	CUSCO	31 (30 - 32)	CALCA	521	0
2019	Highlands	31 (30 - 31)	CUSCO	31 (30 - 32)	CANAS	204	0
2019	Highlands	31 (30 - 31)	CUSCO	31 (30 - 32)	CANCHIS	1829	30 (28 - 32)
2019	Highlands	31 (30 - 31)	CUSCO	31 (30 - 32)	CHUMBIVILCAS	837	10 (8 - 12)
2019	Highlands	31 (30 - 31)	CUSCO	31 (30 - 32)	CUSCO	12196	41 (40 - 42)
2019	Highlands	31 (30 - 31)	CUSCO	31 (30 - 32)	ESPINAR	916	18 (15 - 20)
2019	Highlands	31 (30 - 31)	CUSCO	31 (30 - 32)	LA CONVENCION	2574	24 (22 - 25)
2019	Highlands	31 (30 - 31)	CUSCO	31 (30 - 32)	PARURO	39	0
2019	Highlands	31 (30 - 31)	CUSCO	31 (30 - 32)	PAUCARTAMBO	210	0

2019	Highlands	31 (30 - 31)	CUSCO	31 (30 - 32)	QUISPICANCHI	715	0
2019	Highlands	31 (30 - 31)	CUSCO	31 (30 - 32)	URUBAMBA	396	0
2019	Highlands	31 (30 - 31)	HUANCAVELICA	19 (18 - 20)	ACOBAMBA	595	11 (8 - 14)
2019	Highlands	31 (30 - 31)	HUANCAVELICA	19 (18 - 20)	ANGARAES	703	15 (13 - 18)
2019	Highlands	31 (30 - 31)	HUANCAVELICA	19 (18 - 20)	CASTROVIRREYNA	67	0
2019	Highlands	31 (30 - 31)	HUANCAVELICA	19 (18 - 20)	CHURCAMPA	383	0
2019	Highlands	31 (30 - 31)	HUANCAVELICA	19 (18 - 20)	HUANCAVELICA	2181	29 (28 - 31)
2019	Highlands	31 (30 - 31)	HUANCAVELICA	19 (18 - 20)	HUAYTARA	74	0
2019	Highlands	31 (30 - 31)	HUANCAVELICA	19 (18 - 20)	TAYACAJA	1043	12 (10 - 14)
2019	Highlands	31 (30 - 31)	HUANUCO	22 (22 - 23)	AMBO	615	0
2019	Highlands	31 (30 - 31)	HUANUCO	22 (22 - 23)	DOS DE MAYO	505	7 (5 - 9)
2019	Highlands	31 (30 - 31)	HUANUCO	22 (22 - 23)	HUACAYBAMBA	215	0
2019	Highlands	31 (30 - 31)	HUANUCO	22 (22 - 23)	HUAMALIES	670	8 (6 - 11)
2019	Highlands	31 (30 - 31)	HUANUCO	22 (22 - 23)	HUANUCO	6776	30 (29 - 32)
2019	Highlands	31 (30 - 31)	HUANUCO	22 (22 - 23)	LAURICOCHA	176	0
2019	Highlands	31 (30 - 31)	HUANUCO	22 (22 - 23)	LEONCIO PRADO	3023	30 (28 - 31)
2019	Highlands	31 (30 - 31)	HUANUCO	22 (22 - 23)	MARAÑON	202	0
2019	Highlands	31 (30 - 31)	HUANUCO	22 (22 - 23)	PACHITEA	887	0
2019	Highlands	31 (30 - 31)	HUANUCO	22 (22 - 23)	PUERTO INCA	441	0
2019	Highlands	31 (30 - 31)	HUANUCO	22 (22 - 23)	YAROWILCA	257	0
2019	Highlands	31 (30 - 31)	JUNIN	32 (32 - 33)	CHANCHAMAYO	2833	37 (36 - 39)
2019	Highlands	31 (30 - 31)	JUNIN	32 (32 - 33)	CHUPACA	437	0
2019	Highlands	31 (30 - 31)	JUNIN	32 (32 - 33)	CONCEPCION	330	0
2019	Highlands	31 (30 - 31)	JUNIN	32 (32 - 33)	HUANCAYO	10915	37 (36 - 38)
2019	Highlands	31 (30 - 31)	JUNIN	32 (32 - 33)	JAUJA	949	30 (27 - 33)
2019	Highlands	31 (30 - 31)	JUNIN	32 (32 - 33)	JUNIN	272	18 (14 - 24)
2019	Highlands	31 (30 - 31)	JUNIN	32 (32 - 33)	SATIPO	2690	28 (27 - 30)
2019	Highlands	31 (30 - 31)	JUNIN	32 (32 - 33)	TARMA	1309	16 (14 - 18)
2019	Highlands	31 (30 - 31)	JUNIN	32 (32 - 33)	YAULI	373	29 (24 - 34)
2019	Highlands	31 (30 - 31)	PASCO	22 (20 - 23)	DANIEL ALCIDES CARRION	191	0
2019	Highlands	31 (30 - 31)	PASCO	22 (20 - 23)	OXAPAMPA	1766	18 (16 - 20)

2019	Highlands	31 (30 - 31)	PASCO	22 (20 - 23)	PASCO	2218	27 (25 - 29)
2019	Highlands	31 (30 - 31)	PUNO	26 (26 - 27)	AZANGARO	1042	14 (12 - 16)
2019	Highlands	31 (30 - 31)	PUNO	26 (26 - 27)	CARABAYA	592	8 (6 - 11)
2019	Highlands	31 (30 - 31)	PUNO	26 (26 - 27)	CHUCUITO	681	7 (5 - 10)
2019	Highlands	31 (30 - 31)	PUNO	26 (26 - 27)	EL COLLAO	643	10 (8 - 13)
2019	Highlands	31 (30 - 31)	PUNO	26 (26 - 27)	HUANCANE	516	14 (11 - 17)
2019	Highlands	31 (30 - 31)	PUNO	26 (26 - 27)	LAMPA	259	7 (4 - 11)
2019	Highlands	31 (30 - 31)	PUNO	26 (26 - 27)	MELGAR	829	21 (18 - 24)
2019	Highlands	31 (30 - 31)	PUNO	26 (26 - 27)	MOHO	47	0
2019	Highlands	31 (30 - 31)	PUNO	26 (26 - 27)	PUNO	3250	32 (31 - 34)
2019	Highlands	31 (30 - 31)	PUNO	26 (26 - 27)	SAN ANTONIO DE PUTINA	165	0
2019	Highlands	31 (30 - 31)	PUNO	26 (26 - 27)	SAN ROMAN	6565	36 (34 - 37)
2019	Highlands	31 (30 - 31)	PUNO	26 (26 - 27)	SANDIA	431	10 (7 - 13)
2019	Highlands	31 (30 - 31)	PUNO	26 (26 - 27)	YUNGUYO	350	13 (10 - 17)
2020	Amazon	27 (27 - 28)	AMAZONAS	36 (35 - 38)	BAGUA	1152	46 (43 - 49)
2020	Amazon	27 (27 - 28)	AMAZONAS	36 (35 - 38)	BONGARA	199	0
2020	Amazon	27 (27 - 28)	AMAZONAS	36 (35 - 38)	CHACHAPOYAS	1603	44 (42 - 47)
2020	Amazon	27 (27 - 28)	AMAZONAS	36 (35 - 38)	CONDORCANQUI	341	20 (16 - 25)
2020	Amazon	27 (27 - 28)	AMAZONAS	36 (35 - 38)	LUYA	177	0
2020	Amazon	27 (27 - 28)	AMAZONAS	36 (35 - 38)	RODRIGUEZ DE MENDOZA	134	0
2020	Amazon	27 (27 - 28)	AMAZONAS	36 (35 - 38)	UTCUBAMBA	1139	36 (33 - 39)
2020	Amazon	27 (27 - 28)	LORETO	21 (20 - 22)	ALTO AMAZONAS	3318	17 (16 - 19)
2020	Amazon	27 (27 - 28)	LORETO	21 (20 - 22)	DATEM DEL MARAÑON	561	0
2020	Amazon	27 (27 - 28)	LORETO	21 (20 - 22)	LORETO	742	0
2020	Amazon	27 (27 - 28)	LORETO	21 (20 - 22)	MARISCAL RAMON CASTILLA	786	7 (6 - 9)
2020	Amazon	27 (27 - 28)	LORETO	21 (20 - 22)	MAYNAS	9846	29 (28 - 30)
2020	Amazon	27 (27 - 28)	LORETO	21 (20 - 22)	PUTUMAYO	63	0
2020	Amazon	27 (27 - 28)	LORETO	21 (20 - 22)	REQUENA	685	0
2020	Amazon	27 (27 - 28)	LORETO	21 (20 - 22)	UCAYALI	730	2 (1 - 3)
2020	Amazon	27 (27 - 28)	MADRE DE DIOS	28 (26 - 29)	MANU	143	0
2020	Amazon	27 (27 - 28)	MADRE DE DIOS	28 (26 - 29)	TAHUAMANU	136	3 (1 - 7)

2020	Amazon	27 (27 - 28)	MADRE DE DIOS	28 (26 - 29)	TAMBOPATA	3465	30 (28 - 31)
2020	Amazon	27 (27 - 28)	SAN MARTIN	32 (31 - 33)	BELLAVISTA	494	0
2020	Amazon	27 (27 - 28)	SAN MARTIN	32 (31 - 33)	EL DORADO	448	0
2020	Amazon	27 (27 - 28)	SAN MARTIN	32 (31 - 33)	HUALLAGA	147	0
2020	Amazon	27 (27 - 28)	SAN MARTIN	32 (31 - 33)	LAMAS	703	0
2020	Amazon	27 (27 - 28)	SAN MARTIN	32 (31 - 33)	MARISCAL CACERES	1054	39 (36 - 42)
2020	Amazon	27 (27 - 28)	SAN MARTIN	32 (31 - 33)	MOYOBAMBA	2162	40 (38 - 42)
2020	Amazon	27 (27 - 28)	SAN MARTIN	32 (31 - 33)	PICOTA	435	0
2020	Amazon	27 (27 - 28)	SAN MARTIN	32 (31 - 33)	RIOJA	2110	29 (27 - 31)
2020	Amazon	27 (27 - 28)	SAN MARTIN	32 (31 - 33)	SAN MARTIN	4766	45 (44 - 47)
2020	Amazon	27 (27 - 28)	SAN MARTIN	32 (31 - 33)	TOCACHE	1348	26 (24 - 29)
2020	Amazon	27 (27 - 28)	UCAYALI	27 (26 - 27)	ATALAYA	763	0
2020	Amazon	27 (27 - 28)	UCAYALI	27 (26 - 27)	CORONEL PORTILLO	9952	32 (31 - 33)
2020	Amazon	27 (27 - 28)	UCAYALI	27 (26 - 27)	PADRE ABAD	1120	0
2020	Amazon	27 (27 - 28)	UCAYALI	27 (26 - 27)	PURUS	35	0
2020	Coast	45 (44 - 45)	CALLAO	42 (41 - 42)	CALLAO	17676	42 (41 - 42)
2020	Coast	45 (44 - 45)	ICA	41 (41 - 42)	CHINCHA	4191	34 (33 - 36)
2020	Coast	45 (44 - 45)	ICA	41 (41 - 42)	ICA	7938	47 (46 - 48)
2020	Coast	45 (44 - 45)	ICA	41 (41 - 42)	NAZCA	1159	34 (32 - 37)
2020	Coast	45 (44 - 45)	ICA	41 (41 - 42)	PALPA	94	0
2020	Coast	45 (44 - 45)	ICA	41 (41 - 42)	PISCO	2759	40 (38 - 42)
2020	Coast	45 (44 - 45)	LA LIBERTAD	39 (39 - 40)	ASCOPE	968	29 (26 - 32)
2020	Coast	45 (44 - 45)	LA LIBERTAD	39 (39 - 40)	BOLIVAR	135	0
2020	Coast	45 (44 - 45)	LA LIBERTAD	39 (39 - 40)	CHEPEN	973	38 (35 - 41)
2020	Coast	45 (44 - 45)	LA LIBERTAD	39 (39 - 40)	GRAN CHIMU	197	0
2020	Coast	45 (44 - 45)	LA LIBERTAD	39 (39 - 40)	JULCAN	295	0
2020	Coast	45 (44 - 45)	LA LIBERTAD	39 (39 - 40)	OTUZCO	578	29 (25 - 33)
2020	Coast	45 (44 - 45)	LA LIBERTAD	39 (39 - 40)	PACASMAYO	872	33 (30 - 36)
2020	Coast	45 (44 - 45)	LA LIBERTAD	39 (39 - 40)	PATAZ	712	0
2020	Coast	45 (44 - 45)	LA LIBERTAD	39 (39 - 40)	SANCHEZ CARRION	2080	20 (19 - 22)
2020	Coast	45 (44 - 45)	LA LIBERTAD	39 (39 - 40)	SANTIAGO DE CHUCO	533	22 (19 - 26)

2020	Coast	45 (44 - 45)	LA LIBERTAD	39 (39 - 40)	TRUJILLO	15363	50 (49 - 51)
2020	Coast	45 (44 - 45)	LA LIBERTAD	39 (39 - 40)	VIRU	1206	0
2020	Coast	45 (44 - 45)	LAMBAYEQUE	34 (33 - 34)	CHICLAYO	10905	39 (38 - 40)
2020	Coast	45 (44 - 45)	LAMBAYEQUE	34 (33 - 34)	FERREÑAFE	536	1 (0 - 2)
2020	Coast	45 (44 - 45)	LAMBAYEQUE	34 (33 - 34)	LAMBAYEQUE	3792	22 (21 - 24)
2020	Coast	45 (44 - 45)	LIMA	48 (47 - 48)	BARRANCA	1926	39 (37 - 41)
2020	Coast	45 (44 - 45)	LIMA	48 (47 - 48)	CAÑETE	3969	36 (35 - 38)
2020	Coast	45 (44 - 45)	LIMA	48 (47 - 48)	HUARAL	2594	34 (32 - 35)
2020	Coast	45 (44 - 45)	LIMA	48 (47 - 48)	HUAROCHIRI	258	1 (0 - 3)
2020	Coast	45 (44 - 45)	LIMA	48 (47 - 48)	HUAURA	4390	45 (43 - 46)
2020	Coast	45 (44 - 45)	LIMA	48 (47 - 48)	LIMA	133677	48 (48 - 49)
2020	Coast	45 (44 - 45)	MOQUEGUA	44 (42 - 46)	GENERAL SANCHEZ CERRO	41	0
2020	Coast	45 (44 - 45)	MOQUEGUA	44 (42 - 46)	ILO	1032	44 (41 - 47)
2020	Coast	45 (44 - 45)	MOQUEGUA	44 (42 - 46)	MARISCAL NIETO	1198	45 (42 - 48)
2020	Coast	45 (44 - 45)	PIURA	43 (43 - 44)	AYABACA	889	0
2020	Coast	45 (44 - 45)	PIURA	43 (43 - 44)	HUANCABAMBA	932	2 (1 - 3)
2020	Coast	45 (44 - 45)	PIURA	43 (43 - 44)	MORROPON	2195	32 (30 - 34)
2020	Coast	45 (44 - 45)	PIURA	43 (43 - 44)	PAITA	1664	27 (24 - 29)
2020	Coast	45 (44 - 45)	PIURA	43 (43 - 44)	PIURA	10264	48 (47 - 49)
2020	Coast	45 (44 - 45)	PIURA	43 (43 - 44)	SECHURA	517	1 (0 - 2)
2020	Coast	45 (44 - 45)	PIURA	43 (43 - 44)	SULLANA	6084	64 (63 - 66)
2020	Coast	45 (44 - 45)	PIURA	43 (43 - 44)	TALARA	1159	24 (22 - 27)
2020	Coast	45 (44 - 45)	TACNA	48 (47 - 49)	TACNA	4390	48 (47 - 50)
2020	Coast	45 (44 - 45)	TUMBES	42 (40 - 43)	CONTRALMIRANTE VILLAR	130	0
2020	Coast	45 (44 - 45)	TUMBES	42 (40 - 43)	TUMBES	3466	49 (47 - 50)
2020	Coast	45 (44 - 45)	TUMBES	42 (40 - 43)	ZARUMILLA	448	0
2020	Highlands	31 (30 - 31)	ANCASH	37 (37 - 38)	ANTONIO RAIMONDI	62	0
2020	Highlands	31 (30 - 31)	ANCASH	37 (37 - 38)	ASUNCION	84	0
2020	Highlands	31 (30 - 31)	ANCASH	37 (37 - 38)	BOLOGNESI	151	0
2020	Highlands	31 (30 - 31)	ANCASH	37 (37 - 38)	CARHUAZ	642	10 (8 - 13)
2020	Highlands	31 (30 - 31)	ANCASH	37 (37 - 38)	CARLOS FERMIN FITZCARRALD	126	0

2020	Highlands	31 (30 - 31)	ANCASH	37 (37 - 38)	CASMA	476	11 (8 - 14)
2020	Highlands	31 (30 - 31)	ANCASH	37 (37 - 38)	HUARAZ	3694	39 (37 - 40)
2020	Highlands	31 (30 - 31)	ANCASH	37 (37 - 38)	HUARI	735	24 (21 - 28)
2020	Highlands	31 (30 - 31)	ANCASH	37 (37 - 38)	HUARMEY	284	32 (26 - 37)
2020	Highlands	31 (30 - 31)	ANCASH	37 (37 - 38)	HUAYLAS	866	32 (29 - 35)
2020	Highlands	31 (30 - 31)	ANCASH	37 (37 - 38)	MARISCAL LUZURIAGA	62	0
2020	Highlands	31 (30 - 31)	ANCASH	37 (37 - 38)	PALLASCA	135	0
2020	Highlands	31 (30 - 31)	ANCASH	37 (37 - 38)	POMABAMBA	359	27 (23 - 32)
2020	Highlands	31 (30 - 31)	ANCASH	37 (37 - 38)	RECUAY	129	2 (0 - 7)
2020	Highlands	31 (30 - 31)	ANCASH	37 (37 - 38)	SANTA	7906	49 (48 - 50)
2020	Highlands	31 (30 - 31)	ANCASH	37 (37 - 38)	SIHUAS	301	26 (21 - 31)
2020	Highlands	31 (30 - 31)	ANCASH	37 (37 - 38)	YUNGAY	498	0
2020	Highlands	31 (30 - 31)	APURIMAC	25 (24 - 26)	ABANCAY	2659	33 (31 - 34)
2020	Highlands	31 (30 - 31)	APURIMAC	25 (24 - 26)	ANDAHUAYLAS	2728	26 (24 - 28)
2020	Highlands	31 (30 - 31)	APURIMAC	25 (24 - 26)	ANTABAMBA	51	0
2020	Highlands	31 (30 - 31)	APURIMAC	25 (24 - 26)	AYMARAES	160	0
2020	Highlands	31 (30 - 31)	APURIMAC	25 (24 - 26)	CHINCHEROS	652	14 (11 - 17)
2020	Highlands	31 (30 - 31)	APURIMAC	25 (24 - 26)	COTABAMBAS	911	18 (15 - 20)
2020	Highlands	31 (30 - 31)	APURIMAC	25 (24 - 26)	GRAU	142	0
2020	Highlands	31 (30 - 31)	AREQUIPA	47 (46 - 47)	AREQUIPA	17492	52 (51 - 52)
2020	Highlands	31 (30 - 31)	AREQUIPA	47 (46 - 47)	CAMANA	1000	34 (31 - 37)
2020	Highlands	31 (30 - 31)	AREQUIPA	47 (46 - 47)	CARAVELI	198	0
2020	Highlands	31 (30 - 31)	AREQUIPA	47 (46 - 47)	CASTILLA	468	29 (25 - 33)
2020	Highlands	31 (30 - 31)	AREQUIPA	47 (46 - 47)	CAYLLOMA	1492	19 (17 - 21)
2020	Highlands	31 (30 - 31)	AREQUIPA	47 (46 - 47)	CONDESUYOS	105	0
2020	Highlands	31 (30 - 31)	AREQUIPA	47 (46 - 47)	ISLAY	333	36 (31 - 41)
2020	Highlands	31 (30 - 31)	AREQUIPA	47 (46 - 47)	LA UNION	94	0
2020	Highlands	31 (30 - 31)	AYACUCHO	26 (25 - 26)	CANGALLO	500	17 (14 - 21)
2020	Highlands	31 (30 - 31)	AYACUCHO	26 (25 - 26)	HUAMANGA	6380	33 (32 - 35)
2020	Highlands	31 (30 - 31)	AYACUCHO	26 (25 - 26)	HUANCA SANCOS	58	0
2020	Highlands	31 (30 - 31)	AYACUCHO	26 (25 - 26)	HUANTA	1700	13 (12 - 15)

2020	Highlands	31 (30 - 31)	AYACUCHO	26 (25 - 26)	LA MAR	1826	20 (18 - 22)
2020	Highlands	31 (30 - 31)	AYACUCHO	26 (25 - 26)	LUCANAS	542	22 (19 - 26)
2020	Highlands	31 (30 - 31)	AYACUCHO	26 (25 - 26)	PARINACOCHAS	469	22 (18 - 26)
2020	Highlands	31 (30 - 31)	AYACUCHO	26 (25 - 26)	PAUCAR DEL SARA SARA	63	0
2020	Highlands	31 (30 - 31)	AYACUCHO	26 (25 - 26)	SUCRE	78	0
2020	Highlands	31 (30 - 31)	AYACUCHO	26 (25 - 26)	VICTOR FAJARDO	55	0
2020	Highlands	31 (30 - 31)	AYACUCHO	26 (25 - 26)	VILCAS HUAMAN	134	0
2020	Highlands	31 (30 - 31)	CAJAMARCA	26 (25 - 26)	CAJABAMBA	1182	21 (18 - 23)
2020	Highlands	31 (30 - 31)	CAJAMARCA	26 (25 - 26)	CAJAMARCA	6524	28 (27 - 29)
2020	Highlands	31 (30 - 31)	CAJAMARCA	26 (25 - 26)	CELENDIN	627	4 (3 - 6)
2020	Highlands	31 (30 - 31)	CAJAMARCA	26 (25 - 26)	CHOTA	1700	28 (25 - 30)
2020	Highlands	31 (30 - 31)	CAJAMARCA	26 (25 - 26)	CONTUMAZA	151	0
2020	Highlands	31 (30 - 31)	CAJAMARCA	26 (25 - 26)	CUTERVO	1317	26 (24 - 29)
2020	Highlands	31 (30 - 31)	CAJAMARCA	26 (25 - 26)	HUALGAYOC	755	23 (20 - 26)
2020	Highlands	31 (30 - 31)	CAJAMARCA	26 (25 - 26)	JAEN	3263	44 (42 - 45)
2020	Highlands	31 (30 - 31)	CAJAMARCA	26 (25 - 26)	SAN IGNACIO	893	0
2020	Highlands	31 (30 - 31)	CAJAMARCA	26 (25 - 26)	SAN MARCOS	446	0
2020	Highlands	31 (30 - 31)	CAJAMARCA	26 (25 - 26)	SAN MIGUEL	189	0
2020	Highlands	31 (30 - 31)	CAJAMARCA	26 (25 - 26)	SAN PABLO	133	0
2020	Highlands	31 (30 - 31)	CAJAMARCA	26 (25 - 26)	SANTA CRUZ	265	2 (0 - 4)
2020	Highlands	31 (30 - 31)	CUSCO	29 (28 - 30)	ACOMAYO	116	0
2020	Highlands	31 (30 - 31)	CUSCO	29 (28 - 30)	ANTA	400	0
2020	Highlands	31 (30 - 31)	CUSCO	29 (28 - 30)	CALCA	593	0
2020	Highlands	31 (30 - 31)	CUSCO	29 (28 - 30)	CANAS	265	0
2020	Highlands	31 (30 - 31)	CUSCO	29 (28 - 30)	CANCHIS	1793	25 (23 - 28)
2020	Highlands	31 (30 - 31)	CUSCO	29 (28 - 30)	CHUMBIVILCAS	1071	8 (7 - 10)
2020	Highlands	31 (30 - 31)	CUSCO	29 (28 - 30)	CUSCO	10963	42 (41 - 43)
2020	Highlands	31 (30 - 31)	CUSCO	29 (28 - 30)	ESPINAR	968	19 (17 - 22)
2020	Highlands	31 (30 - 31)	CUSCO	29 (28 - 30)	LA CONVENCION	2767	20 (19 - 22)
2020	Highlands	31 (30 - 31)	CUSCO	29 (28 - 30)	PARURO	79	0
2020	Highlands	31 (30 - 31)	CUSCO	29 (28 - 30)	PAUCARTAMBO	270	0

2020	Highlands	31 (30 - 31)	CUSCO	29 (28 - 30)	QUISPICANCHI	771	0
2020	Highlands	31 (30 - 31)	CUSCO	29 (28 - 30)	URUBAMBA	462	0
2020	Highlands	31 (30 - 31)	HUANCAVELICA	19 (18 - 20)	ACOBAMBA	658	15 (12 - 18)
2020	Highlands	31 (30 - 31)	HUANCAVELICA	19 (18 - 20)	ANGARAES	708	13 (10 - 15)
2020	Highlands	31 (30 - 31)	HUANCAVELICA	19 (18 - 20)	CASTROVIRREYNA	101	0
2020	Highlands	31 (30 - 31)	HUANCAVELICA	19 (18 - 20)	CHURCAMPA	448	0
2020	Highlands	31 (30 - 31)	HUANCAVELICA	19 (18 - 20)	HUANCAVELICA	2386	27 (26 - 29)
2020	Highlands	31 (30 - 31)	HUANCAVELICA	19 (18 - 20)	HUAYTARA	80	0
2020	Highlands	31 (30 - 31)	HUANCAVELICA	19 (18 - 20)	TAYACAJA	1357	16 (14 - 18)
2020	Highlands	31 (30 - 31)	HUANUCO	20 (19 - 21)	AMBO	755	0
2020	Highlands	31 (30 - 31)	HUANUCO	20 (19 - 21)	DOS DE MAYO	503	7 (5 - 9)
2020	Highlands	31 (30 - 31)	HUANUCO	20 (19 - 21)	HUACAYBAMBA	244	0
2020	Highlands	31 (30 - 31)	HUANUCO	20 (19 - 21)	HUAMALIES	793	11 (9 - 13)
2020	Highlands	31 (30 - 31)	HUANUCO	20 (19 - 21)	HUANUCO	6394	29 (28 - 31)
2020	Highlands	31 (30 - 31)	HUANUCO	20 (19 - 21)	LAURICOCHA	223	0
2020	Highlands	31 (30 - 31)	HUANUCO	20 (19 - 21)	LEONCIO PRADO	2916	26 (24 - 27)
2020	Highlands	31 (30 - 31)	HUANUCO	20 (19 - 21)	MARAÑON	213	0
2020	Highlands	31 (30 - 31)	HUANUCO	20 (19 - 21)	PACHITEA	974	0
2020	Highlands	31 (30 - 31)	HUANUCO	20 (19 - 21)	PUERTO INCA	502	0
2020	Highlands	31 (30 - 31)	HUANUCO	20 (19 - 21)	YAROWILCA	325	0
2020	Highlands	31 (30 - 31)	JUNIN	35 (34 - 36)	CHANCHAMAYO	2816	41 (39 - 43)
2020	Highlands	31 (30 - 31)	JUNIN	35 (34 - 36)	CHUPACA	529	0
2020	Highlands	31 (30 - 31)	JUNIN	35 (34 - 36)	CONCEPCION	512	0
2020	Highlands	31 (30 - 31)	JUNIN	35 (34 - 36)	HUANCAYO	10093	43 (42 - 44)
2020	Highlands	31 (30 - 31)	JUNIN	35 (34 - 36)	JAUJA	1061	29 (26 - 32)
2020	Highlands	31 (30 - 31)	JUNIN	35 (34 - 36)	JUNIN	321	16 (12 - 20)
2020	Highlands	31 (30 - 31)	JUNIN	35 (34 - 36)	SATIPO	2620	26 (24 - 27)
2020	Highlands	31 (30 - 31)	JUNIN	35 (34 - 36)	TARMA	1277	19 (17 - 22)
2020	Highlands	31 (30 - 31)	JUNIN	35 (34 - 36)	YAULI	337	23 (18 - 28)
2020	Highlands	31 (30 - 31)	PASCO	22 (21 - 23)	DANIEL ALCIDES CARRION	220	0
2020	Highlands	31 (30 - 31)	PASCO	22 (21 - 23)	OXAPAMPA	1740	18 (16 - 20)

2020	Highlands	31 (30 - 31)	PASCO	22 (21 - 23)	PASCO	2232	27 (25 - 29)
2020	Highlands	31 (30 - 31)	PUNO	26 (25 - 26)	AZANGARO	1218	16 (14 - 18)
2020	Highlands	31 (30 - 31)	PUNO	26 (25 - 26)	CARABAYA	825	19 (16 - 21)
2020	Highlands	31 (30 - 31)	PUNO	26 (25 - 26)	CHUCUITO	758	9 (7 - 12)
2020	Highlands	31 (30 - 31)	PUNO	26 (25 - 26)	EL COLLAO	680	15 (12 - 18)
2020	Highlands	31 (30 - 31)	PUNO	26 (25 - 26)	HUANCANE	657	18 (15 - 21)
2020	Highlands	31 (30 - 31)	PUNO	26 (25 - 26)	LAMPA	322	9 (6 - 12)
2020	Highlands	31 (30 - 31)	PUNO	26 (25 - 26)	MELGAR	943	25 (22 - 27)
2020	Highlands	31 (30 - 31)	PUNO	26 (25 - 26)	MOHO	109	0
2020	Highlands	31 (30 - 31)	PUNO	26 (25 - 26)	PUNO	2925	30 (28 - 32)
2020	Highlands	31 (30 - 31)	PUNO	26 (25 - 26)	SAN ANTONIO DE PUTINA	207	0
2020	Highlands	31 (30 - 31)	PUNO	26 (25 - 26)	SAN ROMAN	5646	36 (34 - 37)
2020	Highlands	31 (30 - 31)	PUNO	26 (25 - 26)	SANDIA	641	11 (9 - 14)
2020	Highlands	31 (30 - 31)	PUNO	26 (25 - 26)	YUNGUYO	347	15 (11 - 19)

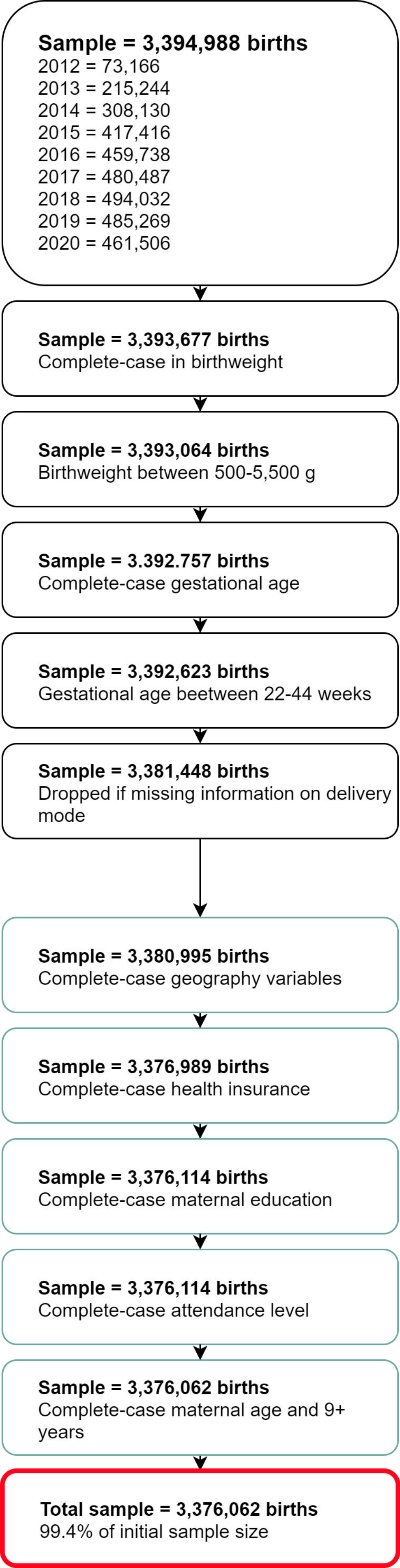
Sample = 3,394,988 births
2012 = 73,166
2013 = 215,244
2014 = 308,130
2015 = 417,416
2016 = 459,738
2017 = 480,487
2018 = 494,032
2019 = 485,269
2020 = 461,506
Sample = 3,393,677 births
Complete-case in birthweight
Sample = 3,393,064 births
Birthweight between 500-5,500 g
Sample = 3.392.757 births
Complete-case gestational age
Sample = 3,392,623 births
Gestational age beetween 22-44 weeks
Sample = 3,381,448 births
Dropped if missing information on delivery mode
Sample = 3,380,995 births
Complete-case geography variables
Sample = 3,376,989 births
Complete-case health insurance
Sample = 3,376,114 births
Complete-case maternal education
Sample = 3,376,114 births
Complete-case attendance level
Sample = 3,376,062 births
Complete-case maternal age and 9+ years
Total sample = 3,376,062 births
99.4% of initial sample size

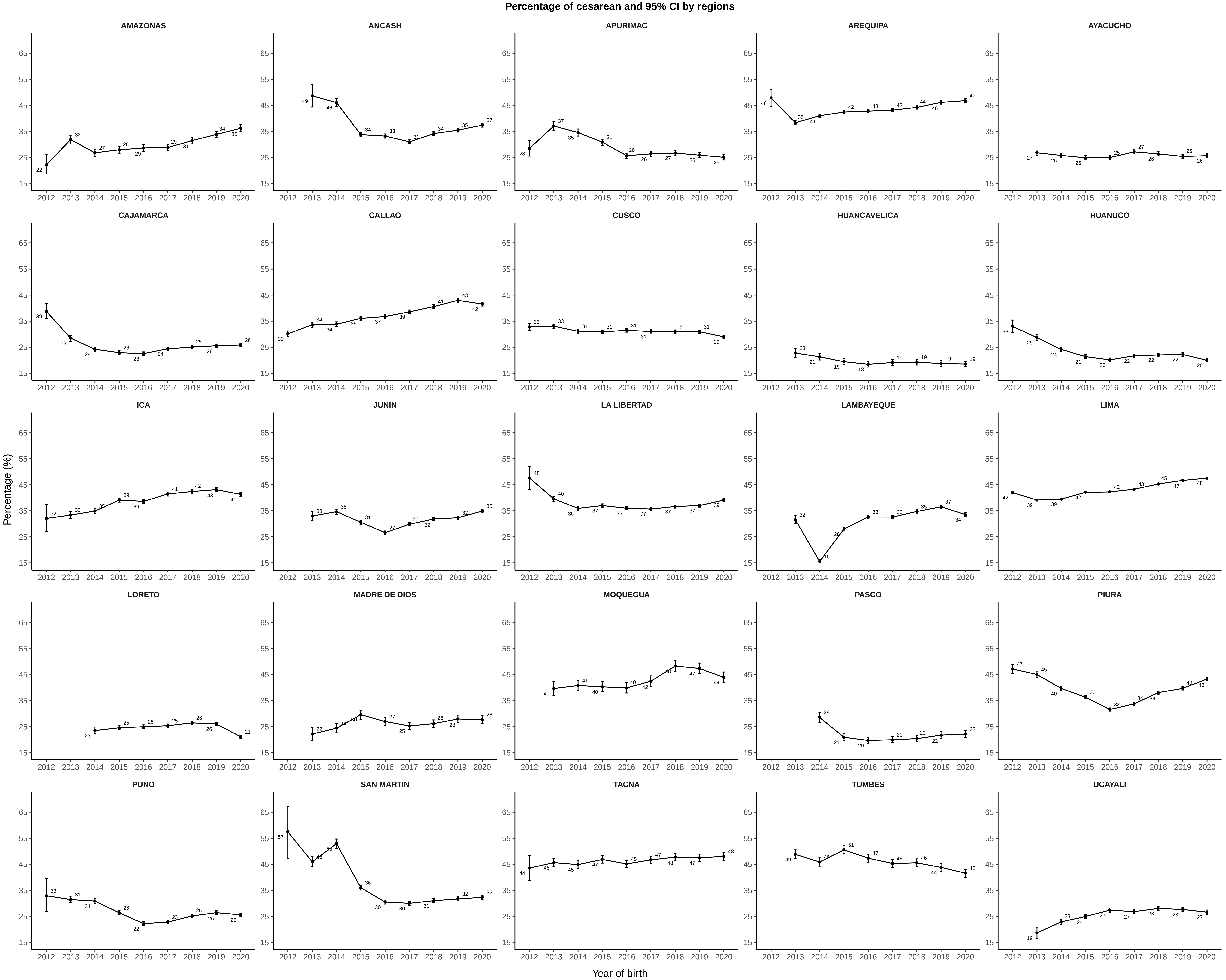

Percentage of cesarean and 95% CI by regions
AMAZONAS
ANCASH
APURIMAC
AREQUIPA
AYACUCHO
CAJAMARCA
CALLAO
CUSCO
HUANCAVELICA
HUANUCO
ICA
JUNIN
LA LIBERTAD
LAMBAYEQUE
LIMA
LORETO
MADRE DE DIOS
MOQUEGUA
PASCO
PIURA
PUNO
SAN MARTIN
TACNA
TUMBES
UCAYALI
Percentage (%)
Year of birth

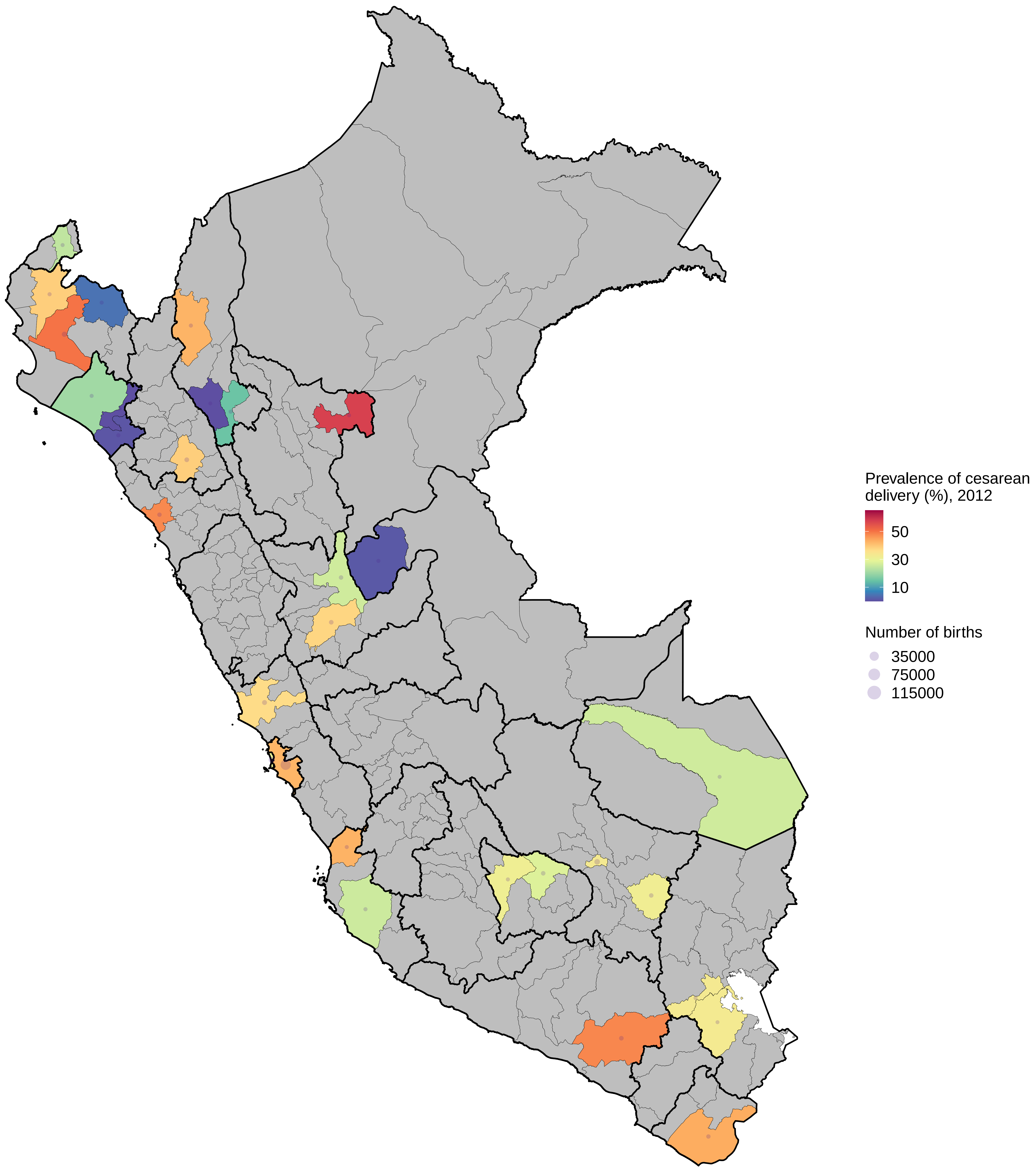

Prevalence of cesarean delivery (%), 2012
50
30
10
Number of births
35000
75000
115000

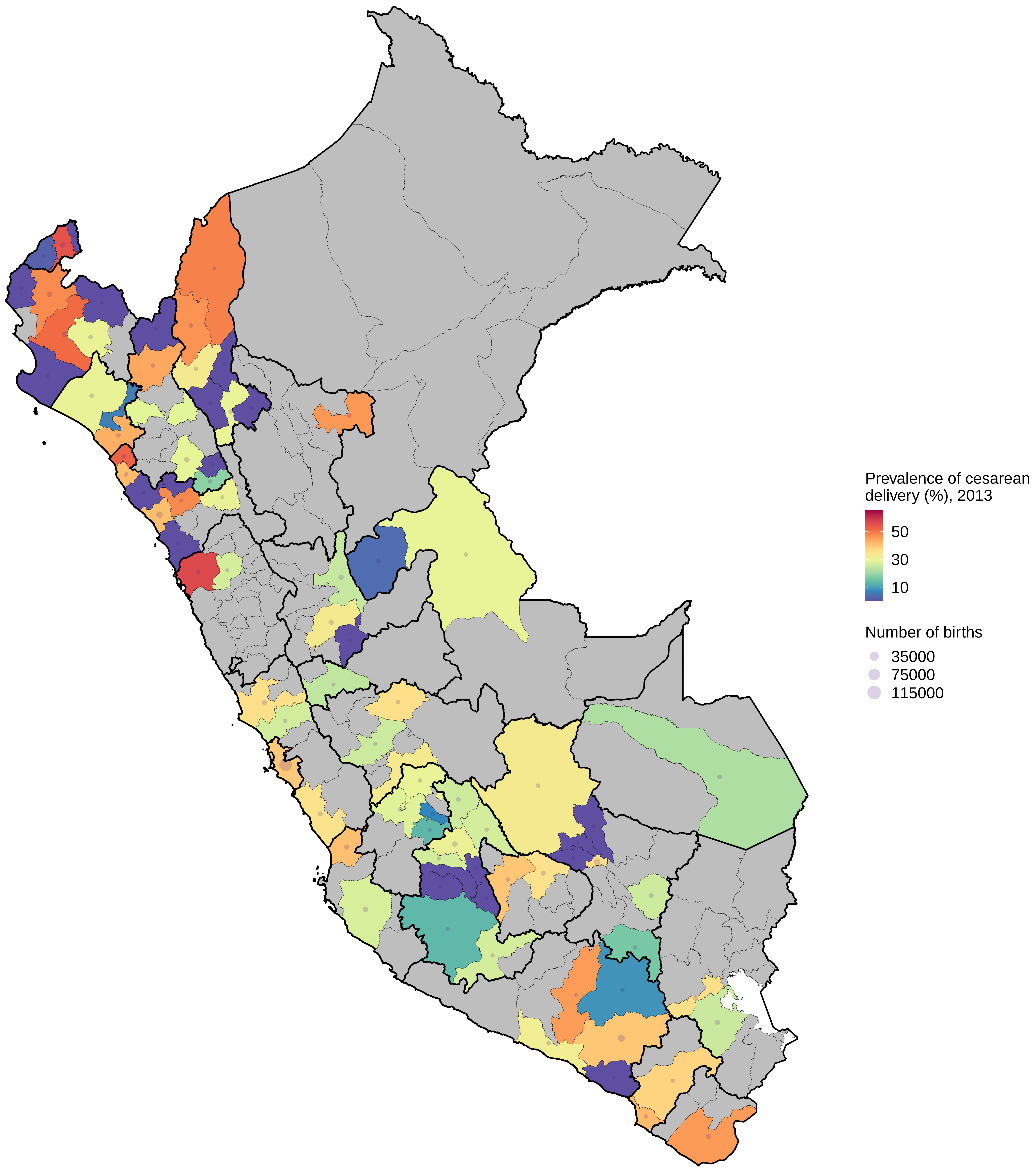

Prevalence of cesarean delivery (%), 2013
50
30
10
Number of births
35000
75000
115000

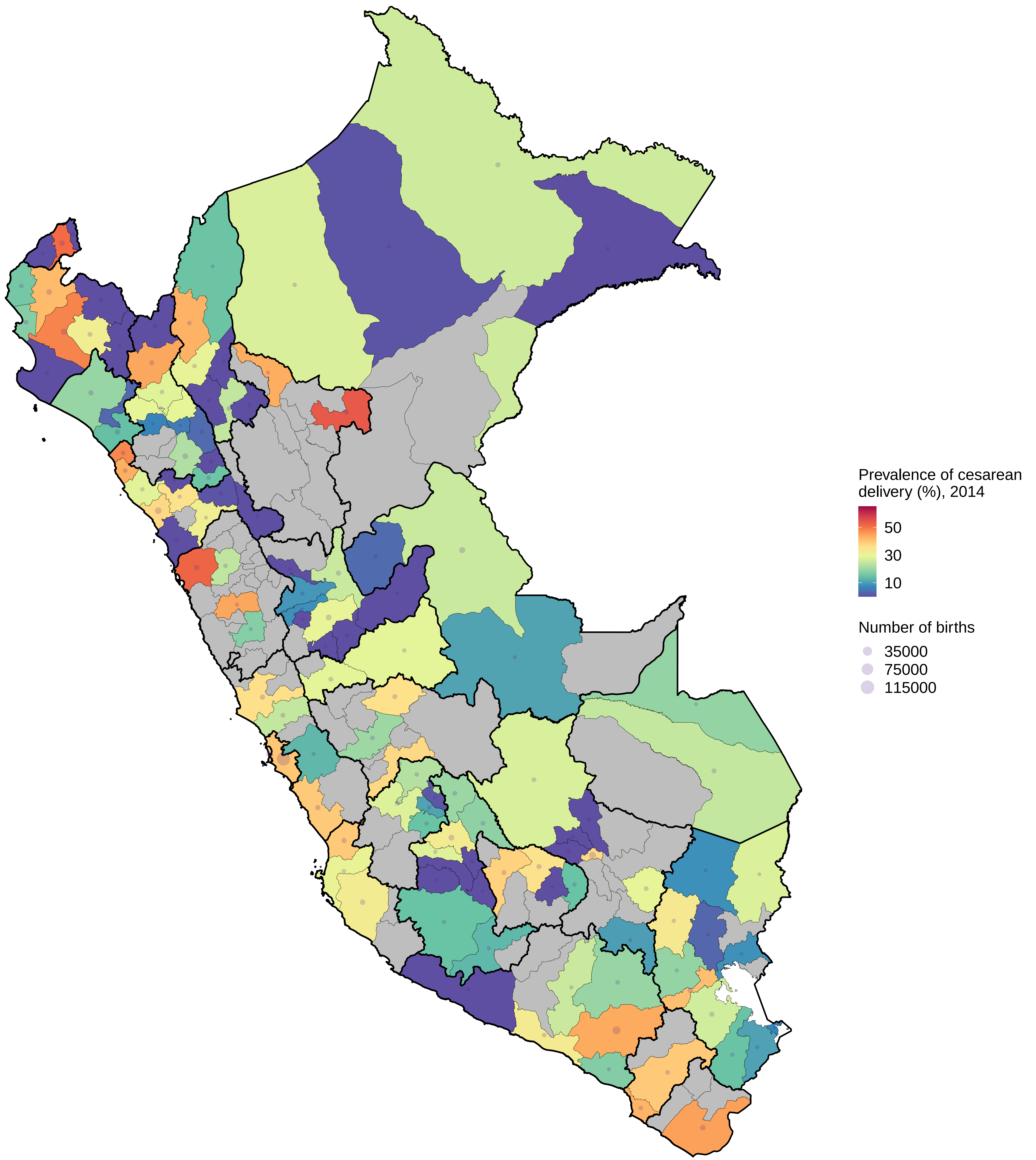
Prevalence of cesarean delivery (%), 2014
50
30
10
Number of births
35000
75000
115000

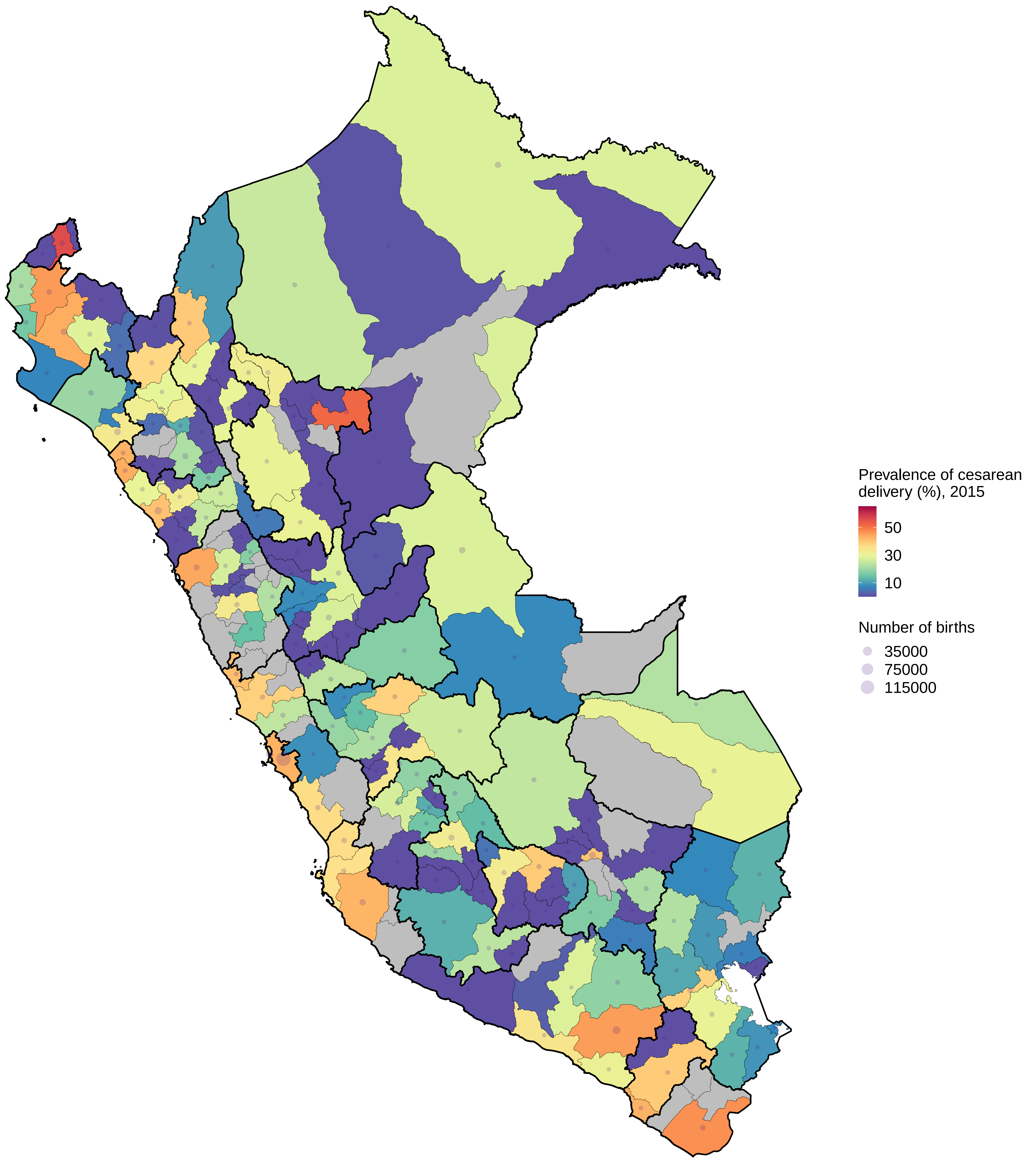
Prevalence of cesarean delivery (%), 2015
50
30
10
Number of births
35000
75000
115000

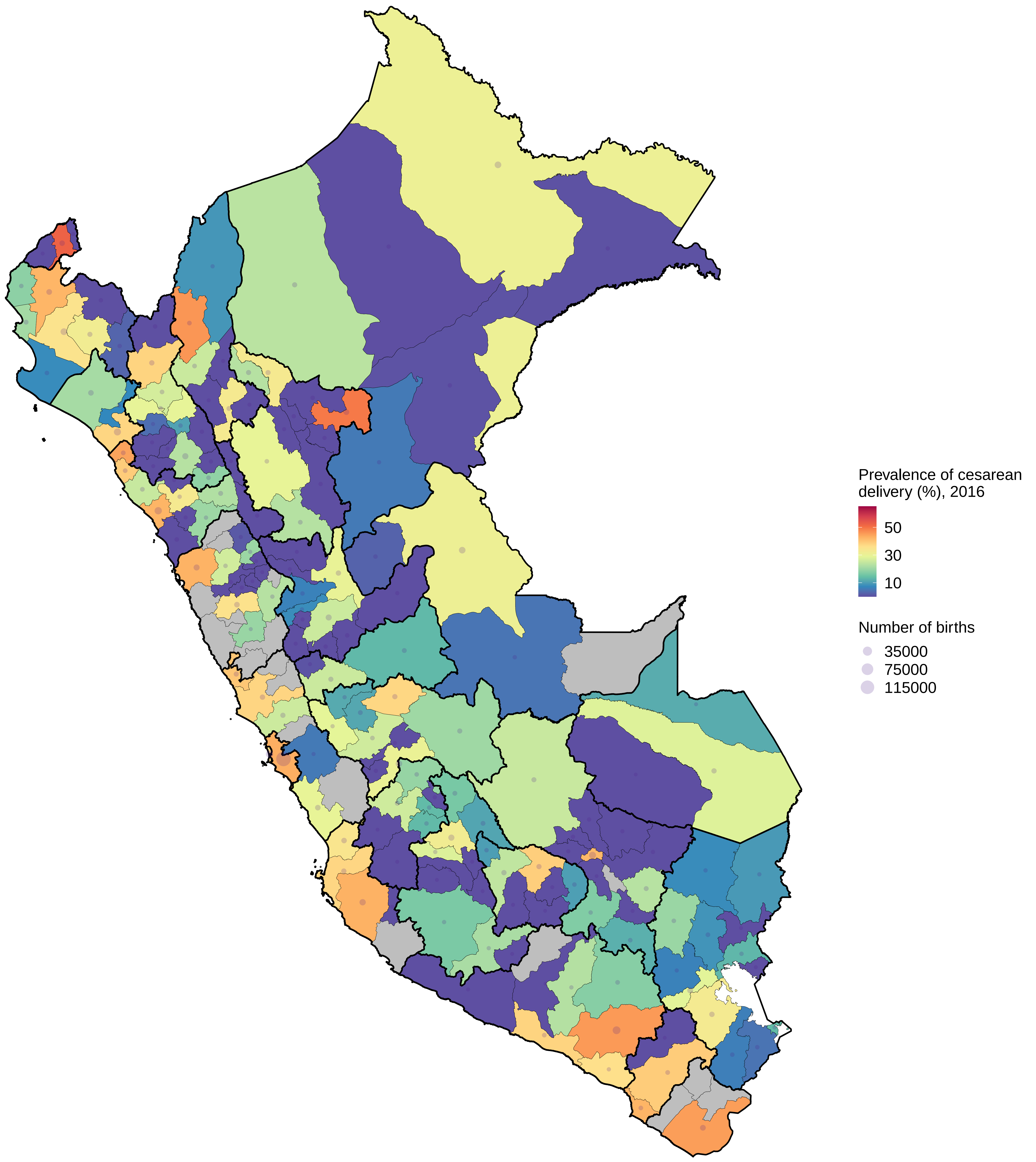
Prevalence of cesarean delivery (%), 2016
50
30
10
Number of births
35000
75000
115000

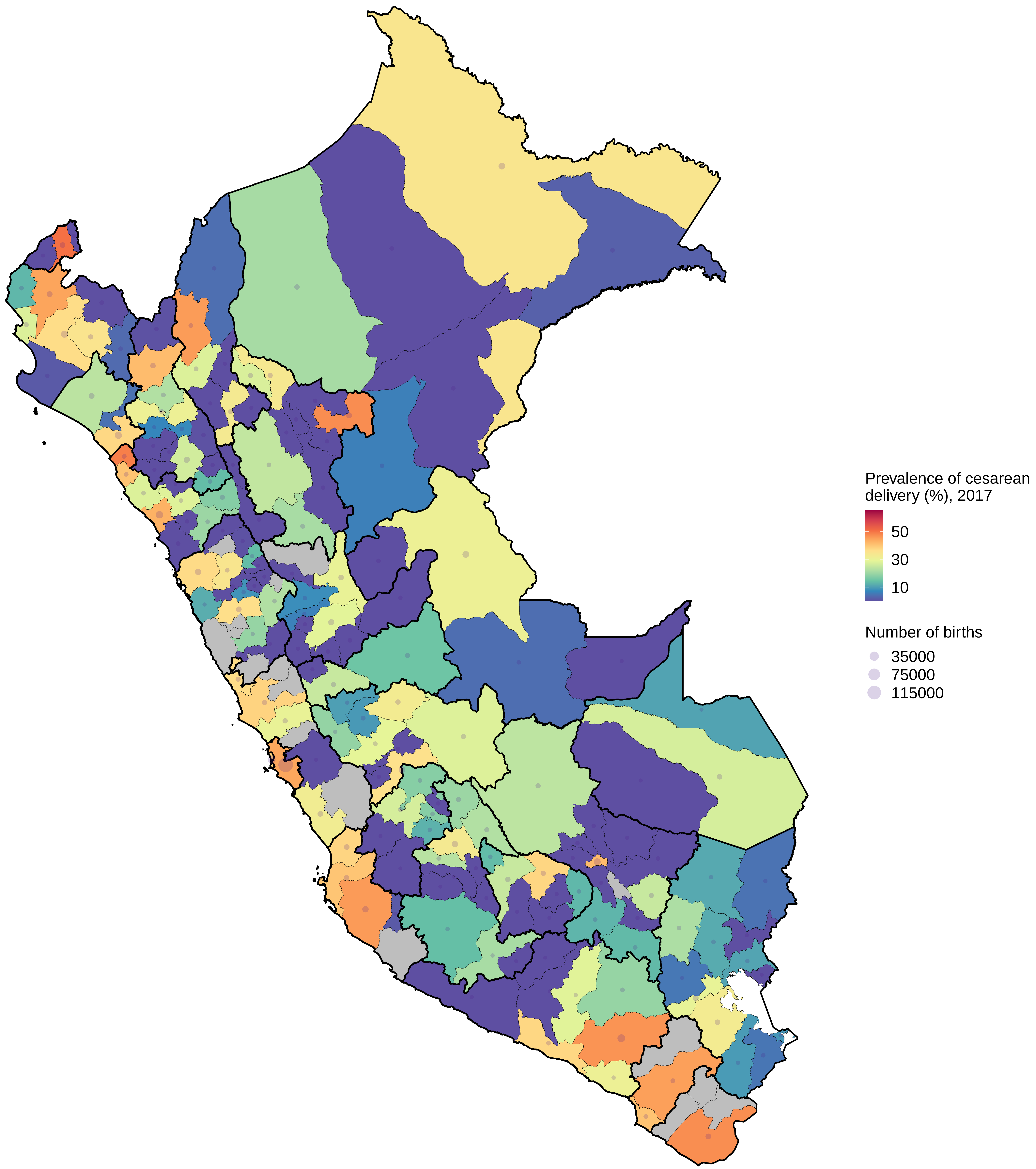

Prevalence of cesarean delivery (%), 2017
50
30
10
Number of births
35000
75000
115000

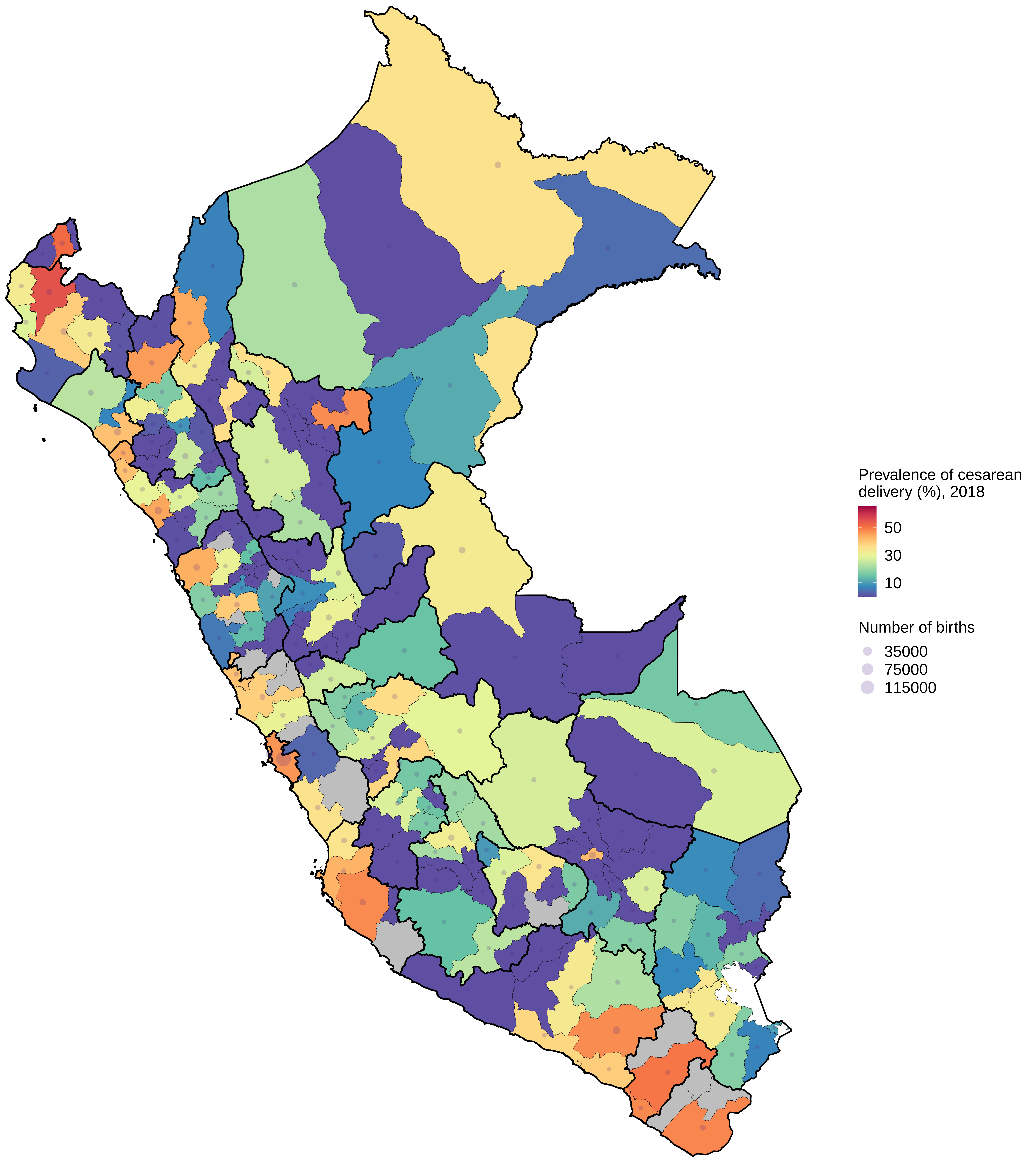

Prevalence of cesarean delivery (%), 2018
50
30
10
Number of births
35000
75000
115000

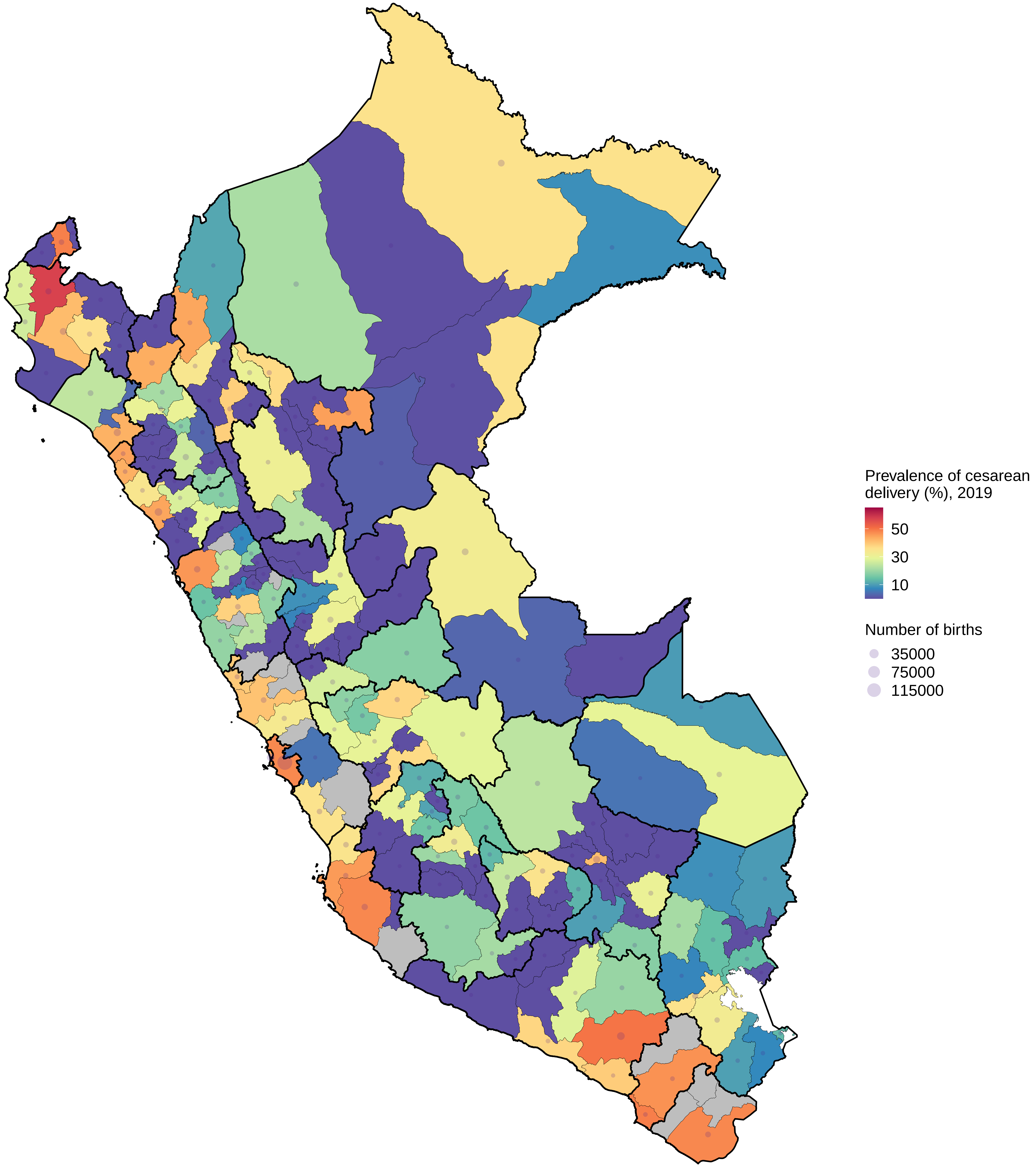
Prevalence of cesarean
delivery (%), 2019
50
30
10
Number of births
35000
75000
115000

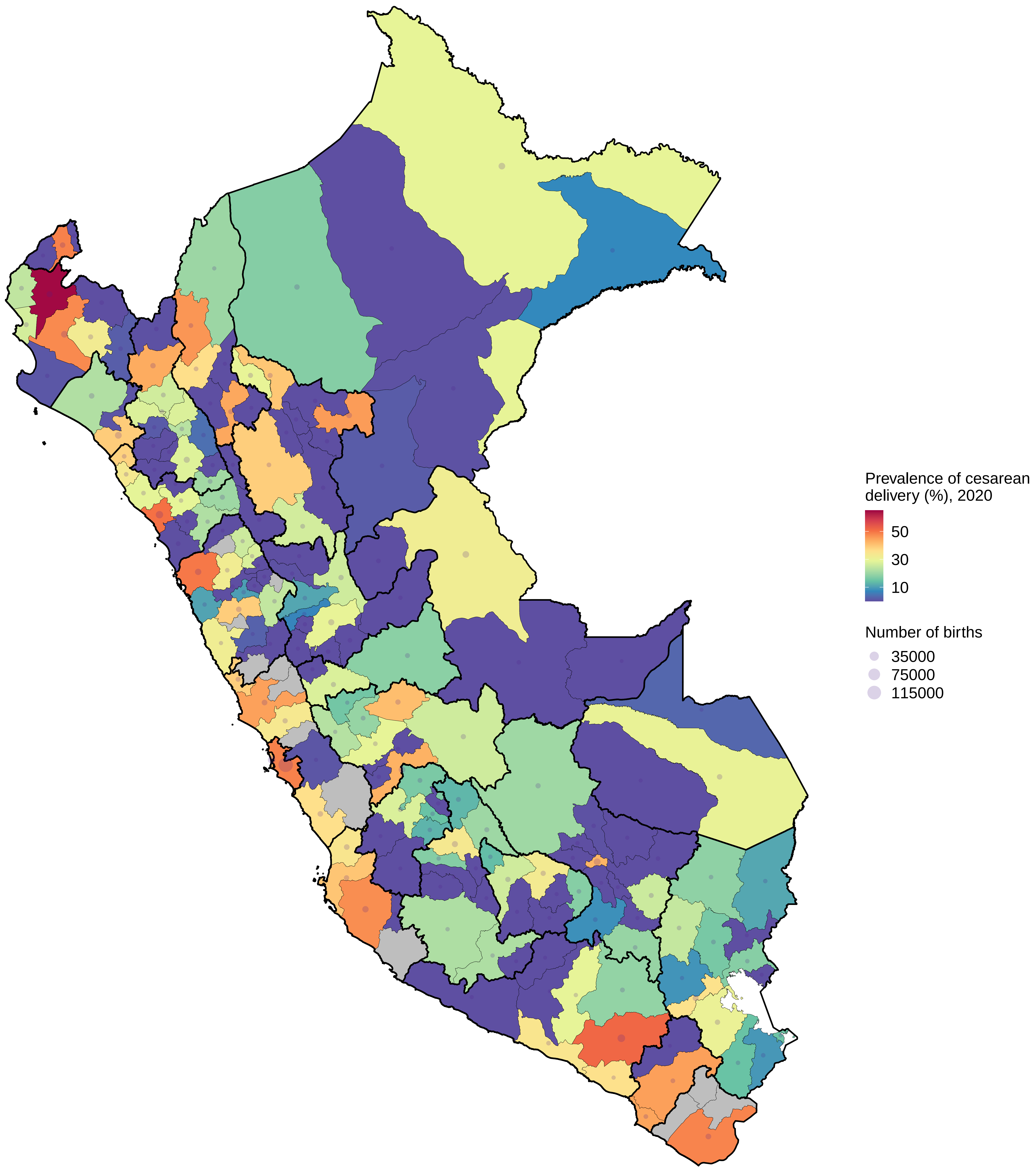
Prevalence of cesarean delivery (%), 2020
50
30
10
Number of births
35000
75000
115000

STROBE Statement—Checklist of items that should be included in reports of ***cohort studies***

	Item No	Recommendation	Page No
**Title and abstract**	1	(*a*) Indicate the study's design with a commonly used term in the title or the abstract<br>(*b*) Provide in the abstract an informative and balanced summary of what was done and what was found	1, 4
**Introduction**			
Background/rationale	2	Explain the scientific background and rationale for the investigation being reported	7
Objectives	3	State specific objectives, including any prespecified hypotheses	7
**Methods**			
Study design	4	Present key elements of study design early in the paper	8-9
Setting	5	Describe the setting, locations, and relevant dates, including periods of recruitment, exposure, follow-up, and data collection	8
Participants	6	(*a*) Give the eligibility criteria, and the sources and methods of selection of participants. Describe methods of follow-up<br>(*b*) For matched studies, give matching criteria and number of exposed and unexposed	9
Variables	7	Clearly define all outcomes, exposures, predictors, potential confounders, and effect modifiers. Give diagnostic criteria, if applicable	8
Data sources/ measurement	8*	For each variable of interest, give sources of data and details of methods of assessment (measurement). Describe comparability of assessment methods if there is more than one group	8
Bias	9	Describe any efforts to address potential sources of bias	13
Study size	10	Explain how the study size was arrived at	8
Quantitative variables	11	Explain how quantitative variables were handled in the analyses. If applicable, describe which groupings were chosen and why	9
Statistical methods	12	(*a*) Describe all statistical methods, including those used to control for confounding<br>(*b*) Describe any methods used to examine subgroups and interactions<br>(*c*) Explain how missing data were addressed<br>(*d*) If applicable, explain how loss to follow-up was addressed<br>(*e*) Describe any sensitivity analyses	9-10
**Results**			
Participants	13*	(a) Report numbers of individuals at each stage of study—eg numbers potentially eligible, examined for eligibility, confirmed eligible, included in the study, completing follow-up, and analysed<br>(b) Give reasons for non-participation at each stage<br>(c) Consider use of a flow diagram	11
Descriptive data	14*	(a) Give characteristics of study participants (eg demographic, clinical, social) and information on exposures and potential confounders<br>(b) Indicate number of participants with missing data for each variable of interest<br>(c) Summarise follow-up time (eg, average and total amount)	11, 12
Outcome data	15*	Report numbers of outcome events or summary measures over time	11,12

Main results	16	(*a*) Give unadjusted estimates and, if applicable, confounder-adjusted estimates and their precision (eg, 95% confidence interval). Make clear which confounders were adjusted for and why they were included<br>(*b*) Report category boundaries when continuous variables were categorized<br>(*c*) If relevant, consider translating estimates of relative risk into absolute risk for a meaningful time period	11,12
Other analyses	17	Report other analyses done—eg analyses of subgroups and interactions, and sensitivity analyses	12
**Discussion**			
Key results	18	Summarise key results with reference to study objectives	13
Limitations	19	Discuss limitations of the study, taking into account sources of potential bias or imprecision. Discuss both direction and magnitude of any potential bias	13,14
Interpretation	20	Give a cautious overall interpretation of results considering objectives, limitations, multiplicity of analyses, results from similar studies, and other relevant evidence	13,14
Generalisability	21	Discuss the generalisability (external validity) of the study results	14,15
**Other information**			
Funding	22	Give the source of funding and the role of the funders for the present study and, if applicable, for the original study on which the present article is based	3

*Give information separately for exposed and unexposed groups.

Note: An Explanation and Elaboration article discusses each checklist item and gives methodological background and published examples of transparent reporting. The STROBE checklist is best used in conjunction with this article (freely available on the Web sites of PLoS Medicine at http://www.plosmedicine.org/, Annals of Internal Medicine at http://www.annals.org/, and Epidemiology at http://www.epidem.com/). Information on the STROBE Initiative is available at http://www.strobe-statement.org.
