# Supplementary material for: Flat trend of high caesarean section rates in Peru: A pooled analysis of 3,376,062 births from the national birth registry, 2012 to 2020
Source: Lancet Reg Health Am. 2022 Jun 17;12:100293. doi: 10.1016/j.lana.2022.100293 (PMC9378316; doi:10.1016/j.lana.2022.100293)
Supplement: Supplementary file 2 [file mmc2.docx]

***Editorial disclaimer:*** *This translation in Spanish was submitted by the authors and we reproduce it as supplied. It has not been peer reviewed. Our editorial processes have only been applied to the original abstract in English, which should serve as reference for this manuscript.*

**RESUMEN**

**Antecedentes** Las tasas de cesárea nacionales y subnacionales rara vez están disponibles en países de ingresos bajos y medianos para orientar las políticas y las intervenciones. Nuestro objetivo fue describir las tasas de cesárea a nivel nacional y subnacional en Perú (2012-2020).

**Métodos** Basándonos en el registro nacional de nacimientos del Perú, cuantificamos las tasas de cesárea a nivel nacional, regional y provincial; también por regiones naturales (costa, sierra y selva). Usando datos a nivel individual de la madre, estratificamos las tasas de cesárea por nivel educativo, seguro médico y proveedor. Ecológicamente, estudiamos las correlaciones entre las tasas de cesárea y el índice de desarrollo humano (IDH), la altitud sobre el nivel del mar, la proporción de la población que vive en la pobreza y la proporción de la población rural.

**Hallazgos** A nivel nacional la tasa disminuyó ligeramente del 2012 (39·7%) al 2020 (38·0%).

En el periodo de estudio, se observó una creciente brecha en las tasas de cesáreas entre la Costa que mostró tasas más altas y las otras regiones naturales que mostraron tasas más bajas. Las tasas en la mayoría de las 25 regiones mostraron una tendencia plana, particularmente en los últimos cuatro años y algunas provincias mostraron tasas de cesáreas muy bajas. Las tasas fueron más altas en madres con educación superior y en usuarias de seguros de salud privada. Un IDH más alto, establecimientos de salud ubicados a menor altitud, menor pobreza y urbanización se correlacionaron positivamente con tasas más altas de cesáreas.

**Interpretación** Las tasas de cesáreas en Perú están por encima de las recomendaciones internacionales. Se encontró grandes diferencias por región natural, provincia y estatus socioeconómico de la madre. Esfuerzos adicionales son necesarios para alcanzar las tasas de cesáreas recomendadas.
